# Deletion of the Polycomb-Group Protein EZH2 Leads to Compromised Self-Renewal and Differentiation Defects in Human Embryonic Stem Cells

**DOI:** 10.1016/j.celrep.2016.11.032

**Published:** 2016-12-06

**Authors:** Adam Collinson, Amanda J. Collier, Natasha P. Morgan, Arnold R. Sienerth, Tamir Chandra, Simon Andrews, Peter J. Rugg-Gunn

**Affiliations:** 1 Epigenetics Programme, The Babraham Institute, Cambridge CB22 3AT, UK; 2 Wellcome Trust – Medical Research Council Stem Cell Institute, University of Cambridge, Cambridge CB2 1QR, UK; 3 Bioinformatics Group, The Babraham Institute, Cambridge CB22 3AT, UK; 4 Centre for Trophoblast Research, University of Cambridge, Cambridge CB2 3EG, UK

**Keywords:** pluripotency, differentiation, epigenetics, histone methylation

## Abstract

Through the histone methyltransferase EZH2, the Polycomb complex PRC2 mediates H3K27me3 and is associated with transcriptional repression. PRC2 regulates cell-fate decisions in model organisms; however, its role in regulating cell differentiation during human embryogenesis is unknown. Here, we report the characterization of *EZH2*-deficient human embryonic stem cells (hESCs). H3K27me3 was lost upon *EZH2* deletion, identifying an essential requirement for EZH2 in methylating H3K27 in hESCs, in contrast to its non-essential role in mouse ESCs. Developmental regulators were derepressed in *EZH2*-deficient hESCs, and single-cell analysis revealed an unexpected acquisition of lineage-restricted transcriptional programs. *EZH2*-deficient hESCs show strongly reduced self-renewal and proliferation, thereby identifying a more severe phenotype compared to mouse ESCs. *EZH2*-deficient hESCs can initiate differentiation toward developmental lineages; however, they cannot fully differentiate into mature specialized tissues. Thus, *EZH2* is required for stable ESC self-renewal, regulation of transcriptional programs, and for late-stage differentiation in this model of early human development.

## Highlights

• Comprehensive examination of *EZH2* function in human ESC regulation
• *EZH2* deficiency causes lineage-restricted derepression of developmental regulators
• More severe self-renewal and growth defects in *EZH2*-deficient hESCs than in mESCs
• *EZH2*-deficient hESCs can differentiate to early lineages but cannot form mature tissues

## Graphical Abstract

**Figure:**
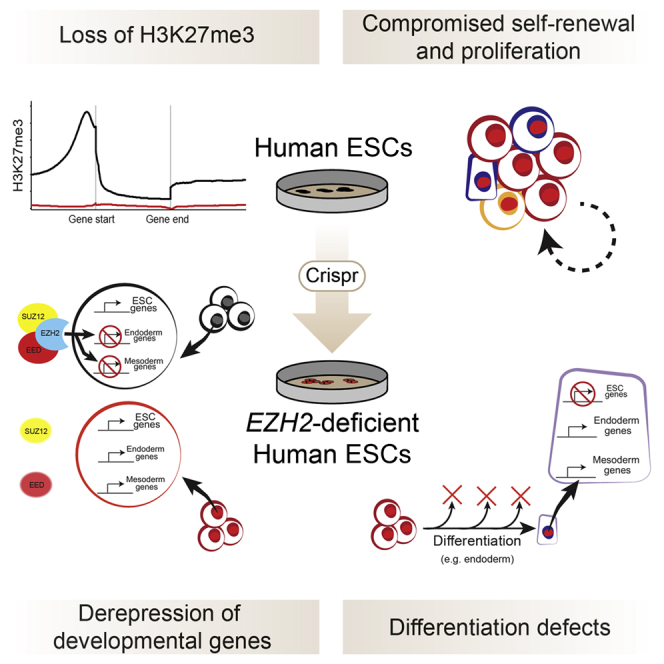

## Teaser

Collinson et al. use *EZH2*-deficient human ESCs to demonstrate the broad conservation of Polycomb-group protein function in controlling cell-fate decisions and transcriptional programs during early human development. The authors also uncover unexpected human-specific differences that result in a more severe self-renewal and proliferation phenotype than that of PRC2-deficient mouse ESCs.

## Introduction

Polycomb-group (PcG) proteins are epigenetic repressors of transcriptional programs and maintain cellular identity during development, differentiation, and disease (Di Croce and Helin, 2013, Pasini and Di Croce, 2016, Pietersen and van Lohuizen, 2008, Schuettengruber and Cavalli, 2009, Surface et al., 2010). PcG proteins form two well-characterized and biochemically distinct chromatin-modifying complexes that are termed Polycomb Repressive Complex 1 and 2 (PRC1 and PRC2). PRC1 catalyzes histone H2A lysine 119 ubiquitination through the activity of the E3 ligases RING1A and RING1B (Müller and Verrijzer, 2009, Wang et al., 2004). PRC2 is composed of the core proteins EZH2, EED, and SUZ12, together with RBAP46/48 and several other accessory subunits, and is responsible for catalyzing di- and trimethylation on histone H3 lysine 27 (H3K27me2/3) (Cao et al., 2002, Czermin et al., 2002, Kuzmichev et al., 2002, Margueron and Reinberg, 2011, Müller et al., 2002). EZH2 is a SET-domain containing histone methyltransferase and is the catalytic subunit of PRC2. EED and SUZ12 are required for substrate recognition, complex stability and for promoting the enzymatic activity of EZH2 (Cao and Zhang, 2004, Nekrasov et al., 2005, Pasini et al., 2004, Tie et al., 2007).

Genome-wide studies in mouse and human embryonic stem cells (ESCs) have shown that PRC2 and H3K27me3 occupy the promoters of many developmental regulators that are important for cell differentiation and lineage specification (Azuara et al., 2006, Bernstein et al., 2006, Boyer et al., 2006, Bracken et al., 2006, Lee et al., 2006, Mikkelsen et al., 2007, Pan et al., 2007, Zhao et al., 2007). This distribution of chromatin marks led to the concept that PRC2 may contribute to the maintenance of pluripotency by keeping developmental regulators transcriptionally repressed, while enabling the genes to be rapidly activated upon suitable differentiation cues and stimuli. Despite a central position within the regulatory framework, however, PRC2 is dispensable for the maintenance of undifferentiated mouse ESCs, as the deletion of PRC2 components, including *Ezh2*, has little effect on their morphology, self-renewal, or proliferation, although a subset of PRC2 target genes are modestly derepressed (Chamberlain et al., 2008, Leeb et al., 2010, Pasini et al., 2007, Riising et al., 2014, Shen et al., 2008). H3K27me3 levels are globally reduced in *Ezh2*-deficient mouse ESCs; however, developmental regulators retain H3K27me3 at their gene promoters and are transcriptionally repressed (Shen et al., 2008). In this context, the Ezh2 homolog, Ezh1, forms a noncanonical PRC2 complex that is able to trimethylate H3K27 at target gene promoters and maintains transcriptional repression through methylation-dependent and potentially methylation-independent pathways (Margueron et al., 2008, Shen et al., 2008).

PRC2 deficiency has a more significant impact on mouse ESCs upon their differentiation, with defects in the repression of pluripotency networks and in the failure to fully activate differentiation transcriptional programs. This aberrant gene regulation results in impaired differentiation and proliferation (Chamberlain et al., 2008, Pasini et al., 2007, Shen et al., 2008). Further underscoring the critical role of PRC2 in directing differentiation programs, all three PRC2 core components (*Ezh2*, *Eed*, and *Suz12*) are essential for early mouse development, as loss-of-function mutant embryos initiate but fail to complete gastrulation and die between embryonic days 7 and 9 (Faust et al., 1995, O’Carroll et al., 2001, Pasini et al., 2004). The mutant phenotype is associated with mis-expression of lineage-specifying genes, decreased cell proliferation and an increased level of apoptosis (Pasini et al., 2004).

The well-conserved binding profiles of PRC2 components and H3K27me3 in human ESCs (hESCs) at the promoters of developmental regulators raises the possibility that PRC2 may also have an important role in controlling hESC pluripotency and differentiation (Gifford et al., 2013, Ku et al., 2008, Pan et al., 2007, Zhao et al., 2007). Moreover, coordinated changes to the epigenome, including H3K27me3 localization, occur upon differentiation of hESCs and are thought to be essential for lineage specification and memory of cellular identity, as they are in *Drosophila* and the mouse (Gifford et al., 2013, Xie et al., 2013). However, no functional studies of PRC2 in hESC regulation and early-stage differentiation have been reported to date. Human pluripotent cells represent a unique model in which to study human development and provide a platform for producing a source of differentiated cells relevant for basic and applied research. In addition, mouse and human ESCs are known to represent different pluripotent states and may therefore rely on different epigenetic pathways to confer their ability to self-renew and differentiate (Nichols and Smith, 2009, Rossant, 2015). Understanding the key epigenetic mechanisms that underpin hESCs is therefore a priority.

Here, we report the generation and characterization of *EZH2*-deficient hESCs. Our findings demonstrate that EZH2 is required to maintain the transcriptional repression of developmental regulators and for cells to undergo late-stage cell differentiation, thereby revealing the broad conservation of PRC2 function in this model of early human development. We also identify unexpected human-specific differences such as the essential requirement in hESC for EZH2 to maintain PRC2 stability and retain promoter-localized H3K27me3, which is in contrast to its non-essential role in mouse ESCs. In addition, self-renewal and proliferation are also perturbed to a greater extent in *EZH2*-deficient hESCs, as compared to *Ezh2*-deficient mouse ESCs. Our study therefore provides a comprehensive characterization of PRC2 function in hESCs, thereby providing a new platform to investigate the role of histone methylation in regulating the genome during human development and stem cell differentiation.

## Results

### Targeted Deletion of *EZH2* in hESCs

**Figure 1:**
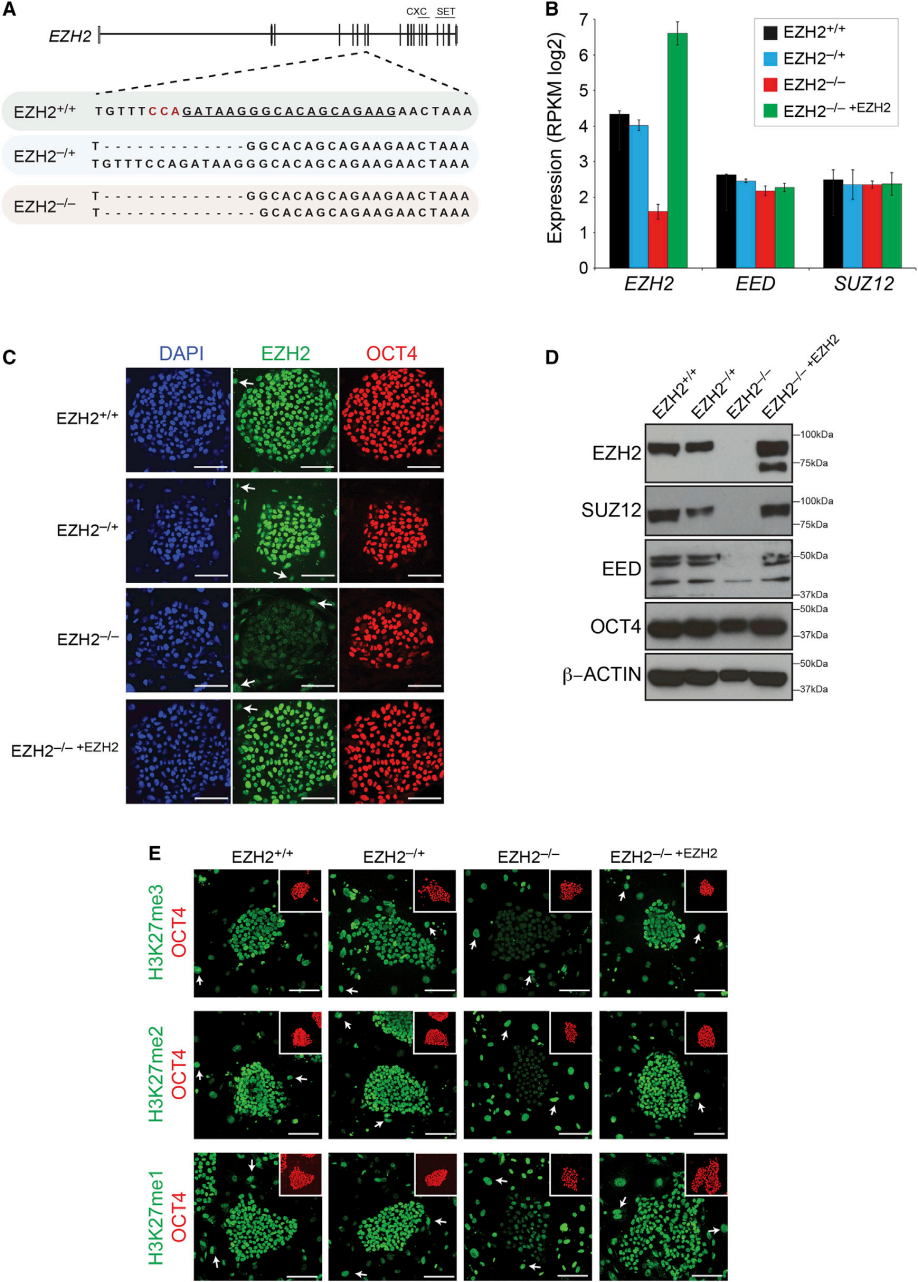
Targeted Deletion of *EZH2* in hESCs (A) Overview of *EZH2* structure and targeting strategy. Exons encoding CXC and SET domains are indicated. The gRNA sequence is underlined and protospacer adjacent motif highlighted in red. DNA sequence of the deletions in one *EZH2*^–/+^ ESC line and one *EZH2*^–/–^ ESC line is shown for both alleles. Mutation causes frameshift and premature stop codon. An additional line is shown in Figures S1 and S2. (B) mRNA expression levels from RNA-seq data revealing *EZH2*, *EED,* and *SUZ12* transcript levels in *EZH2*^+/+^, *EZH2*^–/+^, *EZH2*^–/–^, and *EZH2*^–/–^^+*EZH2*^ ESCs. Data show mean ± SD; n = 3 biological replicates. (C) Immunofluorescent microscopy of colonies from *EZH2*^–/–^ ESCs and control ESCs. This analysis reveals a strong reduction in EZH2 levels in *EZH2*^–/–^ ESCs. The antibody was raised against a C-terminal epitope of EZH2; similar results were obtained using an alternative antibody raised against the N-terminal of EZH2 (Figure S2E). OCT4 expression indicates undifferentiated cells within a hESC colony. Arrows point to MEF. Scale bars, 100 μm. (D) EZH2, SUZ12, and the main isoform of EED are undetectable in *EZH2*^–/–^ ESCs by western blot analysis and are restored upon expression of a DOX-induced *EZH2* transgene. β-ACTIN is the loading control. Mass is in kilodaltons. (E) H3K27me3 and H3K27me2 levels are reduced to background levels, and H3K27me1 levels are partially reduced, in *EZH2*^–/–^ ESCs. OCT4 expression in inset indicates undifferentiated ESCs within the field of view. Arrows point to MEF. Scale bars, 100 μm.

To investigate the role of *EZH2* in human pluripotency and differentiation, we used CRISPR/Cas9 to disrupt *EZH2* in hESCs. A guide RNA (gRNA) designed to target an early exon within all known *EZH2* isoforms was nucleofected with *Cas9* into the H9 hESC line (Figures 1A and S1A). Individual colonies were isolated, expanded, and analyzed by Sanger DNA sequencing. The efficiency of disrupting the target sequence within the *EZH2* coding region was high, with ∼35% clonal lines containing a mutation on one allele (*EZH2*^–/+^). However, no homozygous cell lines were obtained out of 110 screened lines. This result provided a first indication that *EZH2*-deficient hESCs may be compromised relative to *EZH2*-containing cells when plated as single cells at clonal density. To overcome this apparent defect, we introduced a doxycycline (DOX)-inducible *EZH2* transgene using piggyBac transposition into an *EZH2*^–/+^ line and re-targeted the cells with *EZH2* gRNA and *Cas9* in the presence of DOX. Using this strategy, we obtained several *EZH2* homozygous lines (*EZH2*^–/–^; Figures 1A, S1B, and S1C). Once the *EZH2*^–/–^ lines were isolated and established, they could be maintained without DOX-induced *EZH2* expression. Although we did not detect any indication that the DOX-inducible plasmid was leaky in the absence of DOX, to rule out the possibility of low-level *EZH2* expression, we transiently transfected *EZH2*^–/–^ ESCs with piggyBac transposase and obtained stable *EZH2*^–/–^ lines with all copies of the *EZH2* transgene removed (Figures S2A and S2B).

RNA expression analysis confirmed that *EZH2* transcripts were lower in *EZH2*^–/–^ ESCs compared to parental *EZH2*^+/+^ and *EZH2*^–/+^ lines (Figures 1B and S2C). Moreover, EZH2 protein was undetectable by western blot and by immunofluorescent microscopy using two different antibodies raised against N- and C-terminal epitopes of EZH2 (Figures 1C, 1D, and S2D–S2F). The disruption of *EZH2* was accompanied by the loss of other PRC2 proteins, SUZ12 and EED, despite the presence of unchanged levels of *SUZ12* and *EED* transcripts in *EZH2*^–/–^ ESCs (Figures 1B and 1D). This finding unexpectedly contrasts with *Ezh2*-deficient mouse ESCs where Suz12 and Eed levels are unchanged due to the ability of Ezh1 to form noncanonical PRC2 (Shen et al., 2008) but is consistent with *Suz12*-deficient and *Eed*-deficient mouse ESCs in which PRC2 components are unstable outside of the complex (Pasini et al., 2004, Pasini et al., 2007). In hESCs, therefore, EZH1 is unable to form noncanonical PRC2 despite being present (Figure S2G). *EZH1* transcript and protein levels were largely unchanged upon *EZH2* deletion (Figure S2G). Immunofluorescent microscopy revealed that the loss of *EZH2* led to the reduction of H3K27me3 and H3K27me2 to background levels, and to the partial reduction of H3K27me1 (Figure 1E). Applying DOX to induce ectopic *EZH2* expression in *EZH2*^–/–^ ESCs restored EZH2 and led to the stabilization of SUZ12 and EED proteins, and to the re-establishment of global H3K27 methylation (cells designated herein as *EZH2*^–/–^^+*EZH2*^; Figures 1B–1E).

### Loss of Promoter-Localized H3K27me3 in *EZH2*-Deficient hESCs

**Figure 2:**
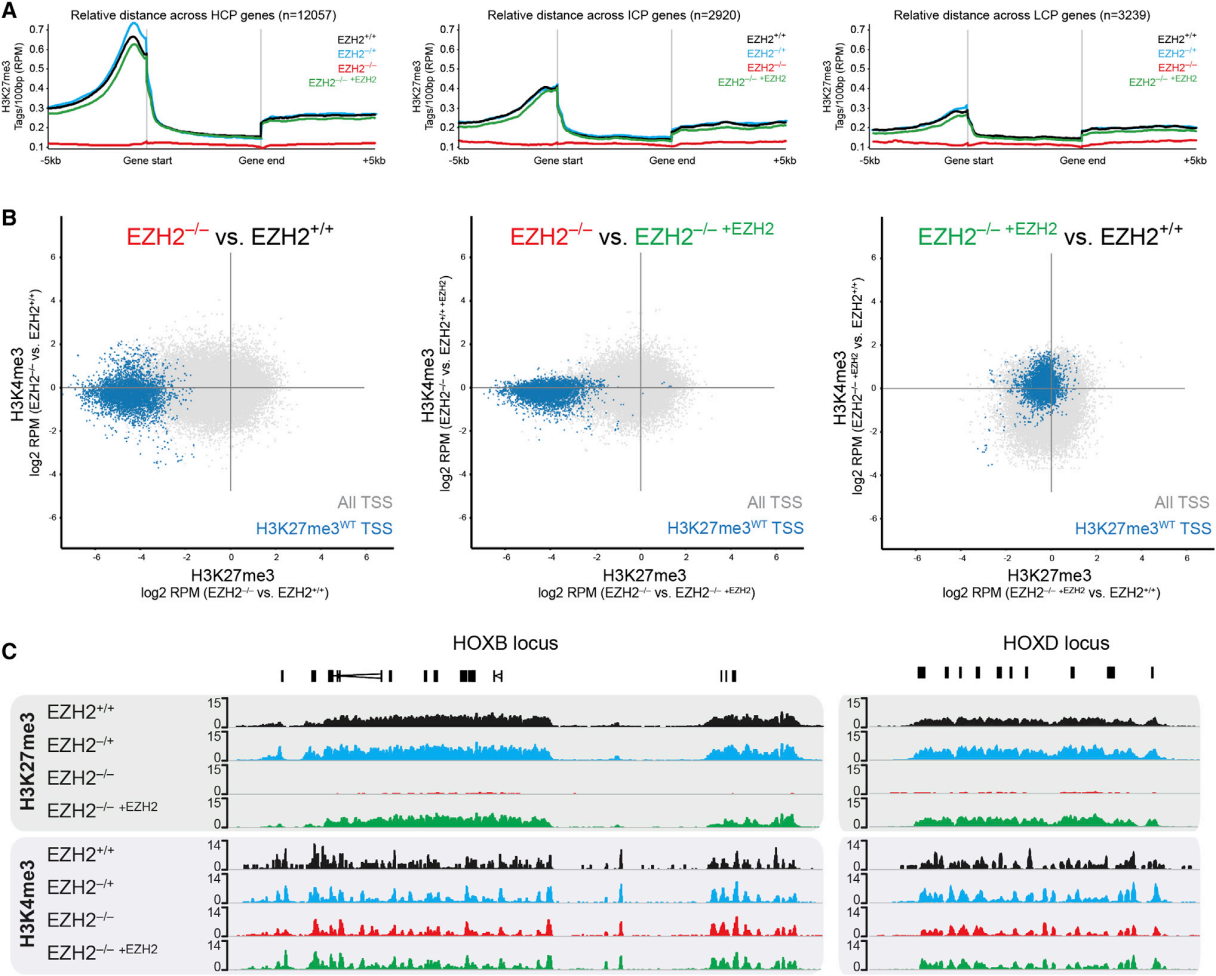
*EZH2* Deficiency in hESCs Results in Loss of H3K27me3 (A) Quantitative trend plot of H3K27me3 normalized ChIP-seq reads over gene body ±5 kb. High CpG (HCP), intermediate CpG (ICP), and low CpG (LCP) promoters are shown separately. (B) Scatterplot of H3K27me3 (x axis) and H3K4me3 (y axis) normalized ChIP-seq reads in *EZH2*^–/–^ relative to *EZH2*^+/+^ (left) and relative to *EZH2*^–/– +*EZH2*^ (center), and *EZH2*^–/– +*EZH2*^ versus *EZH2*^+/+^ (right). All transcriptional start sites (TSS) shown in gray; TSS that are positive for H3K27me3 in *EZH2*^+/+^ ESCs highlighted in blue. Disruption of *EZH2* leads to a strong reduction in H3K27me3 levels at TSS, with little effect on H3K4me3 levels. Expression of a DOX-mediated *EZH2* transgene in the *EZH2*-deficient cells causes restoration of H3K27me3 levels to levels equivalent to *EZH2*^+/+^. (C) ChIP-seq tracks of *HOXB* (left) and *HOXD* (right) loci illustrate the loss of H3K27me3 in *EZH2*^–/–^ ESCs compared to control ESCs. H3K4me3 is relatively unaffected. All ChIP-seq data represent the average of three biological replicates for each cell line. These results were confirmed independently by qPCR analysis of ChIP DNA at several gene promoters (Figure S3C).

To characterize the molecular phenotype of *EZH2*-deficient hESCs, we profiled genome-wide histone methylation by native chromatin immunoprecipitation combined with high-throughput sequencing (chromatin immunoprecipitation sequencing [ChIP-seq]). Quantitative trend plots of normalized ChIP-seq reads revealed a complete loss of H3K27me3 at all gene promoters in *EZH2*^–/–^ ESCs (Figure 2A). This finding contrasts with the retention of H3K27me3 at the promoters of developmental regulators in *Ezh2*-deficient mouse ESCs (Shen et al., 2008). Confirming the result in hESCs, scatterplot analysis of ∼2,000 promoters that have high levels of H3K27me3 in *EZH2*^+/+^ ESCs (H3K27me3^WT^) revealed a loss of H3K27me3 in *EZH2*^–/–^ ESCs (Figure 2B). The majority of H3K27me3^WT^ promoters have histone H3 lysine 4 trimethylation (H3K4me3) in hESCs (Pan et al., 2007, Zhao et al., 2007), and H3K4me3 levels were largely unaffected by *EZH2* disruption (Figure 2B). ChIP-seq tracks for two example loci, *HOXB* and *HOXD*, illustrate the loss of H3K27me3 across the domains in *EZH2*^–/–^ ESCs (Figure 2C). Comparison of H3K27me3 between *EZH2*^+/+^ and *EZH2*^–/– +*EZH2*^ cells revealed highly similar profiles, demonstrating that histone patterns are appropriately re-established upon EZH2 restoration (Figures 2A–2C). Interestingly, there was a modest increase in histone H3 lysine 27 acetylation (H3K27ac) levels at H3K27me3^WT^ promoters in *EZH2*^–/–^ ESCs, supporting a potential antagonism between H3K27 acetylation and trimethylation that has also been observed in other contexts (Ferrari et al., 2014, Gehani et al., 2010, Jung et al., 2010, Pasini et al., 2010, Schoenfelder et al., 2015) (Figure S3A). Last, an alternative ChIP-seq analysis strategy of genome binning confirmed a loss of H3K27me3 sequencing reads across the genome of *EZH2*^–/–^ ESCs, further reinforcing the key finding that H3K27me3 levels are depleted upon deletion of *EZH2* in hESCs (Figure S3B). Together, these results demonstrate that EZH2 is the main functional H3K27me2/3 methyltransferase in hESCs.

### *EZH2* Deficiency Causes Transcriptional Derepression of Key Developmental Genes

**Figure 3:**
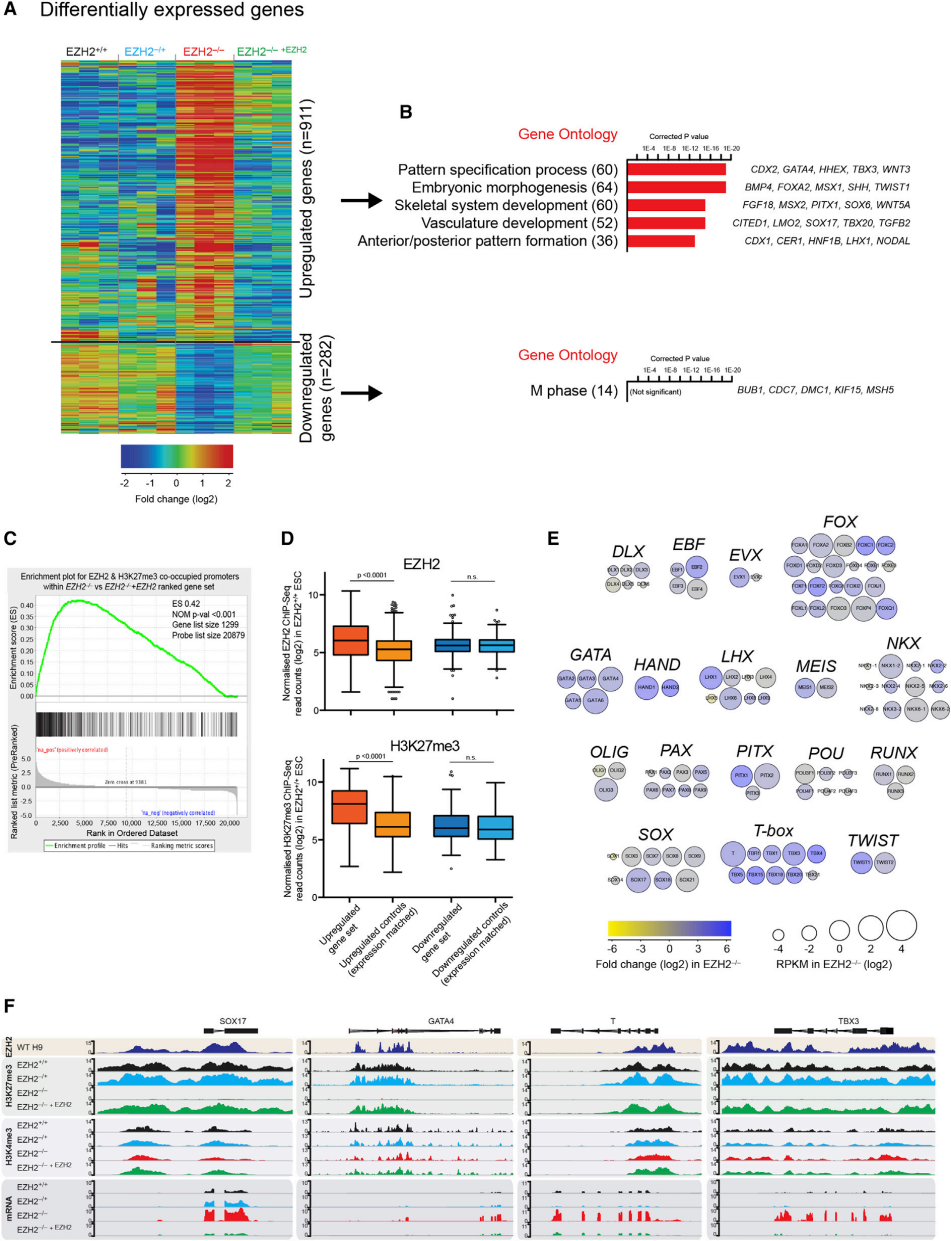
Genes Encoding Developmental Regulators Are Transcriptionally Derepressed in *EZH2*-Deficient hESCs (A) RNA-seq heatmap for *EZH2*^–/–^ ESCs and control ESCs (three biological replicates per line). Shown are all differentially expressed genes between *EZH2*^–/–^and *EZH2*^–/–^^+*EZH2*^ ESCs. (B) Top GO terms of differentially expressed gene sets. Numbers of genes are shown; example genes within each GO category are listed (right). Corrected p values were calculated using a modified Fisher’s exact test followed by Bonferroni’s multiple comparison test. (C) Gene set enrichment analysis of PRC2 targets (n = 1,299; defined by high EZH2 and H3K27me3 promoter-localized ChIP-seq values in *EZH2*^+/+^ ESCs) in genes that have been ranked according to their fold change in transcription between *EZH2*^–/–^ ESCs and *EZH2*^–/–^^+*EZH2*^ ESCs. The positive enrichment score (ES) reveals that genes selectively derepressed in the absence of *EZH2* are enriched in PRC2 targets (p < 0.001; Kolmogorov-Smirnov statistic). (D) Genes within the upregulated category have higher levels of promoter-localized EZH2 (upper) and H3K27me3 (lower) in EZH2^+/+^ ESCs compared to an expression-matched set of genes and to downregulated genes. Data were compared using a Kruskal-Wallis test followed by Dunn’s multiple comparison test. (E) Genes encoding developmental regulators are transcriptionally derepressed in *EZH2*-deficient ESCs. A subset of direct EZH2 target genes is depicted as family groups. The color of each circle represents the log2 fold change in *EZH2*^–/–^ ESCs relative to *EZH2*^–/–^^+*EZH2*^ ESCs. The size of each circle represents the expression value of the gene in *EZH2*^–/–^ cells. A similar pattern of target gene derepression is observed when comparing *EZH2*^–/–^ ESCs with *EZH2*^+/+^ ESCs (Figure S4C). (F) ChIP-seq and mRNA-seq tracks of four genes encoding key developmental regulators illustrate the association between loss of H3K27me3 and transcriptional upregulation in *EZH2*^–/–^ ESCs.

We next performed RNA sequencing (RNA-seq) to investigate the impact of loss of EZH2 and associated H3K27me3 on gene expression. The assays were carried out on samples that were flow-sorted using the hESCs cell-surface marker SSEA4 to ensure that we compared between equivalent cell populations (Figure S4A). The majority of genes were not altered transcriptionally by *EZH2* disruption, but 911 genes were significantly upregulated and 282 genes were significantly downregulated in *EZH2*^–/–^ ESCs compared to *EZH2*^–/–^^+*EZH2*^ ESCs (p < 0.05; Figures 3A and S4B). Gene ontology (GO) analysis of the upregulated gene set identified categories associated with developmental and cellular differentiation, including pattern specification, embryonic morphogenesis, and tissue formation (Figure 3B). The upregulated group was significantly enriched for genes with EZH2 and H3K27me3 occupancy in *EZH2*^+/+^ ESCs and are thus expected to be sensitive to *EZH2* disruption (Figures 3C and 3D). Notably, not all genes with EZH2-bound promoters were mis-regulated, suggesting that secondary events may be required for this group of genes to initiate transcriptional change in response to the loss of EZH2. In addition, a subset of upregulated genes (enriched for signaling and adhesion GO terms) are likely to be regulated indirectly as they are not PRC2 targets in hESCs. No GO categories were significantly enriched in the downregulated gene set, although of note, the top category was associated with the regulation of M-phase and indicate the decreased transcription of genes typically expressed in mitosis (Figure 3B).

We examined transcriptional changes in a set of ∼100 classical developmental regulators that have strong EZH2 promoter occupancy, including genes within the FOX, GATA, LHX, T-box, and SOX families (Lee et al., 2006). A clear pattern emerges from this analysis: nearly all genes within this class of developmental regulator showed transcriptional derepression in the absence of *EZH2* (Figures 3E and S4C). Gene derepression did not become more prevalent upon continued passaging, and therefore the *EZH2*^–/–^ ESCs retained a similar transcriptional profile over time. ChIP-seq and RNA-seq tracks for several example genes, including *SOX17*, *GATA4*, *T*, and *TBX3*, illustrate the absence of promoter H3K27me3 and an associated increased transcript level in *EZH2*^–/–^ ESCs (Figure 3F). We further showed that small hairpin RNA (shRNA)-mediated depletion of *EZH2* causes derepression of developmental regulators in two additional human pluripotent stem cell lines (WIBR3 and FiPS; Figure S4D). We conclude that the deletion of *EZH2* in hESCs leads to the loss of H3K27me3 and to the transcriptional derepression of genes that encode developmental regulators, thereby positioning EZH2 as a key factor in controlling the transcriptome of human cell types during early development.

### Single-Cell Transcriptional Analysis Reveals Gene Mis-regulation Profiles

**Figure 4:**
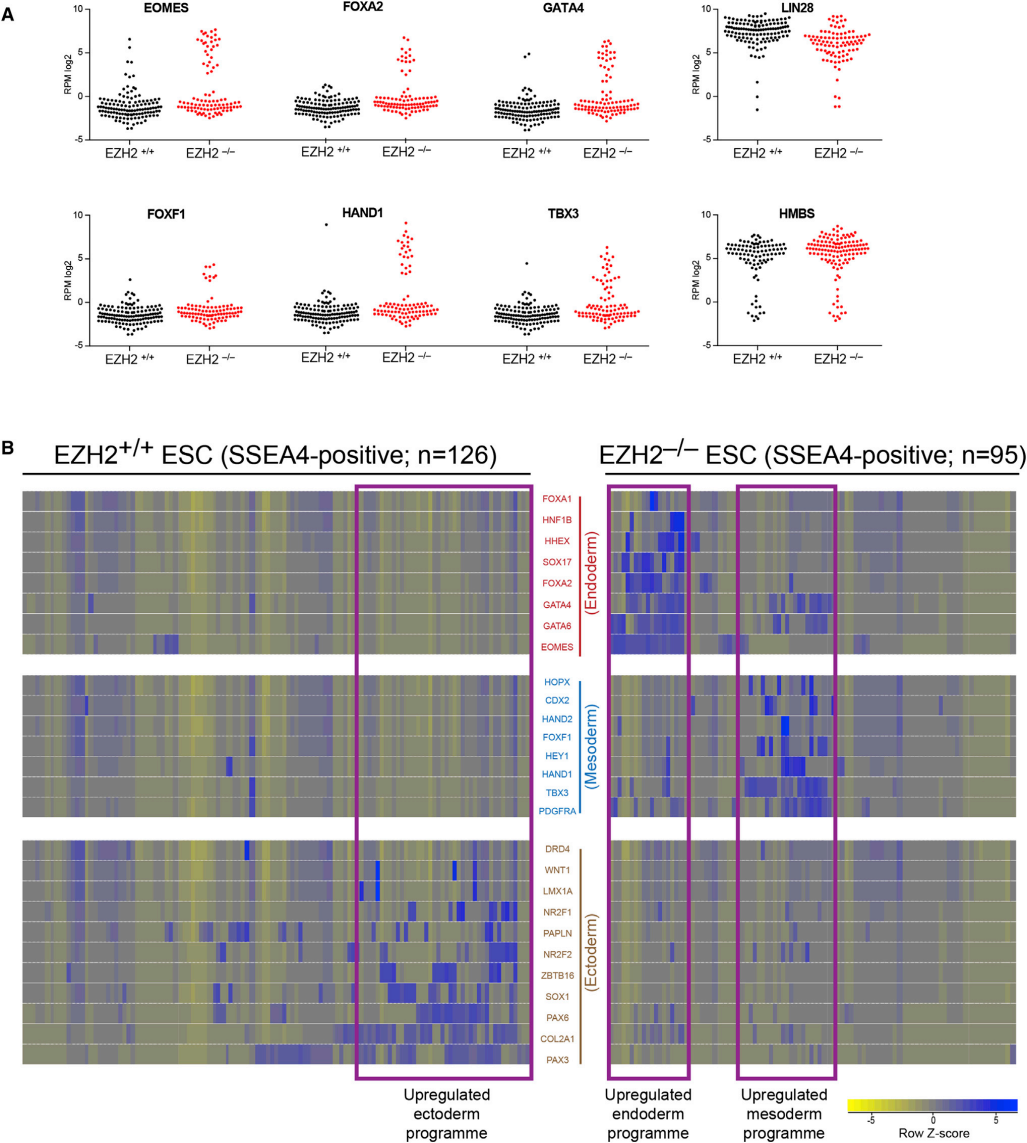
Transcriptional Derepression Occurs Predominantly within Discrete Lineage-Specific Programs (A) Single-cell RNA-seq expression levels for six example PRC2-target genes in *EZH2*^–/–^ ESCs and *EZH2*^+/+^ ESCs, where each dot represent the results from a single cell. A pluripotency gene (*LIN28*) and housekeeping gene (*HMBS*) are shown for comparison. Robust upregulation of PRC2-target genes occurs in a subset of *EZH2*^–/–^ ESCs. (B) Heatmap of single-cell RNA-seq expression for *EZH2*^–/–^ ESCs (right) and *EZH2*^+/+^ ESCs (left). Each column represents an individual cell. Each row represents an individual gene, grouped into three clusters corresponding to endoderm, mesoderm, and ectoderm cell lineages. Shown are PRC2-target genes from within the hESC scorecard assay, which is an assay that can classify differentiated cell lineages (Bock et al., 2011). Subsets of cells (boxed in purple) tend to mis-express many genes from within one lineage but rarely mis-express multiple genes derived from more than one lineage.

To investigate more precisely the transcriptional mis-regulation and cell-to-cell variability in response to *EZH2* deficiency, we performed single-cell RNA-seq on individual SSEA4-positive, flow-sorted *EZH2*^–/–^ and *EZH2*^+/+^ ESCs. The results show that a subset of *EZH2*^–/–^ ESCs strongly upregulated EZH2-target genes, but individual genes are not robustly derepressed in most cells examined (Figure 4A). Unexpectedly, clustering of the data suggested that gene derepression occurs predominately within discrete transcriptional programs, such that individual *EZH2*^–/–^ ESCs have upregulated multiple genes associated with a particular cell lineage but rarely show strong signatures derived from several cell lineages (Figure 4B). In particular, *EZH2*^–/–^ ESCs show lineage biases toward endoderm and mesoderm, but not to ectoderm. This response may be constrained by the hESC culture environment due to the activities of FGF and Activin/Nodal signaling within the media, which are known to promote endoderm and mesoderm specification and suppress ectoderm differentiation (Pauklin and Vallier, 2015). Together, these results reveal that the depletion of EZH2 does not cause global EZH2-target gene derepression in all ESCs, as might be predicted from cell population studies. Rather, its loss leads to the mis-regulation of subsets of genes and to the acquisition of lineage-restricted transcriptional programs.

### *EZH2* Disruption Causes Self-Renewal and Proliferation Defects in hESCs

**Figure 5:**
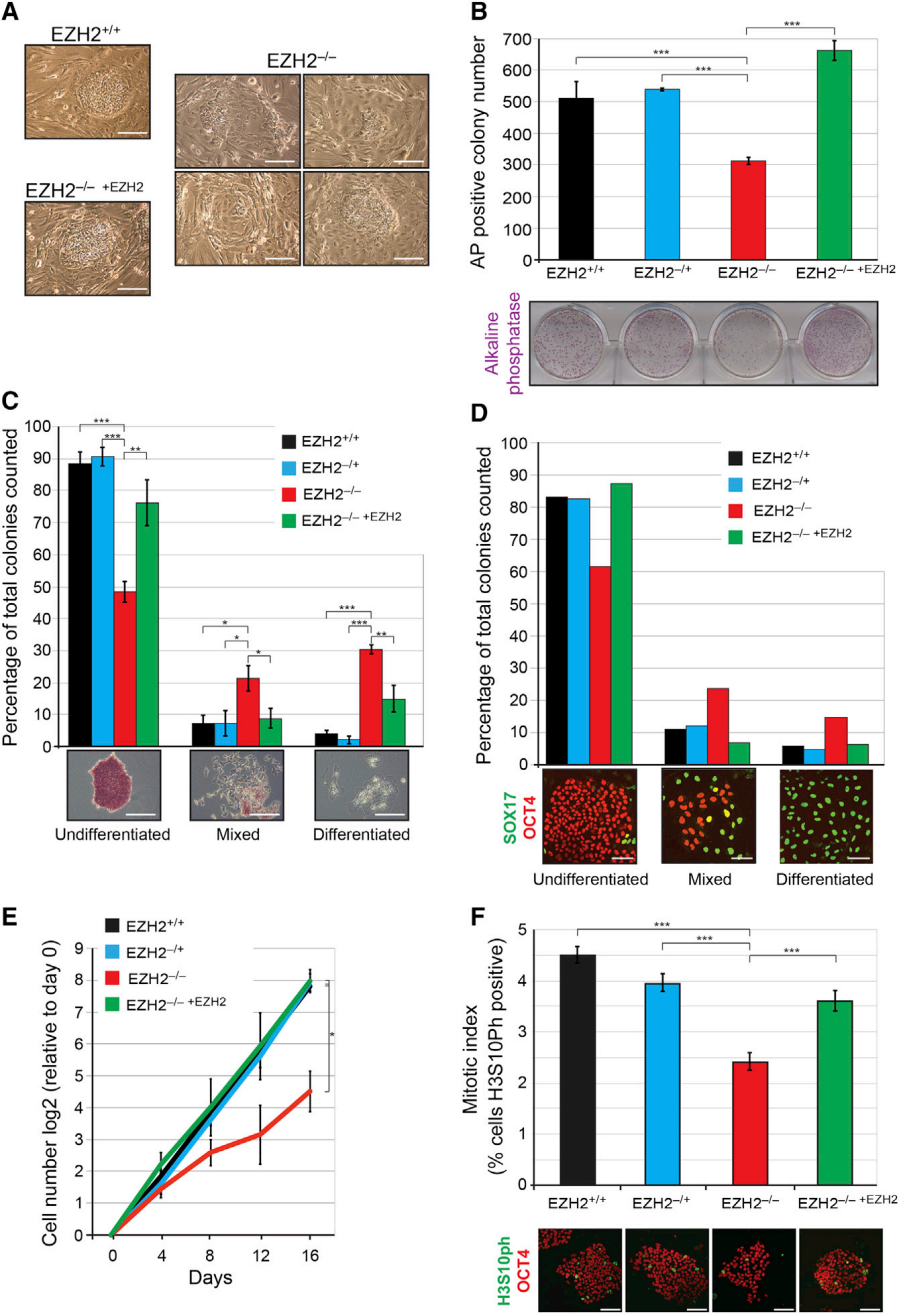
*EZH2*-Deficient hESCs Are Compromised in Self-Renewal and Proliferation (A) Phase contrast images show representative colonies of *EZH2*^+/+^, *EZH2*^–/–^, and *EZH2*^–/–^^+*EZH2*^ ESC lines. Note the variable morphology of *EZH2*-deficient colonies. Scale bars, 100 μm. (B) *EZH2*^–/–^ ESCs show reduced ESC colony formation when plated as single SSEA4-positive cells at low density (6,000 cells seeded per well). Data show mean ± SD; n = 3 biological replicates. Data were compared using a one-way ANOVA followed by Bonferroni’s multiple comparison test (^∗∗∗^p < 0.0005). Representative AP staining is shown underneath. (C) *EZH2*^–/–^ ESCs have reduced capacity to self-renew when plated at clonal density. ESC colonies were categorized as undifferentiated, mixed, or differentiated based on AP activity; examples shown underneath. Data show mean ± SD; n = 3 biological replicates. Over 150 colonies were scored for each cell line. Data were compared using a one-way ANOVA followed by Bonferroni’s multiple comparison test (^∗∗∗^p < 0.0005; ^∗∗^p < 0.005; ^∗^p < 0.05). Scale bars, 100 μm. (D) Immunofluorescent microscopy for OCT4 (undifferentiated marker) and SOX17 (early differentiation marker) reveals an increased prevalence for mixed and fully differentiated colonies in *EZH2*^–/–^ compared to control ESC lines. Representative images are shown underneath. Over 100 colonies were scored for each cell line. Scale bars, 100 μm. (E) Growth curve over 16 days reveals a significant proliferation defect in *EZH2*^–/–^ ESCs compared to control ESCs. Data show mean ± SD; n = 3 biological replicates. Data were compared between *EZH2*^–/–^ ESCs and each control ESC line using one-way ANOVA followed by Bonferroni’s multiple comparison test (^∗^p < 0.05 for each comparison). (F) Mitotic index was calculated for each ESC line by dividing the number of H3S10ph-positive cells by the total number of cells within a colony. The analysis was restricted to undifferentiated colonies (determined by OCT4 expression) of similar size in order to control for potential differences in cell state. Over 1,000 cells were scored for each cell line. Data show mean ± SD; n = 3 biological replicates. Data were compared using a one-way ANOVA followed by Bonferroni’s multiple comparison test (^∗∗∗^p < 0.0005). Scale bars, 100 μm.

Undifferentiated *EZH2*-deficient ESCs could be maintained in culture for >50 passages; however, their growth and morphology were severely compromised compared to control lines. *EZH2*^–/–^ colonies were highly variable in appearance with an increased prevalence of flatter cells that are characteristic of spontaneous differentiation (Figure 5A). To compare directly the ability of each hESC line to self-renew, we plated an equal number of SSEA4-positive flow sorted cells at clonal density, and after 7 days we counted the number of colonies that were positive for the undifferentiated hESC marker alkaline phosphatase (AP). We observed an ∼40% reduction in colony number in *EZH2*^–/–^ ESCs compared to control ESCs (Figure 5B).

Diminished colony formation was due to both compromised self-renewal and impaired proliferation. Categorizing *EZH2*^–/–^ ESC colonies based on AP activity patterns revealed an ∼50% reduction in the proportion of undifferentiated colonies in *EZH2*^–/–^ ESCs compared to control ESCs, and an associated increase in the proportion of colonies with a mixed or fully differentiated phenotype (Figure 5C). This was accompanied by a decrease in the proportion of *EZH2*^–/–^ ESC colonies that are entirely OCT4 positive, with an associated increase in the proportion of colonies that are positive for SOX17, a marker of early differentiated cells (Figure 5D). The proportion of undifferentiated and differentiated cells within the *EZH2*^–/–^ ESC cultures was unchanged over passage and was re-established after plating of purified undifferentiated *EZH2*^–/–^ ESCs, further highlighting the unstable nature of these cells.

Cell counts over four passages revealed an ∼50% reduction in cell number in *EZH2*^–/–^ ESCs, revealing that proliferation is significantly reduced in the absence of *EZH2* (Figure 5E). The mitotic index, as determined by the proportion of histone H3 serine 10 phosphorylation (H3S10ph) positive cells, was significantly lower in *EZH2*^–/–^ ESCs compared to control ESCs (Figure 5F). The reduction in mitotic cells within *EZH2*^–/–^ cultures is in agreement with our RNA-seq results, which identify the transcriptional downregulation of genes associated with M-phase (Figure 3B). Of further relevance to this defect is that several negative regulators of the cell cycle, such as *CDKN2A* (encoding p16INK4A and p14ARF) and *CDKN2B* (encoding p15INK4B), were transcriptionally derepressed in *EZH2*-deficient ESCs (Figure S5). These findings are consistent with previous studies in other cell types that have identified a role for EZH2 in controlling the transcription of cell-cycle regulators (Bracken et al., 2003, Bracken et al., 2007, Pasini et al., 2004, Sauvageau and Sauvageau, 2010, Varambally et al., 2002). Together, the results demonstrate that *EZH2*-deficient hESCs are strongly compromised in their ability to self-renew and proliferate, thereby identifying a more severe phenotype compared to mouse ESCs that are deficient for *Ezh2* and other PRC2 proteins.

### Differentiation Defects in *EZH2*-Deficient hESCs

**Figure 6:**
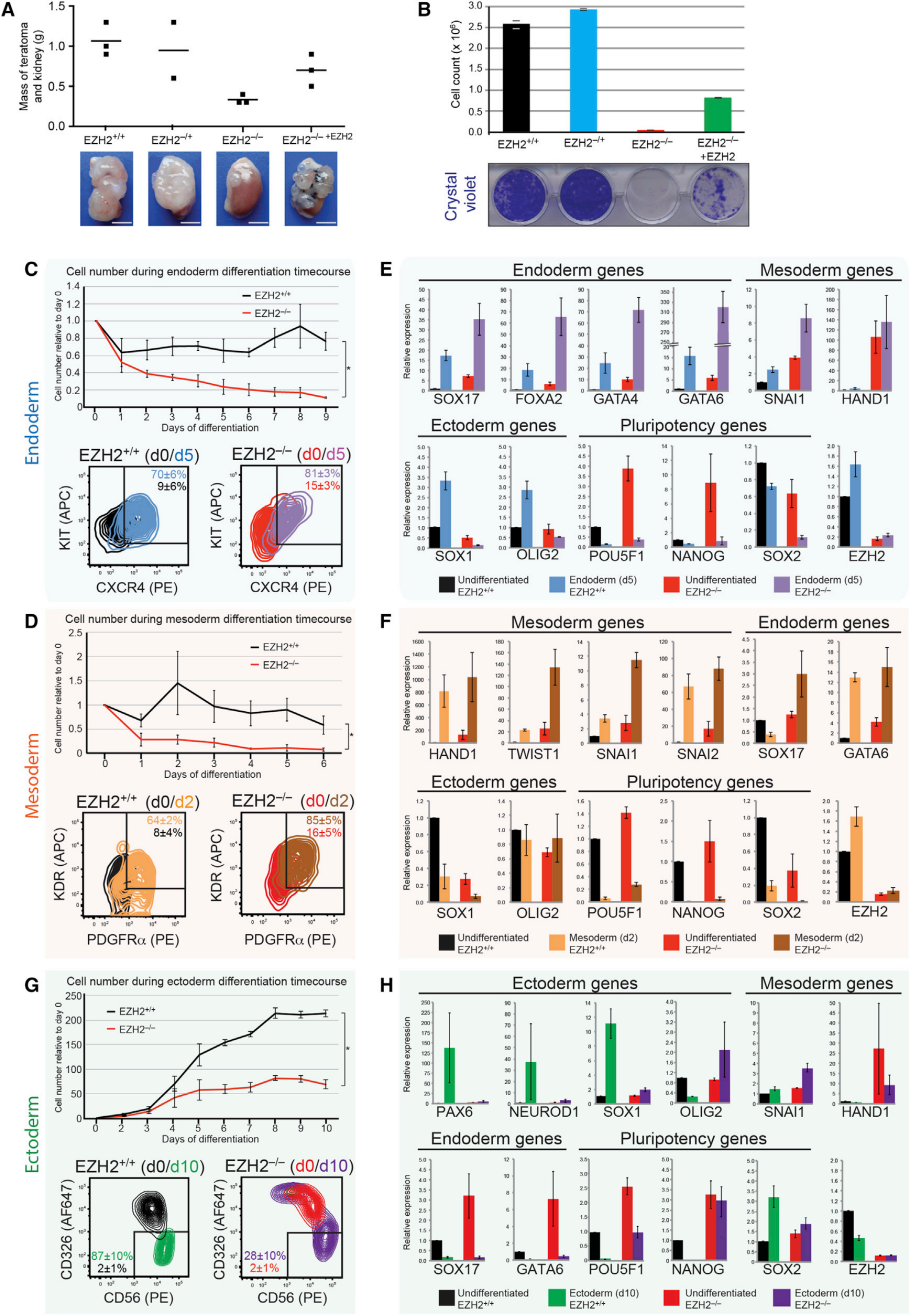
*EZH2*-Deficient hESCs Can Initiate Differentiation but Are Severely Impaired in Generating Mature Cell Types (A) *EZH2*^–/–^ ESCs fail to generate teratomas. Mass of teratoma and kidney samples for indicated ESC lines, with images shown underneath (scale bar, 5 mm). Additional images and histology analysis are provided in Figures S6A and S6B. (B) ESCs were induced to differentiate with retinoic acid for 5 days. Cell counts (upper) and crystal violet stain (lower) reveal that few *EZH2*^–/–^ ESCs remain after 5 days compared to control ESCs. Short bars indicate mean values for the two biological replicates. (C) *EZH2*-deficient ESCs can generate early endoderm cells. Upper panel shows cell counts over endoderm differentiation time course. Lower panel shows flow cytometry analysis of endoderm markers KIT/CXCR4 in undifferentiated *EZH2*^+/+^ ESCs (black), day 5 endoderm differentiated *EZH2*^+/+^ (blue), undifferentiated *EZH2*^–/–^ ESCs (red), and day 5 endoderm differentiated *EZH2*^–/–^ (purple). Inset numbers show percentage positive cells for each cell population (mean of three biological replicates, with range). (D) *EZH2*-deficient ESCs can generate early mesoderm cells. Upper panel shows cell counts over 6 days of mesoderm differentiation. Lower panel shows flow cytometry analysis of mesoderm markers KDR/PDGFRα. (E) RT-qPCR analysis of endoderm, mesoderm, ectoderm and pluripotency genes in undifferentiated (black) and day 5 endoderm differentiated (blue) *EZH2*^+/+^ ESCs, and undifferentiated (red) and day 5 endoderm differentiated (purple) *EZH2*^–/–^ ESCs. Note that *POU5F1* and *NANOG* are also associated with ESC differentiation (Loh and Lim, 2011), which may underlie their elevated expression patterns in *EZH2*^–/–^ ESCs. (F) qRT-PCR analysis of undifferentiated and day 2 mesoderm differentiated ESCs. (G) *EZH2*-deficient ESCs can generate early ectoderm cells, but with significantly reduced efficiency compared to EZH2^+/+^ ESCs. Upper panel shows cell counts over 10 days of ectoderm differentiation. Lower panel shows flow cytometry analysis of ectoderm marker CD56 and undifferentiated ESC marker CD326. Note the significantly decreased efficiency of ectoderm differentiation in *EZH2*^–/–^ ESCs compared to *EZH2*^+/+^ ESCs (p = 0.01; unpaired two-sided t test). (H) RT-qPCR analysis of undifferentiated and day 10 ectoderm differentiated ESCs. For all panels, data show mean ± SEM of three biological replicates and were compared using an unpaired two-sided t test (^∗^p < 0.05).

We next investigated the impact of *EZH2* deletion on the ability of hESCs to differentiate correctly. We injected each hESC line into the kidney capsule of three immunocompromised mice to test for teratoma formation. Control hESC lines produced teratomas consisting of mature cell types derived from all three germ lineages. By contrast, *EZH2*^–/–^ ESCs failed to produce teratomas in two mice and generated a very small mass in one mouse, which consisted of a restricted set of cell types, including immature adipocytes and epithelial cells (Figures 6A, S6A, and S6B). Although the DOX-inducible *EZH2* transgene could partially rescue the *EZH2*^–/–^ phenotype, we noticed that the teratomas formed from the *EZH2*^–/–^^+*EZH2*^ ESCs were smaller and displayed different morphology compared to the *EZH2*^+/+^ and *EZH2*^–/+^ ESC teratomas (Figures 6A, S6A, and S6B). The difference is likely to be because the cells were not provided with DOX once they were injected in situ, and therefore EZH2 levels would be lost gradually over several days. Further investigation of *EZH2* function in late-stage cell differentiation revealed that very few *EZH2*^–/–^ ESCs survived after 5 days of retinoic-acid-mediated differentiation in vitro, compared to *EZH2*^+/+^ and *EZH2*^–/+^ ESCs (Figure 6B). Restoration of *EZH2* with the DOX-inducible transgene partially rescued the defect, although we noticed that the transgene was silenced at the later stages of cell differentiation, thereby hindering a full rescue. Together, these results lead us to conclude that hESCs require *EZH2* to form late-stage differentiated cell types.

We next studied the early stages of ESC differentiation. PRC2 loss-of-function mutant embryos initiate but fail to complete gastrulation (Faust et al., 1995, O’Carroll et al., 2001, Pasini et al., 2004); however, a detailed examination of PRC2-deficient mouse or human ESC differentiation toward early developmental progenitors using defined conditions has not been reported. To investigate these developmental events, we initiated directed and separate differentiation toward endoderm, mesoderm, and ectoderm progenitors, using defined conditions and lineage-specific markers (Supplemental Experimental Procedures). We observed that cell number declined sharply during endoderm and mesoderm differentiation of *EZH2*^–/–^ ESCs, such that very few cells remained by the end of their differentiation protocols (Figures 6C and 6D). In contrast, cell number was maintained in *EZH2*^+/+^ ESCs during differentiation. We examined the cells at a mid-stage time point as this allowed us to obtain sufficient cells for analysis. Quantitative analysis of lineage-specific, cell-surface markers using flow cytometry showed that *EZH2*^–/–^ ESCs were capable of differentiating into early-stage endoderm (defined as KIT^+^/CXCR4^+^) (Nostro et al., 2011) and mesoderm (defined as PDGFRα^+^/KDR^+^) (Kattman et al., 2011) and, surprisingly, formed cell populations at these time points that were more uniform in marker expression than achieved upon differentiation of *EZH2*^+/+^ ESCs (Figures 6C and 6D). To ascertain whether a pre-existing subset of endoderm progenitors were responsible for generating endoderm cells in *EZH2*^–/–^ cultures, we used flow cytometry to separate KIT^+^/CXCR4^+^ (endoderm primed) and KIT^–^/CXCR4^–^ (not endoderm primed) *EZH2*^–/–^ populations and subjected the cells to endoderm differentiation. Flow cytometry analysis showed that KIT^–^/CXCR4^–^ were highly efficient in generating endoderm, thereby demonstrating the ability of *EZH2*-deficient ESCs to respond to appropriate differentiation cues and initiate early-stage differentiation (Figure S6C).

Upon cell differentiation, pluripotency factors *POU5F1*, *NANOG*, and *SOX2* were downregulated to a similar extent in *EZH2*^–/–^ ESCs compared to *EZH2*^+/+^ ESCs (Figures 6E and 6F). This finding is in contrast to PRC2-deficient mouse ESCs, which exhibit a defect in silencing pluripotency networks during cell differentiation (Pasini et al., 2007, Shen et al., 2008). Consistent with our RNA-seq data, genes associated with endoderm and mesoderm differentiation were detected at higher levels at day 0 in *EZH2*^–/–^ ESCs compared to *EZH2*^+/+^ ESCs, and the transcript level of these genes increased further during differentiation, confirming their ability to undergo early-stage differentiation (Figures 6E and 6F). Ectopic expression of lineage-restricted genes occurred during *EZH2*^–/–^ ESC differentiation, suggesting a failure to repress alternate transcriptional programs (Figures 6E and 6F).

*EZH2*-deficient cells were also able to generate early ectoderm cells (defined as CD56^+^/CD326^–^) (Gifford et al., 2013); however, cell number during differentiation and the efficiency of differentiation were significantly reduced compared to *EZH2*^+/+^ ESCs (Figure 6G). Mis-regulation of lineage-restricted genes was observed upon ectoderm differentiation, thereby identifying a requirement for EZH2 in regulating appropriate gene expression in the early stages of ectoderm specification (Figure 6H).

Taken together, these results demonstrate that EZH2 is not required for the initial phase of hESC differentiation but is required for the robust generation of mature cell types that are produced in the later stages of differentiation in vitro or in teratoma assays.

## Discussion

**Figure 7:**
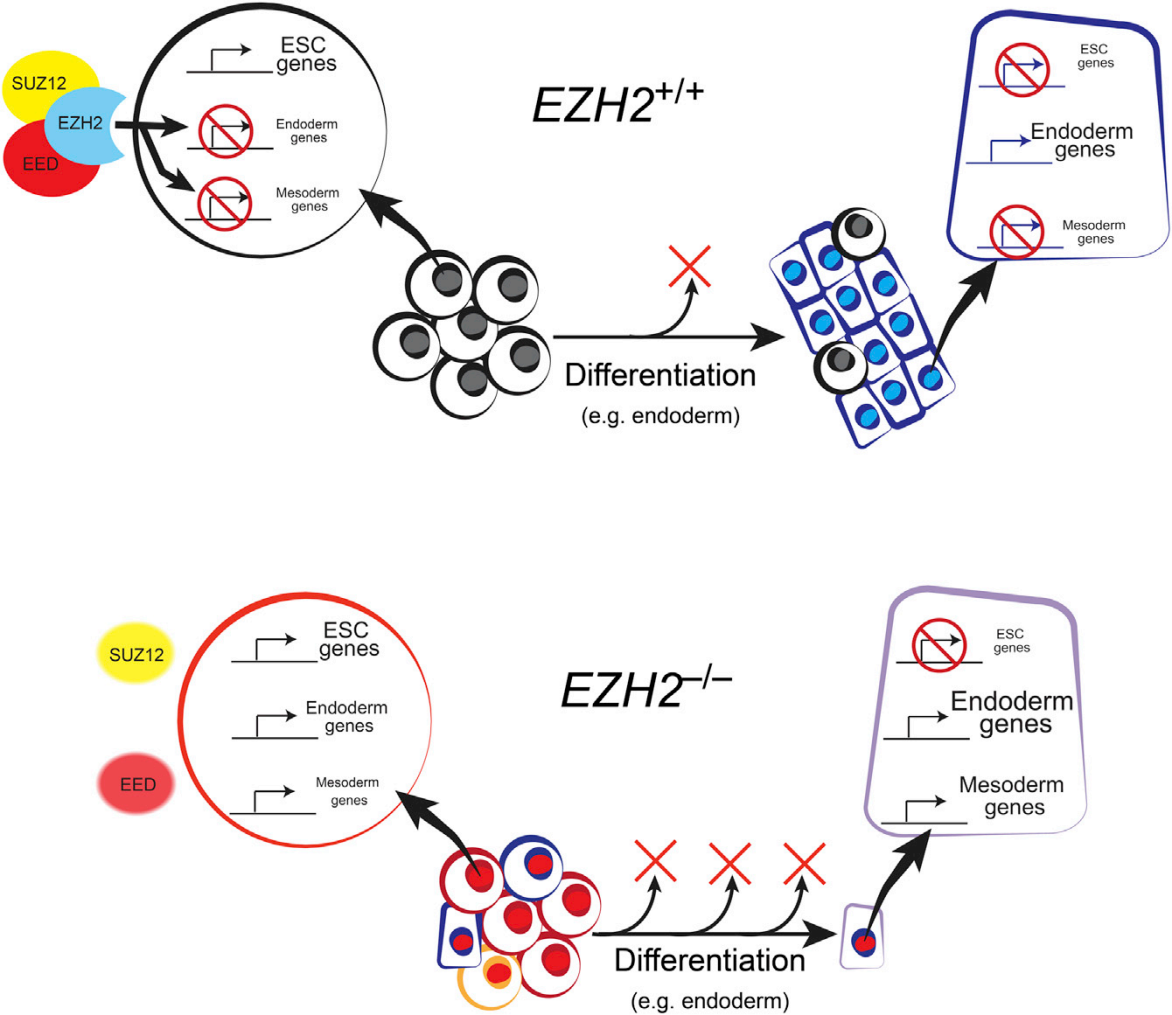
Proposed Model Summarizing the Role of *EZH2* in Regulating Transcriptional Programs and Cell Differentiation in hESCs *EZH2*^+/+^, above; and *EZH2*^–/–^, below. *EZH2* deficiency leads to loss of PRC2, transcriptional derepression of developmental regulators and self-renewal defects in hESCs. Substantial cell loss (red crosses) and gene mis-regulation is observed upon differentiation of *EZH2*^–/–^ ESCs (e.g., to endoderm in example shown).

PcG-proteins are essential regulators of cell-fate decisions and transcriptional programs during the development of several species, including *Drosophila* and the mouse. Here, we show that this important function is also required in humans during the establishment of early developmental cell types that arise upon ESC differentiation (Figure 7). Furthermore, loss of EZH2 function in undifferentiated hESCs led to the transcriptional derepression of ∼900 genes including many important developmental regulators, thereby positioning EZH2 as a key factor in controlling the transcriptome of human cell types during early development. Interestingly, not all PRC2-target genes were mis-regulated, suggesting that redundant modes of transcriptional repression are in place, or that additional cues (such as transcription factor binding) are required to fully activate those genes. In addition to developmental factors, we also detected an increased expression of cell-cycle regulators in the absence of *EZH2*, including *CDKN2A* (encoding p16INK4A and p14ARF) and *CDKN2B* (encoding p15INK4B). Given the close association between cell-cycle control and cell differentiation in hESCs (Pauklin and Vallier, 2013, Gonzales et al., 2015, Pauklin et al., 2016), it is likely that both processes contribute to the phenotype of *EZH2*-deficient cells. For example, an upregulation of p16INK4A would inhibit CDK4/6, which, in turn, would lead to an increase in Activin/Nodal activity (Pauklin and Vallier, 2013). This signaling change would promote the transcriptional programs of endoderm and mesoderm lineages and suppress ectoderm differentiation. We speculate, therefore, that the increased expression of endoderm and mesoderm genes in *EZH2*-deficient hESCs is caused jointly by the removal of repressive H3K27me3 marks, by altered signaling activities that are mediated by cell-cycle machinery, and by the cell-culture environment. Notably, our transcriptional results are consistent with a recent study that reported derepression of a subset of PRC2-target genes in *Ezh2*-deficient mouse epiblast tissue, thereby underscoring the relevance of our observations to pluripotent cells in vivo (Zylicz et al., 2015). Importantly, our analysis of individual cells further revealed that mis-regulation of genes tended to occur in a coordinated manner within lineage-restricted transcriptional programs, rather than a haphazard derepression of all PRC2-target genes as might be predicted from global cell population analysis. These results suggest the presence of feedback mechanisms that are able to promote or repress alternative cell fates during the early phases of differentiation. An exciting set of future studies will be to model and investigate the mechanisms responsible for this feedback. Our findings also raise broader questions about how cells are committed to a particular lineage during differentiation. Purifying live hESC populations that are in different transcriptional states and challenging the cells to functional assays should begin to unravel the complexities of cell-fate commitment during human development.

Despite extensive conservation in their functions, differences exist between mouse and human ESCs that lack *EZH2*; self-renewal, morphology, and proliferation are seemingly perturbed to a greater extent in human *EZH2*^–/–^ ESCs compared to mouse *Ezh2*^–/–^ ESCs (Shen et al., 2008). One potential explanation is that differences in PRC2 protein stability or function could contribute to the distinct mouse and human ESC phenotypes. For example, Eed and Suz12 levels are unaffected by the loss of Ezh2 in mouse ESCs, potentially due to a partial compensation by Ezh1 (Shen et al., 2008). In contrast, we show here that depletion of EZH2 in hESC results in loss of EED and SUZ12, despite the presence of EZH1. Interestingly, Ezh1 cannot compensate for the absence of Ezh2 during mouse ESC differentiation or embryo gastrulation (O’Carroll et al., 2001, Shen et al., 2008). We speculate there is a context-dependent role for Ezh1 and that the compensatory function diminishes as cells enter the post-implantation phase of development, which could partially explain the apparent inability of EZH1 to fulfill a compensatory role in *EZH2*-deficient hESCs. In addition, Ezh1 is able to repress gene transcription through methylation-independent mechanisms in somatic cells, potentially via chromatin compaction (Margueron et al., 2008). It will therefore be interesting in future studies to more precisely define the functional interplay between EZH1 and EZH2 in early human developmental cell types.

A second potential explanation for the distinct phenotypes is that mouse and human ESCs are known to represent different pluripotent states, with hESCs considered to be primed for differentiation (Nichols and Smith, 2009, Rossant, 2015). The *EZH2*-deficient phenotype may therefore manifest differently depending on cell state, a concept recently proposed for *DNMT1*-depleted ESCs (Liao et al., 2015). It will be important in future studies to test this hypothesis by investigating the role of PRC2 in human “naive” pluripotent cells, which are reported to be more similar to mouse ESCs (Manor et al., 2015). Furthermore, it is interesting to consider that PRC2 may contribute to the balance required for primed-state pluripotency by enabling low-level expression of lineage-specifying developmental regulators while constraining their levels so that they do not overwhelm the maintenance of the undifferentiated state. Given that the ectopic expression of several EZH2 target genes, such as *SOX17* and *GATA6*, can induce the differentiation of hESC (Séguin et al., 2008, Wamaitha et al., 2015), it is plausible that derepression of these and other developmental regulators in the absence of *EZH2* results in a shift toward an increased level of spontaneous differentiation that is observed in *EZH2*-deficient hESCs. Thus, our analysis of PcG function in hESCs should lead to a better understanding of the processes that regulate lineage priming and cell-fate commitment and inform similar events that occur in other species and cell types.

Genome-wide mapping has revealed that dynamic changes in epigenetic marks, including H3K27me3 localization, occur upon hESC differentiation (Gifford et al., 2013, Xie et al., 2013). A prevailing model proposes that this epigenetic reconfiguration is required to coordinate transcriptional programs and provide a memory of cell identity. We have now tested this model, and we show that EZH2 is not required for the initial phase of hESC differentiation as ectoderm, mesoderm, and endoderm germ lineages can form in the absence of EZH2; however, the mutant cells mis-express lineage-specific genes are unstable and are gradually lost over the differentiation time course. Interestingly, and in contrast to PRC2-deficient mouse ESCs (Pasini et al., 2007, Shen et al., 2008), pluripotency genes were downregulated appropriately upon differentiation of *EZH2*-deficient hESCs, suggesting that these genes are silenced by PRC2-independent pathways. The observed differentiation defects and reduction in cell survival are therefore unlikely to be caused by aberrant expression of pluripotency factors, but rather by mis-expression of lineage-specifying genes and cell-cycle regulators. Finally, although the *EZH2*-deficient hESCs were unable to form mature cell types, the rescue of early differentiation defects by conditionally restoring *EZH2* levels should enable the role of PRC2 to be investigated during late-stage in vitro differentiation. As the *EZH2*^–/–^^+*EZH2*^ cells could not fully recapitulate the parental wild-type cells in the teratoma and the RA-differentiation experiments, alternative conditional systems might be better suited for the investigation of *EZH2* in late-stage cell differentiation. Interestingly, the results from our teratoma experiments suggest that once the *EZH2*^–/–^ ESCs have overcome the initial early-stage differentiation barrier (enabled by residual EZH2), they are able to specialize along certain tissue lineages. The differences in morphology and tissue composition of the teratomas are presumably, to some extent, a reflection of what differentiation pathways are accessible to *EZH2*-deficient cells. Future studies using alternative conditional strategies and more precise differentiation systems should lead to a better understanding of epigenetic modifiers in the generation of specialized cell types. Artificially controlling *EZH2* levels may also have useful practical applications in producing desired cell types, as has been demonstrated recently to boost production of beta cell progenitors (Xu et al., 2014).

Taken together, our study provides a comprehensive examination of *EZH2* function in hESC pluripotency and differentiation. Of note is that PRC2 mediates the self-renewal and differentiation of adult stem cells and cancer stem cells (Sauvageau and Sauvageau, 2010). Our findings therefore not only reveal the role of epigenetic modifiers and associated histone marks in regulating the genome during early human development, but also establish general principles that can be applicable to stem cells involved in homeostasis and disease.

## Experimental Procedures

### Cell Culture

hESCs (H9/WA09, obtained from WiCell; WIBR3, kindly provided by Rudolph Jaenisch; FiPS, kindly provided by Austin Smith) were cultured at 37°C in 5% CO_2_ in air on CF1 irradiated mouse embryonic fibroblasts (MEFs) in Advanced DMEM containing 20% knockout serum replacement supplemented with 2 mM L-glutamine, 0.1 mM β-mercaptoethanol, 1 × penicillin/streptomycin, 1 × non-essential amino acids (all from Thermo Fisher Scientific) and 4 ng/ml FGF2 (WT-MRC Cambridge Stem Cell Institute). Where indicated, DOX was added at 1 μg/ml. For feeder-free culture, ESCs were transferred onto Vitronectin matrix in TeSR-E8 media (STEMCELL Technologies). Authentication of the hESCs was achieved by confirmation of expression of pluripotency gene and protein markers. Cells were routinely verified as mycoplasma-free using a PCR-based assay. Teratoma formation assays were performed in a designated facility under licenses granted by the UK Home Office. Additional cell-culture materials and methods are detailed in Supplemental Experimental Procedures.

### Targeted Deletion of *EZH2*

EZH2 gRNA (CCGCTTCTGCTGTGCCCTTATC) was designed using http://crispr.mit.edu (Hsu et al., 2013). The gRNA sequence was incorporated into the U6 target gRNA expression vector (Mali et al., 2013) and synthesized as a gBlock by Integrated DNA Technologies. The *EZH2* gRNA gBlock was sub-cloned into pCR2.1-TOPO (Thermo Fisher Scientific) and verified by sequencing. hESCs were dissociated into single cells using Accutase (Thermo Fisher Scientific). H9 ESCs (2 million) were nucleofected with 5 μg pCas9_GFP (Addgene plasmid # 44719) and 5 μg *EZH2* gRNA expression vector. After 48 hr, 10,000 GFP-positive single cells were isolated by FACS and seeded onto MEF in a 10-cm tissue culture dish in ESC media supplemented with 10 μM Rho Kinase inhibitor (Cell Guidance Systems) for the first 24 hr. Individual clones were picked and expanded in 24-well plates. Mutations were validated by DNA sequencing of TOPO cloned PCR products. As a check for specificity, ten predicted off-target gRNA sites within genes were tested and verified to contain unmodified sequences.

### Plasmid Constructs

To construct PB-TET-EZH2-ires-mCherry plasmid, the *EZH2* coding sequence was amplified using primers EZH2_attb_F and EZH2_attb_R and sub-cloned into PB-TET-ires-mCherry plasmid. To generate *EZH2*^–/– +*EZH2*^ ESCs, *EZH2*^–/+^ ESCs were lipofected with 1 μg PB-TET-EZH2-ires-mCherry, 1 μg pCAG-rtTA-Puro, and 2 μg pCyL43 (Wang et al., 2008) followed by selection with 1 μg/ml Puromycin.

To remove PB-TET-EZH2-ires-mCherry, DOX-induced *EZH2*^–/– +*EZH2*^ ESC were nucleofected with 5 μg pCMV-hyPBase (Yusa et al., 2011) and 1 μg Turbo-GFP (Lonza). After 48 hr, 10,000 GFP/mCherry double-positive single cells were isolated by FACS and seeded onto MEFs in a 10-cm tissue culture dish in ESC media supplemented with 10 μM Rho Kinase inhibitor for the first 24 hr. Individual clones were picked and expanded in 24-well plates. DNA was genotyped using mCherry_Geno and TET-Prom_Geno primers to confirm removal of PB-TET-EZH2-ires-mCherry.

### Statistics

For Figure 3D, the data are significantly departed from normality (p < 0.05; D’Agostino-Pearson omnibus normality test) and the variance is different between the groups (p < 0.05; Brown-Forsythe test); therefore, a non-parametric test was used. For statistical analysis of data within Figures 5 and 6, the scatter of the data lead us to assume that the samples comes from a normally distributed population and that the variability between the groups is about the same; therefore, parametric tests were used.

## Author Contributions

A.C. and P.J.R.-G. designed the study, interpreted the results, and wrote the manuscript. A.C. generated all cell lines and performed all experiments. A.J.C. carried out the ectoderm differentiation experiments. N.P.M. generated several ChIP-seq libraries. A.R.S. performed cell-line characterization. T.C. assisted with generating the single-cell RNA-seq libraries. S.A. analyzed single-cell RNA-seq data. P.J.R.-G. conceived and supervised the project, performed experiments, and analyzed data. We consider A.J.C. and N.P.M. to have contributed equally.

## Accession Numbers

The accession number for the sequencing data reported in this paper is GEO: GSE76626.

## Supplemental Information

Document S1. Supplemental Experimental Procedures and Figures S1–S6

Document S2. Article plus Supplemental Information
