# Supplementary material for: Deletion of the Polycomb-Group Protein EZH2 Leads to Compromised Self-Renewal and Differentiation Defects in Human Embryonic Stem Cells
Source: Cell Rep. 2016 Dec 6;17(10):2700–14. doi: 10.1016/j.celrep.2016.11.032 (PMC5177603; doi:10.1016/j.celrep.2016.11.032)
Supplement: Document S1. Supplemental Experimental Procedures and Figures S1–S6 [file mmc1.pdf]

Cell Reports, Volume *17*

Supplemental Information

Deletion of the Polycomb-Group Protein EZH2 Leads to Compromised Self-Renewal and Differentiation Defects in Human Embryonic Stem Cells

Adam Collinson, Amanda J. Collier, Natasha P. Morgan, Arnold R. Sienerth, Tamir Chandra, Simon Andrews, and Peter J. Rugg-Gunn

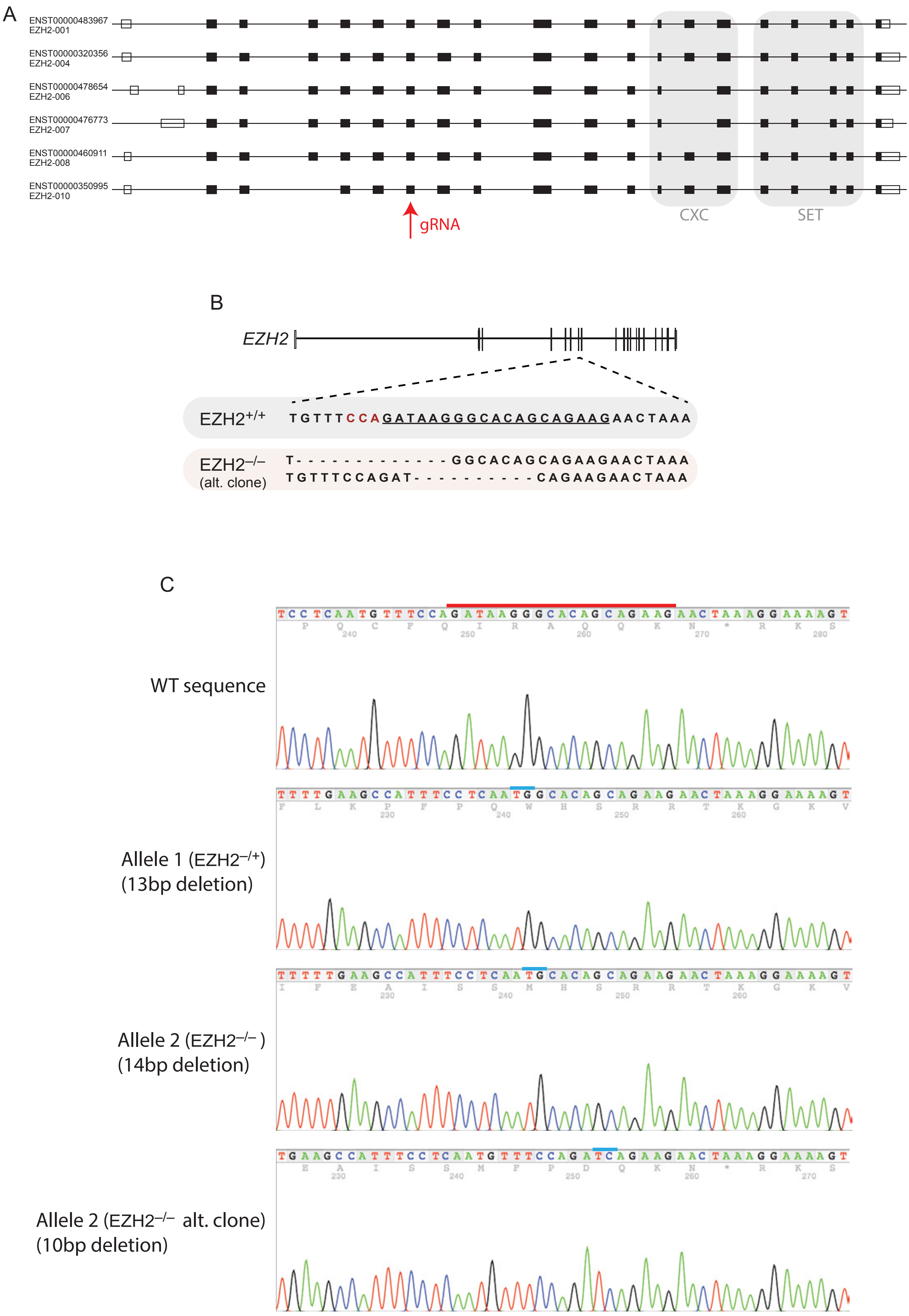
A
ENST00000483967
EZH2-001
ENST00000320356
EZH2-004
ENST00000478654
EZH2-006
ENST00000476773
EZH2-007
ENST00000460911
EZH2-008
ENST00000350995
EZH2-010
gRNA
CXC
SET
B
EZH2
EZH2+/+ TGTTTCCAGATAAGGGCACAGCAGAAGAACTAAA
EZH2–/– (alt. clone) T- - - - - - - - - - - - - GGCACAGCAGAAGAACTAAA
TGTTTCCAGAT- - - - - - - - - - CAGAAGAACTAAA
C
WT sequence
Allele 1 (EZH2–/+) (13bp deletion)
Allele 2 (EZH2–/–) (14bp deletion)
Allele 2 (EZH2–/– alt. clone) (10bp deletion)

Figure S1, related to Figure 1.

Targeted deletion of *EZH2* in human ESC.

(A) Exon structure of *EZH2* isoforms (Ensembl) and the location of gRNA sequence.

(B) DNA sequence of the deletions in an alternative $EZH2^{-/-}$ ESC line is shown for both alleles. Mutation causes frameshift and premature stop codon. The gRNA sequence is underlined and protospacer adjacent motif highlighted in red.

(C) Sanger DNA sequencing chromatograms for $EZH2^{+/+}$, $EZH2^{-/+}$ and two $EZH2^{-/-}$ ESC lines. The red line indicates the position of the gRNA sequence. The blue lines indicate the position of the deletion.

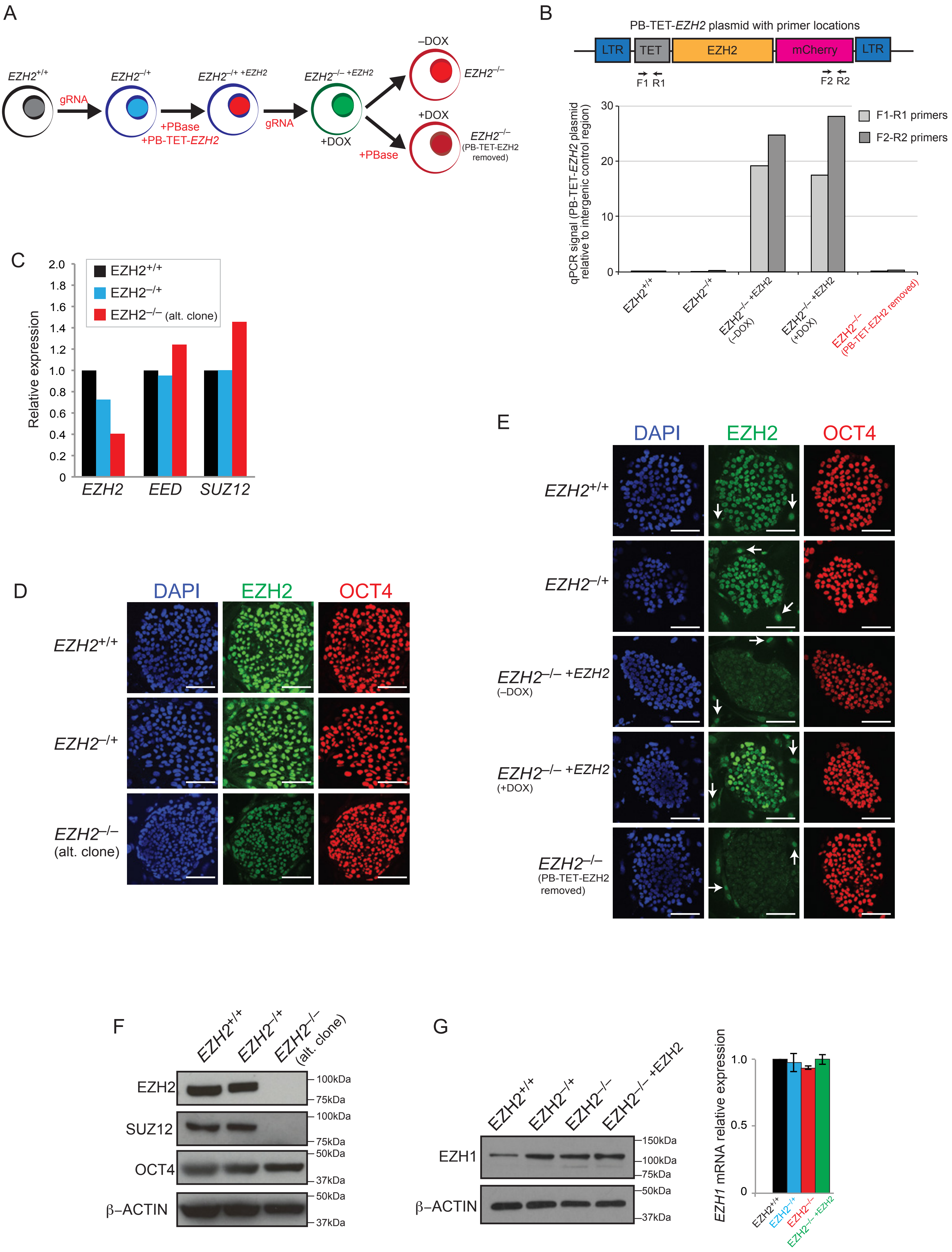
A
EZH2+/+
gRNA
EZH2−/+
+PBase
+PB-TET-EZH2
EZH2−/+ +EZH2
gRNA
EZH2−/− +EZH2
+DOX
−DOX
EZH2−/−
+DOX
+PBase
EZH2−/−
(PB-TET-EZH2 removed)
B
PB-TET-EZH2 plasmid with primer locations
LTR
TET
EZH2
mCherry
LTR
F1 R1
F2 R2
qPCR signal (PB-TET-EZH2 plasmid relative to intergenic control region)
F1-R1 primers
F2-R2 primers
EZH2+/+
EZH2−/+
EZH2−/− +EZH2 (−DOX)
EZH2−/− +EZH2 (+DOX)
EZH2−/− (PB-TET-EZH2 removed)
C
Relative expression
EZH2+/+
EZH2−/+
EZH2−/− (alt. clone)
EZH2
EED
SUZ12
D
DAPI
EZH2
OCT4
EZH2+/+
EZH2−/+
EZH2−/− (alt. clone)
E
DAPI
EZH2
OCT4
EZH2+/+
EZH2−/+
EZH2−/− +EZH2 (−DOX)
EZH2−/− +EZH2 (+DOX)
EZH2−/− (PB-TET-EZH2 removed)
F
EZH2+/+
EZH2−/+
EZH2−/− (alt. clone)
EZH2
SUZ12
OCT4
β−ACTIN
100kDa
75kDa
50kDa
37kDa
G
EZH2+/+
EZH2−/+
EZH2−/−
EZH2−/− +EZH2
EZH1
β−ACTIN
150kDa
100kDa
75kDa
50kDa
37kDa
EZH1 mRNA relative expression

Figure S2, related to Figure 1.

Additional characterisation of *EZH2*-deficient human ESC.

(A) Overview of targeting strategy including generation of *EZH2*-deficient ESC containing a DOX-inducible *EZH2* transgene (PB-TET-*EZH2*) and after transient expression of piggyBac transposase (PBase) to excise all copies of the transgene.

(B) qPCR on genomic DNA extracted from indicated ESC lines using two primer pairs that are specific for the PB-TET-*EZH2* transgene are shown relative to one primer pair that amplifies a control region that is not within the transgene. These data demonstrate that transient PBase expression excised the transgene.

(C) RT-qPCR analysis of PRC2 components *EZH2*, *EED* and *SUZ12* in $EZH2^{+/+}$, $EZH2^{-/+}$ and an alternative $EZH2^{-/-}$ ESC line.

(D) Immunofluorescent microscopy confirms a strong reduction in EZH2 levels in an alternative $EZH2^{-/-}$ ESC line compared to control ESC. The antibody was raised against a C-terminal epitope of EZH2. OCT4 expression indicates undifferentiated cells within an ESC colony. Scale bars, 100μm.

(E) Immunofluorescent microscopy reveals a strong reduction in EZH2 levels in $EZH2^{-/-}$ ESC in the absence of DOX and also after transgene excision (in the presence of DOX). The antibody was raised against an N-terminal epitope of EZH2. OCT4 expression indicates undifferentiated cells within an ESC colony. Arrows point to MEF. Scale bars, 100μm.

(F) EZH2 and SUZ12 are undetectable in an alternative $EZH2^{-/-}$ ESC line by Western blot analysis. β-ACTIN is loading control. Mass in kDa.

(G) EZH1 is expressed in $EZH2^{-/-}$ ESC by Western blot analysis (left panel) and by qRT-PCR analysis (right panel), and levels are unaffected by the loss of *EZH2*. β-ACTIN is loading control. Mass in kDa. qRT-PCR data show mean ± s.d.; n=3 biological replicates.

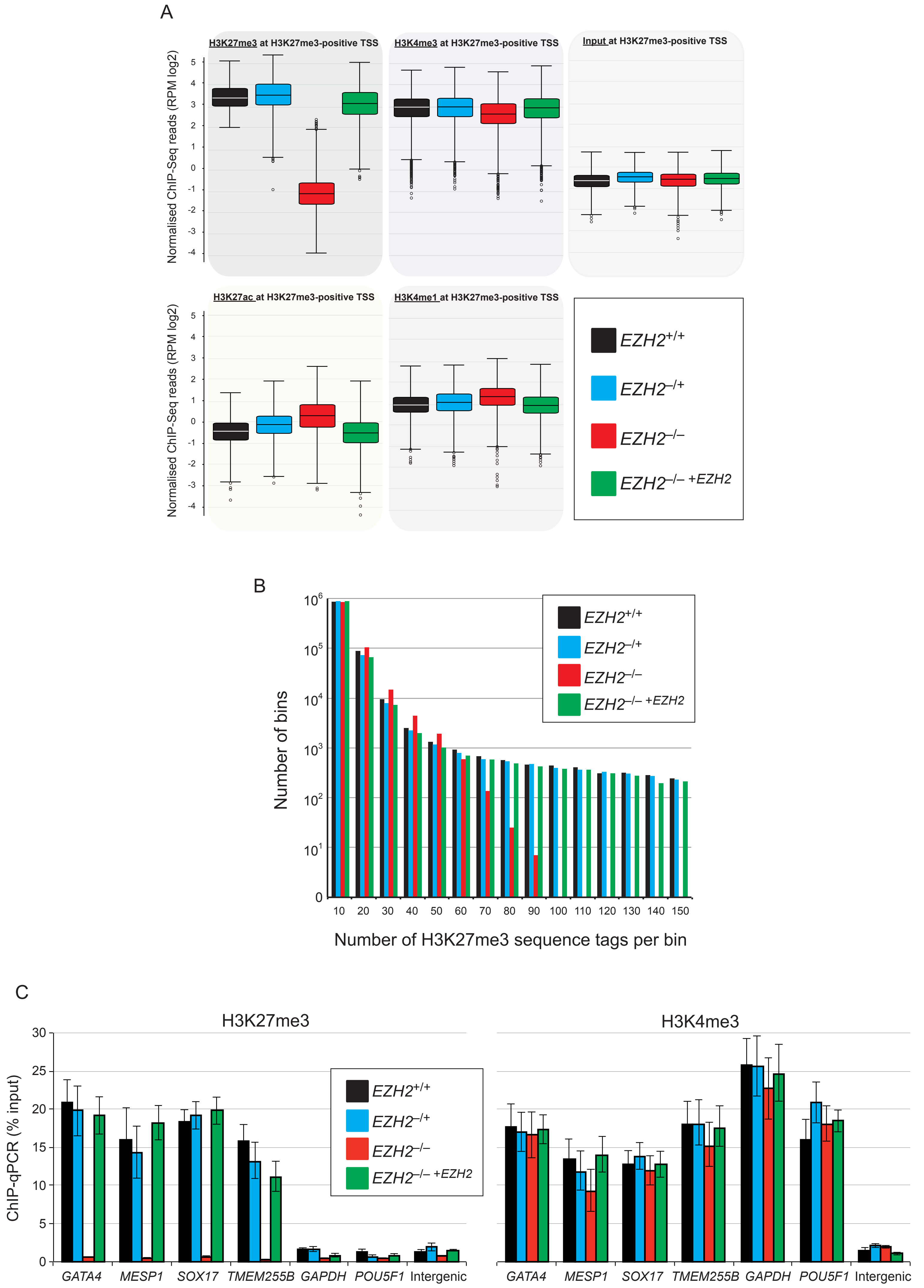
A
H3K27me3 at H3K27me3-positive TSS
H3K4me3 at H3K27me3-positive TSS
Input at H3K27me3-positive TSS
H3K27ac at H3K27me3-positive TSS
H3K4me1 at H3K27me3-positive TSS
Normalised ChIP-Seq reads (RPM log2)
EZH2+/+
EZH2–/+
EZH2–/–
EZH2–/– +EZH2
B
Number of bins
Number of H3K27me3 sequence tags per bin
C
H3K27me3
H3K4me3
ChIP-qPCR (% input)
GATA4
MESP1
SOX17
TMEM255B
GAPDH
POU5F1
Intergenic

Figure S3, related to Figure 2.

Disruption of *EZH2* causes loss of promoter and genome-wide H3K27me3 in human ESC.

(A) Boxplot analysis of normalised ChIP-Seq reads for H3K27me3-positive (in EZH2$^{+/+}$ ESC) gene promoters (±2.5kb TSS; n=2081) in the indicated ESC lines. Panels show four different histone modifications, plus input sample. Promoters show a substantial reduction of H3K27me3 and moderate increase in H3K27ac in *EZH2*$^{-/-}$ ESC compared to control ESC.

(B) Genome-wide reduction in H3K27me3 levels in *EZH2*-deficient ESC. 150Mb of Chromosome 5 (arbitrarily chosen) was divided into 300bp bins with 150bp step size, and the number of non-duplicated reads per bin was quantified and globally normalised to the data set with the highest coverage. A similar number of bins have a low sequence count per bin in all four ESC lines; however EZH2$^{-/-}$ ESC have very few bins with a high sequence count per bin.

(C) Additional validation provided by qPCR analysis of ChIP DNA for several gene promoter regions in *EZH2*-deficient and control ESC lines. Gene promoters include *GATA4*, *MESP1*, *SOX17* and *TMEM255B*, which are marked by H327me3 and H3K4me3 in wild-type ESC; *GAPDH* and *POU5F1*, which are marked by H3K4me3 only in ESC; and a negative control intergenic region that is not marked by either histone modification. Consistent with the ChIP-Seq data, the qPCR analysis reveals that H3K27me3 signals are reduced to background levels in *EZH2*$^{-/-}$ ESC, compared to control ESC, whereas H3K4me3 signals are largely unaffected. Data show mean ± s.d.; n=3 biological replicates.

Collinson_Fig.S4

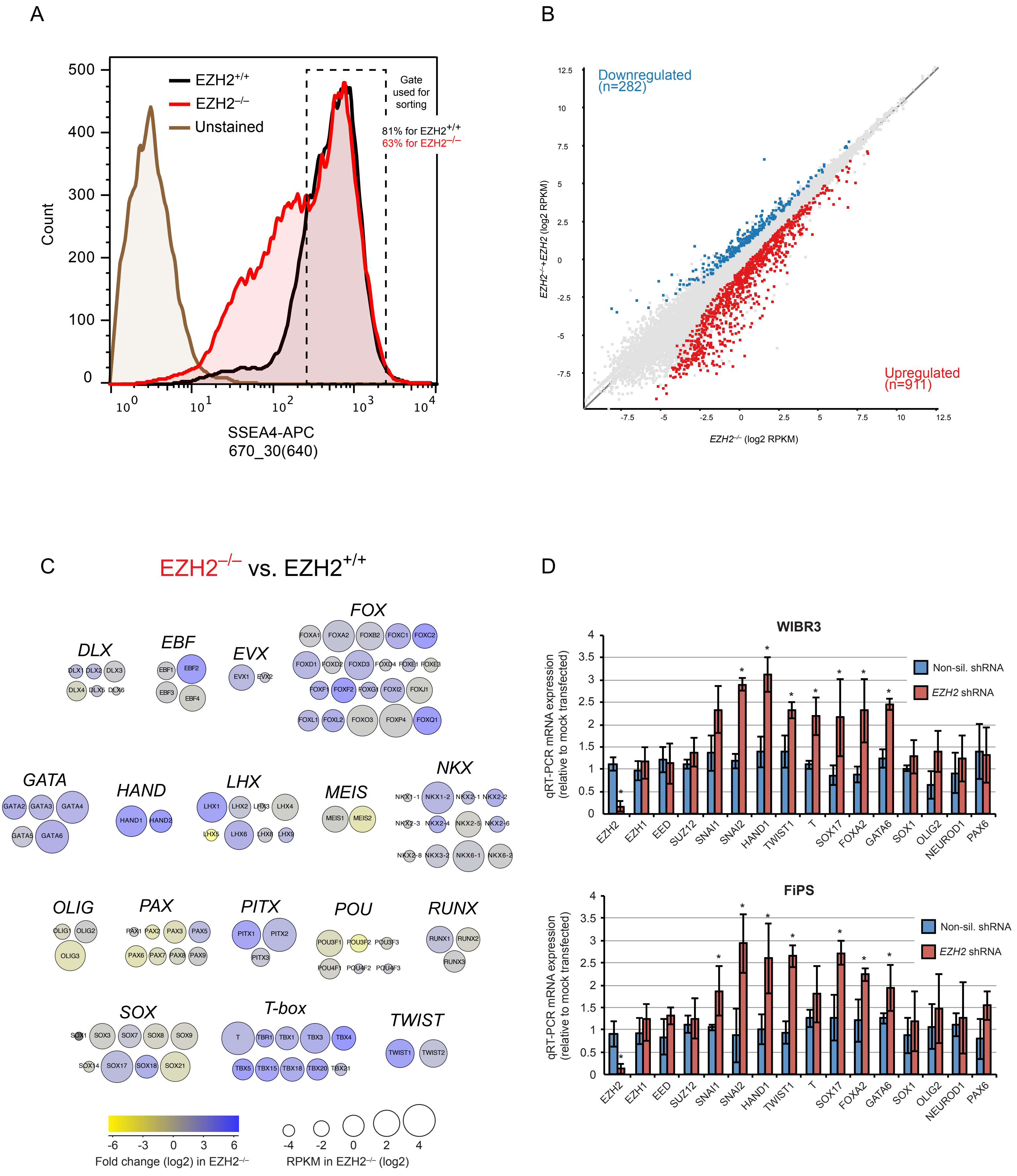

Figure S4, related to Figure 3.

Genes encoding key developmental regulators are transcriptionally derepressed in *EZH2*-deficient hESC.

(A) Flow cytometry histogram of SSEA4 levels and sorting strategy used for $EZH2^{+/+}$ and $EZH2^{-/-}$ ESC. Numbers show percentage positive cells within sorting gate for each cell line.

(B) Scatter plot of RNA-Seq transcript levels of all genes in $EZH2^{-/-}$ ESC versus $EZH2^{-/- +EZH2}$ ESC. Upregulated and downregulated genes are highlighted.

(C) A subset of direct EZH2 target genes is grouped into families. The colour of each circle represents the log2 fold change in $EZH2^{-/-}$ ESC relative to $EZH2^{+/+}$ ESC. The size of each circle represents the expression value of the gene in $EZH2^{-/-}$ ESC.

(D) qRT-PCR analysis of WIBR3 (upper) and FiPS (lower) human pluripotent stem cells lines 72h after transfection with shRNA targeting *EZH2*. Non-silencing shRNA provides a negative control. All values are displayed relative to mock-transfected cells. *EZH2* mRNA was depleted to ~15% levels compared to mock-transfected and non-silencing samples. *EZH1*, *EED* and *SUZ12* mRNA levels are unaffected. Several genes encoding developmental regulators are transcriptionally derepressed upon *EZH2* knockdown, particularly those associated with mesoderm (*SNAI1*, *SNAI2*, *HAND1*, *TWIST1*, T) and endoderm (*SOX17*, *FOXA2*, *GATA6*) differentiation. Genes associated with ectoderm (*SOX1*, *OLIG2*, *NEUROD1*, *PAX6*) differentiation are unchanged. Data show mean ± s.d.; n=3 biological replicates, and the *EZH2* shRNA samples were compared against the non-silencing shRNA samples using an unpaired two-sided t-test (*, $p<0.05$).

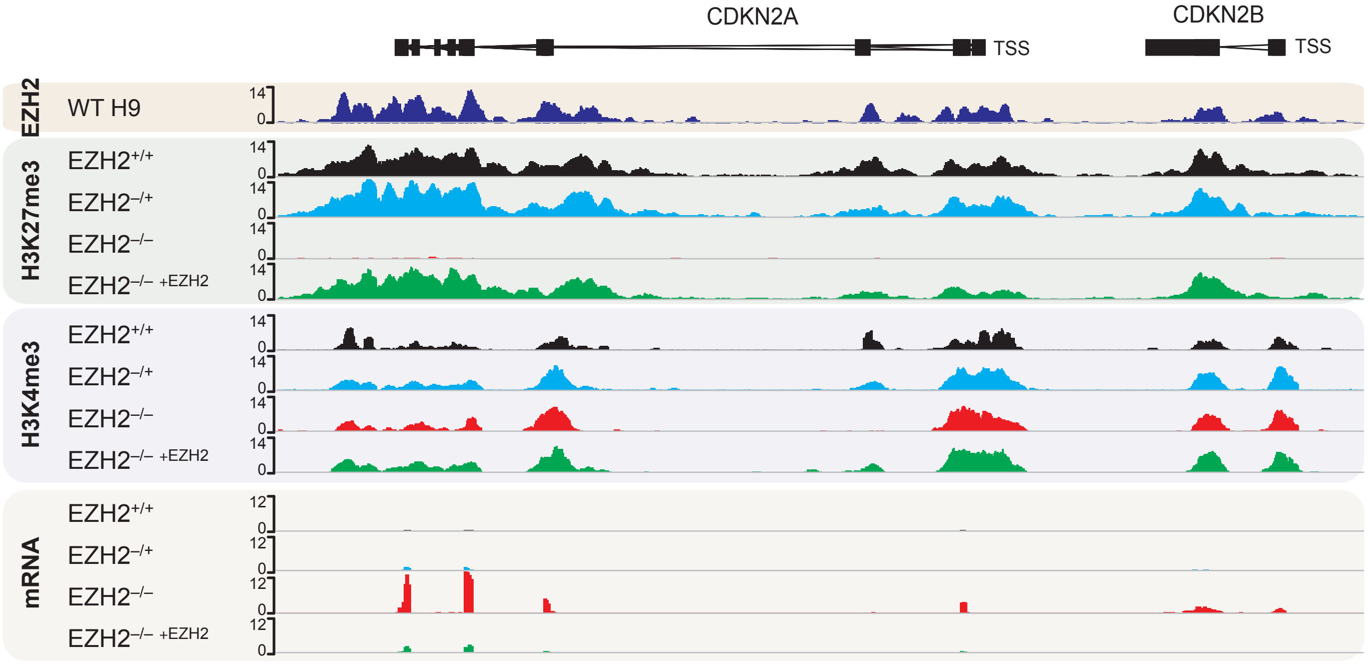

CDKN2A
TSS
CDKN2B
TSS
EZH2
WT H9
14
0
H3K27me3
EZH2+/+
EZH2–/+
EZH2–/–
EZH2–/– +EZH2
H3K4me3
EZH2+/+
EZH2–/+
EZH2–/–
EZH2–/– +EZH2
mRNA
EZH2+/+
EZH2–/+
EZH2–/–
EZH2–/– +EZH2
12
0

Figure S5, related to Figure 5.

Genes encoding key cell cycle regulators are transcriptionally derepressed in *EZH2*-deficient human ESC.

ChIP-Seq and mRNA-Seq tracks of two genes encoding key cell cycle regulators illustrate the association between loss of H3K27me3 and transcriptional upregulation in $EZH2^{-/-}$ ESC compared to control ESC. The transcriptional upregulation of *CDKN2A* and *CDKN2B* could partially underlie the proliferation impairment in $EZH2^{-/-}$ ESC.

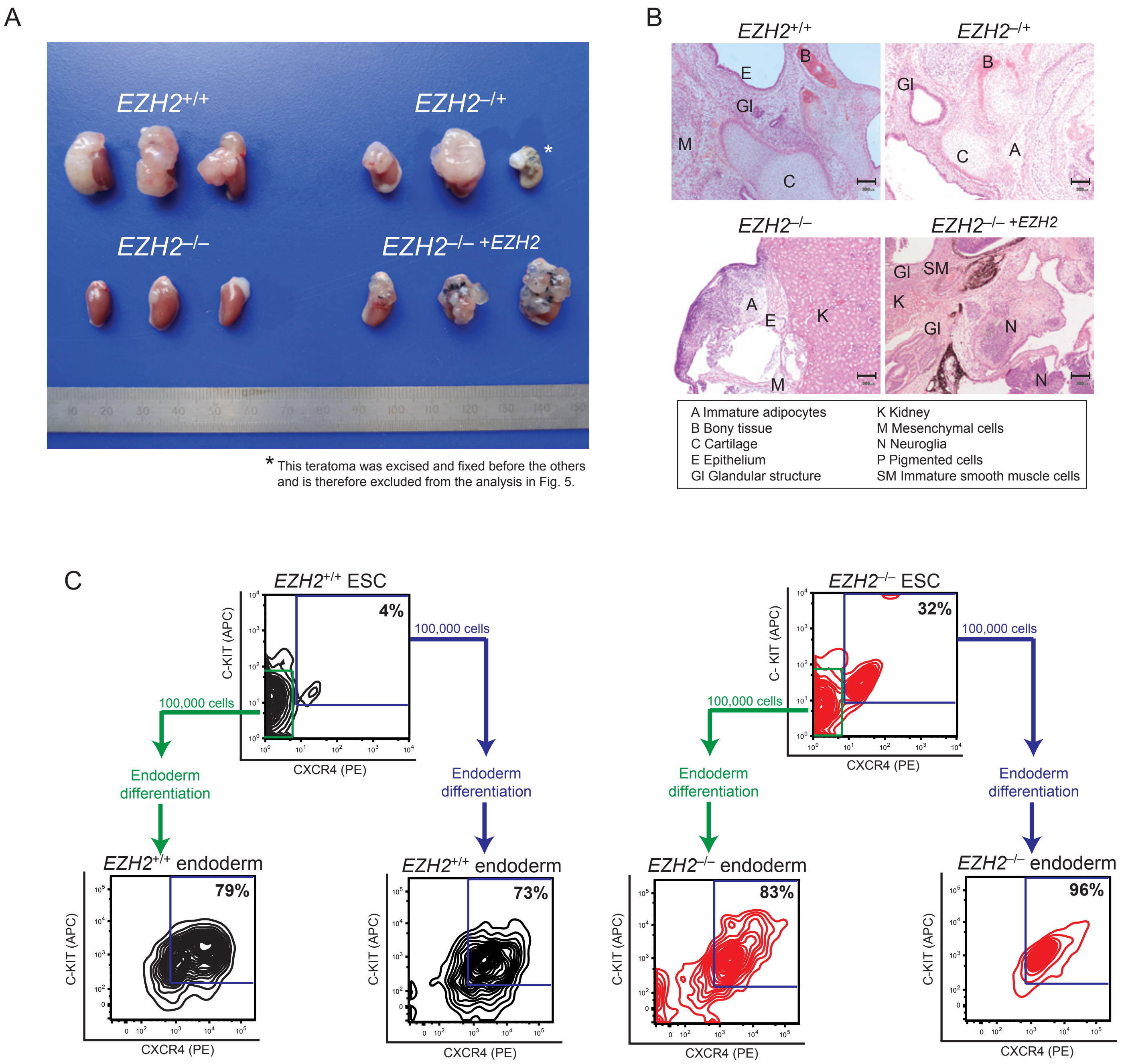
A
EZH2+/+
EZH2–/+
EZH2–/–
EZH2–/– +EZH2
*
* This teratoma was excised and fixed before the others and is therefore excluded from the analysis in Fig. 5.
B
EZH2+/+
EZH2–/+
EZH2–/–
EZH2–/– +EZH2
A Immature adipocytes
B Bony tissue
C Cartilage
E Epithelium
Gl Glandular structure
K Kidney
M Mesenchymal cells
N Neuroglia
P Pigmented cells
SM Immature smooth muscle cells
C
EZH2+/+ ESC
4%
C-KIT (APC)
CXCR4 (PE)
100,000 cells
100,000 cells
Endoderm differentiation
Endoderm differentiation
EZH2+/+ endoderm
79%
EZH2+/+ endoderm
73%
EZH2–/– ESC
32%
EZH2–/– endoderm
83%
EZH2–/– endoderm
96%

Figure S6, related to Figure 6.

***EZH2*-deficient human ESC can initiate differentiation, but cannot generate mature cell types.**

(A) *EZH2*$^{-/-}$ ESC fail to generate teratomas. Three teratomas were analysed for each ESC line. The ruler underneath provides a size reference.

(B) Representative images of haematoxylin and eosin staining of teratomas. Note that control ESC formed large teratomas in all experiments, whereas EZH2$^{-/-}$ ESC generated a very small mass in only one kidney. The presence of various tissue types are indicated with key shown underneath. *EZH2*$^{-/-}$ ESC are unable to form mature cell types that are abundant in teratomas derived from control ESC. Scale bars, 300µm.

(C) Flow cytometry analysis confirms that C-KIT/CXCR4 negative *EZH2*$^{-/-}$ ESC are able to form C-KIT/CXCR4 positive endoderm progenitors after five days of differentiation. This experiment confirms that endoderm cells generated from a bulk population of *EZH2*$^{-/-}$ ESC are not arising entirely from pre-existing endoderm-primed cells.

Supplemental Experimental Procedures

Retinoic Acid differentiation

ESC were seeded onto Vitronectin matrix in TeSR-E8 media in 12-well plates. After 48h, 5μM of retinoic acid (Sigma-Aldrich) was added to each well in N2B27 media (50% DMEM-F12 (Thermo Fisher Scientific), 50% Neurobasal (Thermo Fisher Scientific) supplemented with 0.5X B-27 Supplement (Thermo Fisher Scientific), 0.5X N2 Supplement (Thermo Fisher Scientific), 2mM L-Glutamine, 0.1mM β-mercaptoethanol and 1x Penicillin/Streptomycin. Media was changed every 24h and cells processed on day 5 after induction.

Endoderm differentiation

Endoderm differentiation was achieved following the protocol by Nostro and colleagues (Nostro et al., 2011) with minor modifications. Briefly, three days after passage, ESC were washed once with RPMI-1640 (Thermo Fisher Scientific) and incubated overnight in 5%CO_2;5%O_2 in Endoderm Aggregation Media (RPMI-1640 supplemented with 2mM L-Glutamine (Thermo Fisher Scientific), 450nM MTG (Sigma), 1X Penicillin/Streptomycin (Thermo Fisher Scientific), 50μg/ml Ascorbic Acid (Sigma), 0.25ng/ml BMP-4 (R&D Systems), 5ng/ml FGF-2 (WT–MRC Cambridge Stem Cell Institute), 100ng/ml activin A (WT–MRC Cambridge Stem Cell Institute) and 10ng/ml VEGF (R&D Systems)). The following day (designated day 0) the media was replaced with endoderm induction media (RPMI-1640 supplemented with 2mM L-Glutamine (Thermo Fisher Scientific), 450nM MTG (Sigma), 1X Penicillin/Streptomycin (Thermo Fisher Scientific), 50μg/ml Ascorbic Acid (Sigma), 100ng/ml activin A (WT–MRC Cambridge Stem Cell Institute), 25ng/ml Wnt3a (R&D Systems) and 0.2-2% FBS (Sigma). Media was changed every 48h and cells were typically harvested on day 5.

Mesoderm differentiation

Mesoderm differentiation was achieved following the protocol by Kennedy and colleagues (Kennedy et al., 2007) with minor modifications. Briefly, ESC colonies were lifted away from MEF using 1mg/ml Collagenase IV, washed, and the colonies triturated to obtain small clusters. The clusters were cultured overnight as embryoid bodies (EB) in a non-tissue culture treated Petri dish on a rocker at 30 rpm at 5%CO_2;5%O_2 in 12ml mesoderm aggregation media: STEMPRO34 (Thermo Fisher Scientific) supplemented with 2mM L-Glutamine (Thermo Fisher Scientific), 450nM MTG (Sigma), 1X Penicillin/Streptomycin (Thermo Fisher Scientific), 50μg/ml Ascorbic Acid (Sigma), 150mg/ml Apo-Transferrin (Sigma), 2ng/ml BMP-4 (R&D Systems). The following day, half of the media was discarded and replaced with 6ml fresh mesoderm aggregation media supplemented with 10ng/ml FGF-2. Eighteen hours later (designed as day 0), EBs were collected by settling and re-suspended in mesoderm induction media (STEMPRO 34 supplemented with 2mM L-Glutamine, 450nM MTG, 1X Penicillin/Streptomycin, 50 μg/ml Ascorbic Acid, 150mg/ml Apo-Transferrin, 60ng/ml BMP-4, 10ng/ml activinA, 5ng/ml FGF-2). Cells were typically harvested on day 2.

Ectoderm differentiation

Ectoderm differentiation was achieved following the protocol by Lee and colleagues (Lee et al., 2010). Briefly, ESC were seeded at a density of 10,000 cells per cm^2 on Matrigel in mTeSR1 media (StemCell Technologies) and incubated in 5%CO_2 in air (designated as day 0). Differentiation was initiated on day 2 by replacing the media with KSR media (Advanced DMEM with 15% KSR, 2mM L-Glutamine, 1X Penicillin/Streptomycin (all from Thermo Fisher Scientific), 0.1mM β-mercaptoethanol (Sigma-Aldrich), supplemented with 10μM SB431542 and 500ng/ml Noggin. On day 4, media was replaced with 75% KSR media / 25% N2 media (Neurobasal supplemented with N2, B27 (without RA) and 2mM L-Glutamine; all from Thermo Fisher Scientific) containing 10μM SB431542 and 500ng/ml Noggin. On day 6, media was replaced with 50% KSR media / 50% N2 media containing 10μM SB431542 and 500ng/ml Noggin, and on day 8, media was replaced with 25% KSR media / 75% N2 media containing 10μM SB431542 and 500ng/ml Noggin. Cells were typically harvested on day 10.

Teratoma formation

Teratoma formation assays were provided as a service by the WT–MRC Cambridge Stem Cell Institute in a designated facility under licenses granted by the UK Home Office. HESC were injected beneath the kidney capsule of immune-deficient mice (NOD/SCID) as 1 million cells per animal. Four hESC lines were tested, with one hESC line injected into one kidney per animal and a total of three animals used for each hESC line. Mice were male, 12-weeks old and obtained from Charles River. Randomisation, but not experimental 'blinding' to sample identity, was used for these studies. Teratomas were excised after 8 weeks, except for one teratoma from an *EZH2*$^{-/+}$ hESC line that was excised after 6 weeks due to animal health. Samples were fixed, embedded, sectioned, stained with hematoxylin and eosin and examined as a service by the Histopathology Facility at the University of Cambridge.

***EZH2* shRNA knockdown experiments**

EZH2 shRNA (clone ID: V3LHS_412598) and non-silencing shRNA were purchased from Dharmacon and cloned into Neomycin EF1α mCherry expression plasmid (Golding et al., 2010) using *MluI* and *XhoI*. WIBR3 and FiPS cells were dissociated into single cells using Accutase (Thermo Fisher Scientific). Cells (2 million) were nucleofected with 10μg

Neomycin EF1α *EZH2* shRNA mCherry, 10μg Neomycin EF1α non-silencing shRNA mCherry, or mock transfected. Cells were plated in ESC media, supplemented for the first 24h with 10μM Rho Kinase inhibitor (Sigma-Aldrich). 72h after nucleofection, mCherry-positive cells were sorted by FACS directly into Trizol LS.

Flow cytometry
Cells were washed once with PBS and incubated with 0.05% Trypsin (Thermo Fisher Scientific) for 5 minutes at 37°C. Media containing 5% FBS was added to inactivate the trypsin and samples triturated gently to form a single cell suspension. Cells were washed once with 2% FBS in PBS and collected by centrifugation at 1000 rpm for 5 minutes. Primary antibody (diluted in 2% FBS) was applied for 1 hr at 4°C in the dark, washed once more, and suspended in 400μl 2% FBS, 1ug/ml DAPI. Cells were analysed on a LSRII or Fortessa V (BD) or sorted on an Influx or Aria III (BD) at the Babraham Institute Flow Cytometry Facility.

Alkaline Phosphatase staining
SSEA4-positive live ESC were obtained by FACS and 6000 cells were seeded in ESC media onto MEF-coated 12-well plates or in mTeSR-E8 media onto Vitronectin-coated 12-well plates. Media was supplemented with 10μM Rho kinase inhibitor for the first 24h. Cells were grown for 7-10d until the appearance of visible colonies and stained for Alkaline Phosphatase activity (Sigma-Aldrich).

Crystal Violet staining
Media was removed and cells were fixed with 3:1 (v:v) methanol/acetic acid for 5 minutes. Fresh fix buffer added for a further 5 minutes and cells were washed once in water. Cells were dried for 30 minutes and then each well coated equally with 0.1% crystal violet solution in PBS for 10 minutes. Crystal violet solution was removed and the cells washed with water. Cells were then air-dried.

Immunofluorescent microscopy
ESC were seeded onto MEF-coated glass coverslips. Cells were fixed with 2% paraformaldehyde for 10 minutes at room temperature, washed in PBS then permeablised and blocked with 5% FBS, 0.1%Triton X-100 in PBS for 1 hour at room temperature. Cells were stained with primary antibody overnight at 4°C. Secondary antibody was applied for 4 hours at 4°C. Nuclei were stained with 0.2ng/ml DAPI (Sigma-Alrich). Images were acquired on an Olympus FV1000 or NIKON A1-R confocal microscopes at the Babraham Institute Imaging Facility.

Western blot
Whole cell lysates were extracted in RIPA buffer. Proteins were separated by electrophoresis in 10-15% SDS-polyacrylamide gels and transferred to 0.45μM PVDF membranes (Amersham Hybond). Membranes were blocked for >1 h in TBS-T 5% milk and hybridised to primary antibody overnight at 4°C. Membranes were washed 3 times for 10 minutes in TBS-Tween 5% milk at room temperature then incubated for 1 h at room temperature with secondary antibodies HRP-conjugated rabbit-anti-mouse or goat-anti-rabbit immunoglobulins (1:10,000 dilution, GE Healthcare). Detection was performed using ECL Primer Western Blotting Detection Reagent (Amersham).

RT-qPCR
Total RNA from bulk cells was isolated using the RNeasy kit (QIAGEN). Cells isolated by FACS went directly into TRIzol LS Reagent (Thermo Fisher Scientific) and total RNA was extracted followed by clean-up with the RNeasy kit (Qiagen). Total RNA (0.5-1μg) was reversed transcribed using the QuantiTect Reverse Transcription Kit (Qiagen). qPCR was performed using JumpStart Sybr Green (Sigma-Aldrich) in the following reaction: 6μl Sybr Green, 0.52μl water, 0.24μl 10mM forward primer, 0.24μl 10mM reverse primer, 250ng cDNA in a total volume of 12μl. The following primers were used: EZH2_qPCR ,EED_qPCR, SUZ12_qPCR, EZH1_qPCR, SOX17_qPCR, FOXA2_qPCR, GATA4_qPCR, GATA6_qPCR, SOX1_qPCR, NEUROD1_qPCR, OCT4_qPCR, NANOG_qPCR, SOX2_qPCR, HAND1_qPCR, TWIST1_qPCR, OLIG2_qPCR, SNAI1_qPCR, SNAI2_qPCR, PAX6_qPCR, GAPDH_qPCR and HMBS_qPCR.

RNA-Sequencing libraries
SSEA4-positive live ESC were isolated by FACS straight into TRIzol LS (Thermo Fisher Scientific). Total RNA was purified and then cleaned up using an RNeasy kit (Qiagen). Indexed mRNA-Seq libraries were constructed from 500ng total RNA using the Tru-Seq RNA Library Prep Kit v2 (Illumina). Library fragment size and concentration was determined using an Aglient Bioanalyzer 2100 and KAPA Library Quantification Kit (KAPA Biosystems). Samples were sequenced on Illumina HiSeq 1000/2500 as 100bp single-end libraries at the Babraham Institute Sequencing Facility.

For single-cell RNA-Seq experiments, individual SSEA4-positive live ESC were deposited by FACS directly into 96-well plates. Poly-A cDNA from single cells was obtained as described (Picelli et al., 2014). For library generation the Illumina Nextera DNA LT library preparation kit was used following the Fluidigm protocol described for cDNA generation using the Fluidigm C1 machine (available online). Pooled libraries were sequenced on an Illumina HiSeq

1000/2500 as 100bp paired-end libraries at the Babraham Institute Sequencing Facility. Spike-ins (ERCC RNA Spike-in Mix, Ambion) were added in a final dilution of 1:10E6.

RNA-Sequencing analysis
Reads were trimmed using trim galore v0.4 (http://www.bioinformatics.babraham.ac.uk/projects/trim_galore/) using default parameters to remove the standard Illumina adapter sequence. Reads were mapped to the human GRCh38 genome assembly using tophat 2.0.12 guided by the gene models from the Ensembl v70 release. BAM files were imported to Seqmonk v32.1 (http://www.bioinformatics.babraham.ac.uk/projects/seqmonk/). Raw read counts per transcript were calculated using the RNA-Seq quantitation pipeline on the Ensembl v70 gene set using non-directional counts. Differentially expressed genes were identified using DESeq2 with a cut-off of $p<0.05$ after multiple testing correction and without independent filtering. Correlation based hierarchical clustering of the DESeq2 hits was performed on log2 RPM (reads per million reads of library) values, which were normalised per-gene by subtracting the median value across all samples. Gene ontology (GO) functional annotation for the differentially expressed gene sets was done using the DAVID analysis tool (http://david.abcc.ncifcrf.gov/home.jsp). *P*-values were adjusted for multiple hypothesis testing using Bonferroni correction. Gene set enrichment analysis in Figure 3C was calculated using the GSEAPreranked tool within GSEA software (Subramanian et al., 2005) (www.broadinstitute.org/gsea). Input data were a ranked gene list ordered by fold-change expression between $EZH2^{-/-}$ ESC and $EZH2^{-/- +EZH2}$ ESC (n=20879), and a set of PRC2 targets (n=1299; defined by high EZH2 and H3K27me3 promoter-localised ChIP-Seq values in $EZH2^{+/+}$ ESC). Default settings were used with 1000 gene set permutations. The *EZH2* network in Figure 3E was analysed and visualized using Cytoscape (http://www.cytoscape.org). For Figure 3F, data were quantitated over 20bp running windows, separated by 10bp and the values were globally normalised to the data set with the highest coverage.

For single-cell RNA-Sequencing analysis, reads were trimmed using trim galore v0.4 (http://www.bioinformatics.babraham.ac.uk/projects/trim_galore/) using default parameters to remove the standard Illumina adapter sequence. Reads were mapped to the GRCh38 human genome assembly plus the ERCC spike-in control sequences using HiSat v2.0.1b guided by splice junctions imported from the gene models in Ensembl v78. Uniquely mapping reads were imported into SeqMonk v0.32.1 for analysis (http://www.bioinformatics.babraham.ac.uk/projects/seqmonk/). In initial QC, samples were removed where the percentage of reads coming from ERCC was >10% or the percentage of reads in genes was < 75% or the percentage of genes showing any signal was <15%. To remove potential ambiguity, samples were excluded if *EZH2* log2RPM > 1 in the $EZH2^{-/-}$ ESC set, and *EZH2* log2RPM < 2 in the $EZH2^{+/+}$ ESC set. Initial quantitation was performed on a gene set of annotated models with a biotype of protein_coding or lincRNA at a gene level counting reads overlapping any valid exon in each gene. Heatmaps were produced from hierarchical clustering of scaled RPM normalised single-cell RNA-Seq data using the R hclust function with the Ward's method and Euclidean distance correlations.

ChIP-Sequencing libraries
SSEA4-positive live ESC were isolated by FACS and snap frozen. Native ChIP for profiling histone marks was performed as described by Gilfillan and colleagues (Gilfillan et al., 2012) with minor modifications. Cell pellets (from 400,000 cells) were thawed and suspended in 95µl MNase Buffer (50mM Tris-HCl pH8, 1mM CaCl2, 0.2% Triton X-100) supplemented with 5mM Sodium Butyrate and 1X Complete EDTA-free Protease Inhibitor (Roche). Chromatin was digested with micrococcal nuclease (3U; NEB) for 8min at 37°C to obtain predominantly mononucleosomes and dinucleosomes. Stop buffer (10µl of 110mM Tris-HCl pH8, 5mM EDTA) was added to inactivate the reaction. Chromatin was solubilised by brief sonication using a Diagenode Bioruptor for 1min on high power. Chromatin was diluted in 100ml RIPA-IP buffer (280mM NaCl, 1.8% Triton X-100, 0.2% SDS, 0.2% Sodium Deoxycholate, 5mM EGTA supplemented with 5mM Sodium Butyrate and 1X Complete EDTA-free Protease Inhibitor) and insoluble material was removed by centrifugation at 14,000 rpm at 4°C for 15 minutes. Supernatant was transferred to a new tube, 10% was removed for the input sample, and the remaining chromatin was pre-cleared with 50µl pre-washed Protein A and Protein G Dynabeads (Thermo Fisher Scientific) at 4°C for 1 hour. Dynabeads were removed, the pre-cleared chromatin was diluted to 500µl in RIPA-IP buffer and split into 5 tubes. Chromatin was incubated overnight with histone antibodies at 4°C with rotation. Pre-washed Dynabeads (10µl) were added to each tube and incubated at 4°C for 2-3 hours with rotation. Beads were washed five times with RIPA buffer, once with LiCl buffer (250mM LiCl, 10mM Tris-HCl pH8, 0.5% NP-40, 0.5% Sodium Deoxycholate, 1mM EDTA) and once with 1xTE. Beads were suspended in 100µl 1X TE supplemented with 50µg Proteinase K and incubated at 55°C for 1 hour. ChIP and Input DNA were purified using Genomic DNA Clean and Concentrator columns (Zymo) and eluted in 50µl 1xTE. ChIP material was examined using a small aliquot of material by qPCR with primers ChIP_qPCR_MESP1, ChIP_qPCR_GAPDH, ChIP_qPCR_OCT4, ChIP_qPCR_TMEM255B, ChIP_qPCR_SOX17, ChIP_qPCR_GATA4 and ChIP_qPCR_Intergenic.

Crosslinked ChIP for profiling EZH2 occupancy was performed using iDeal ChIP-Seq kit for Transcription Factors (Diagenode).

Indexed ChIP-Seq libraries were generated with the NEBNext Master Kit (NEB) using NEBNext Multiplex Oligos for Illumina indexes (NEB). Library fragment size and concentration was determined using an Aglient Bioanalyzer 2100 and KAPA Library Quantification Kit (KAPA Biosystems). Samples were sequenced on Illumina HiSeq 1000/2500 as 100bp single-end libraries at the Babraham Institute Sequencing Facility.

ChIP-Sequencing analysis

Reads were trimmed using trim galore v0.4 (http://www.bioinformatics.babraham.ac.uk/projects/trim_galore/) using default parameters to remove the standard Illumina adapter sequence. They were mapped to the human GRCh38 genome assembly using bowtie2 v2.2.5 using default parameters. BAM files imported to Seqmonk and reads were extended by 200bp at their 5' end to approximate the true insert size. For Figure 2A, gene promoters were assigned with high, intermediate and low CpG states as previously described by Mikkelsen and colleagues (Mikkelsen et al., 2007). Sequence reads were quantitated over 100bp running windows and the values were globally normalised to the data set with the highest coverage. Quantitation trend plot was used to calculate the mean signal over an average gene body -/+ 5kb for each of the three promoter states. For Figure 2B, non-duplicated reads were quantified using globally normalised read counts within probes -/+ 2.5kb of annotated transcriptional start sites. From this set, H3K27me3^{WT} promoters were defined as probes <0.1 log2 RPKM in input sample and >0.1 log2 RPKM in H2K27me3 IP sample. Probes were name-matched to genes and deduplicated by name. For Figure 3F and Figure S5, data were quantitated over 20bp running windows, separated by 10bp and the values were globally normalised to the data set with the highest coverage. For Figure S3B, 150Mb of Chromosome 5 (arbitrarily chosen) was divided into 300bp bins with 150bp step size, and the number of non-duplicated reads per bin was quantified and globally normalised to the data set with the highest coverage. For Figure 3, EZH2-target promoters were identified as follows: non-duplicated reads were quantified within probes -/+ 2.5kb of annotated transcriptional start sites, and probes with a globally normalised log2 read count between 7.25 and 10.5 were retained. Probes were name-matched to genes and deduplicated by name.

Antibodies

The following antibodies were used for flow cytometry: SSEA4 (R&D Systems, FAB1435), KDR-APC (R&D Systems, FAB357A), PDGFRα-PE (R&D Systems, FAB1264P), C-KIT-APC (Thermo Fisher Scientific, CD11705), CXCR4-PE (R&D Systems, FAB170B), EPCAM-APC (BioLegend, 324212), CD56-PE (BD Biosciences, 345812), Mouse IgG_{I}.APC (R&D Systems, IC002A), Mouse IgG_{2A} PE (R&D Systems, IC003P) and Mouse IgG_{I} PE (R&D Systems, IC002P). The following antibodies were used for immunofluorescent microscopy: SOX17 (R&D Systems, AF1924), OCT4 (Santa Cruz, sc5279), EZH2 C-terminal (Diagenode, pAB-039-050), EZH2 N-terminal (Sigma-Aldrich, E6909), H3K27me3 (Millipore, 07-449), H3K27me2 (Activ Motif, 39378), H3K27me1 (Activ Motif, 39246), H3S10ph (Millipore, 06-570), Donkey anti-goat AF488 (Thermo Fisher Scientific, A11055), Donkey anti-rabbit AF555 (Thermo Fisher Scientific, A31572), Goat anti-mouse AF568 (Thermo Fisher Scientific, A11031), Donkey anti-mouse AF647 (Thermo Fisher Scientific, A31571), Donkey anti-rabbit AF488 (Thermo Fisher Scientific, A21206). The following antibodies were used for ChIP: H3K27me3 (Millipore, 07-449; 1μg per ChIP), H4K4me3 (Abcam, ab8580; 0.5μg per ChIP), H3K27ac (Abcam ab4729; 0.5μg per ChIP), H3K4me1 (Abcam, ab8895; 0.5ug per ChIP), rabbit anti-mouse IgG (Jackson Immuno Research, 315-005-003; 0.5μg per ChIP) and EZH2 (Diagenode, pAB-039-050; 2μg per ChIP). The following antibodies were used for Western blotting: EZH2 (Diagenode, pAB-039-050), SUZ12 (NEB, 3737), EED (Millipore, 09-774), EZH1 (Abcam, ab13665), OCT4 (Santa Cruz, sc5279) and β-actin (Sigma-Aldrich, A5441).

Primers

EZH2_attb_F	GGGGACAAGTTTGTACAAAAAAGCAGGCTCTATGGGCCAGACTGGGAAGAA
EZH2_attb_R	GGGGACCACTTTGTACAAGAAAGCTGGGTCTCAAGGGATTTCCATTTCTCTTTCGA
mCherry_Geno_F	CTACGACGCTGAGGTCAAGA
mCherry_Geno_R	GTGTAGTCCTCGTTGTGGGA
TET-Prom_Geno_F	GCACGTCTCCCTATCAGTGA
TET-Prom_Geno_R	CCCGGTGTCTTCTATGGAGG
Ezh2_Exon7_F	TCAGCTTTGTTATAGAGACATAATTGG
Ezh2_Exon7_F	GGCTCATCCGCTACATTGAT
Ezh2_qPCR_F	GAGCAAAGCTTACACTCCTTTCA
Ezh2_qPCR_R	ATAAGTGTTGGGTGTTGCATGA
Suz12_qPCR_F	TTCTTCGAACTCGGAATCTCAT
Suz12_qPCR_R	TGATGTTTGTTCTGGAGTTTCG
Eed_qPCR_F	TTGCATTGGGCAATCAAGTT
Eed_qPCR_R	GCAGCACCACATTTATGATGAG
Ezh1_qPCR_F	GGCGCTGCTTTAAATACGACT
Ezh1_qPCR_R	CACATGGTTCTGGTTCAATCTT
Sox17_qPCR_F	CAGAATCCAGACCTGCACAAC
Sox17_qPCR_R	CTCTGCCTCCTCCACGAAG
Foxa2_qPCR_F	GTCCGACTGGAGCAGCTACTAT

Foxa2_qPCR_R	GTCCGACTGGAGCAGCTACTAT
Gata4_qPCR_F	GAAGCCCAAGAACCTGAATAAA
Gata4_qPCR_R	GTTGCTGGAGTTGCTGGAAG
Gata6_qPCR_F	GTGCCCAGACCACTTGCTAT
Gata6_qPCR_R	TGGAGTCATGGGAATGGAAT
Gapdh_qPCR_F	CGCTGAGTACGTCGTGGAGT
Gapdh_qPCR_R	GGGCAGAGATGATGACCCTTT
Hmbs_qPCR_F	AGGAGTTCAGTGCCATCATCCT
Hmbs_qPCR_R	CACAGCATACATGCATTCCTCA
Sox1_qPCR_F	ATGAAGGAGCACCCGGATTA
Sox1_qPCR_R	GCCAGCGAGTACTTGTCCTT
Pax6_qPCR_F	GGTTGGTATCCGGGGACTTC
Pax6_qPCR_R	CGTTGGAACTGATGGAGTTGGT
Neurod1_qPCR_F	CGAAGATGAGGACCTGGAAG
Neurod1_qPCR_R	CAGGCGAGCCTTAGTCATCT
Oct4_qPCR_F	GGATATACACAGGCCGATGTGG
Oct4_qPCR_R	ATGGTCGTTTGGCTGAATACCT
Nanog_qPCR_F	TCCAGCAGATGCAAGAACTCTC
Nanog_qPCR_R	GGTTCTGGAACCAGGTCTTCAC
Twist1_qPCR_F	CGGACAAGCTGAGCAAGATT
Twist1_qPCR_R	TGGAGGACCTGGTAGAGGAA
Hand1_qPCR_F	ACATCGCCTACCTGATGGAC
Hand1_qPCR_R	ATCCGCCTTCTTGAGTTCAG
Olig2_qPCR_F	GACAAGCTAGGAGGCAGTGG
Olig2_qPCR_R	CGGCTCTGTCATTTGCTTCT
Snai1_qPCR_F	TCAAGATGCACATCCGAAGC
Snai1_qPCR_R	CGGACATGGCCTTGTAGCAG
Snai2_qPCR_F	TCGGACCCACACATTACCTT
Snai2_qPCR_R	TGTGTCCTTGAAGCAACCAG
ChIP_qPCR_Intergenic_F	CGGATGACAGGGTTATTGCT
ChIP_qPCR_ Intergenic _R	GGATTCTGGGATCCTTGGTT
ChIP_qPCR_Gata4_F	TGAGAGGGTGTGCCAGAAACT
ChIP_qPCR_Gata4_R	GGGTTGCAAAGCGAATACATC
ChIP_qPCR_Sox17_F	AGTTGAGTCCTGGGGGAAAAA
ChIP_qPCR_Sox17_R	ACTGCATGGGCATCTTCAAAT
ChIP_qPCR_Mesp1_F	GTCTGCCAAGGAACCACTTC
ChIP_qPCR_Mesp1_R	CCCAAGTGACAAGGGACAAC
ChIP_qPCR_Oct4_F	CTCCCACACCTCCATGTTCT
ChIP_qPCR_Oct4_R	AGGAGCTGAGAGGGTGACTG
ChIP_qPCR_Tmem255b_F	CGGAATCTCCAATTCTTCCA
ChIP_qPCR_Tmem255b_R	AACTGGTGAACACGCAACAG
ChIP_qPCR_Gapdh_F	CAGGCTGGATGGAATGAAAG
ChIP_qPCR_Gapdh_R	AAAGGCACTCCTGGAAACCT

Supplemental References

Gilfillan, G.D., Hughes, T., Sheng, Y., Hjorthaug, H.S., Straub, T., Gervin, K., Harris, J.R., Undlien, D.E., and Lyle, R. (2012). Limitations and possibilities of low cell number ChIP-seq. BMC Genomics *13*, 645.

Golding, M.C., Zhang, L., and Mann M.R. (2010) Multiple epigenetic modifiers induce aggressive viral extinction in extraembryonic endoderm stem cells. Cell Stem Cell *6*:457-467.

Kennedy, M., D'Souza, S.L., Lynch-Kattman, M., Schwantz, S., and Keller, G. (2007). Development of the hemangioblast defines the onset of hematopoiesis in human ES cell differentiation cultures. Blood *109*, 2679-2687.

Lee, G., Chambers, S.M., Tomishima, M.J., and Studer, L. (2010). Derivation of neural crest cells from human pluripotent stem cells. Nat Protoc *5*, 688-701.

Mikkelsen, T.S., Ku, M., Jaffe, D.B., Issac, B., Lieberman, E., Giannoukos, G., Alvarez, P., Brockman, W., Kim, T.K., Koche, R.P., *et al.* (2007). Genome-wide maps of chromatin state in pluripotent and lineage-committed cells. Nature *448*, 553-560.

Nostro, M.C., Sarangi, F., Ogawa, S., Holtzinger, A., Corneo, B., Li, X., Micallef, S.J., Park, I.H., Basford, C., Wheeler, M.B., *et al.* (2011). Stage-specific signaling through TGFbeta family members and WNT regulates patterning and pancreatic specification of human pluripotent stem cells. Development *138*, 861-871.

Picelli, S., Faridani, O.R., Bjorklund, A.K., Winberg, G., Sagasser, S., and Sandberg, R. (2014). Full-length RNA-seq from single cells using Smart-seq2. Nat Protoc *9*, 171-181.

Subramanian, A., Tamayo, P., Mootha, V.K., Mukherjee, S., Ebert, B.L., Gillette, M.A., Paulovich, A., Pomeroy, S.L., Golub, T.R., Lander, E.S., *et al.* (2005). Gene set enrichment analysis: a knowledge-based approach for interpreting genome-wide expression profiles. Proc Natl Acad Sci U S A *102*, 15545-15550.
