# Supplementary material for: Deletion of the Polycomb-Group Protein EZH2 Leads to Compromised Self-Renewal and Differentiation Defects in Human Embryonic Stem Cells
Source: Cell Rep. 2016 Dec 6;17(10):2700–14. doi: 10.1016/j.celrep.2016.11.032 (PMC5177603; doi:10.1016/j.celrep.2016.11.032)
Supplement: Document S2. Article plus Supplemental Information [file mmc2.pdf]

Cell Reports

Article

Deletion of the Polycomb-Group Protein EZH2 Leads to Compromised Self-Renewal and Differentiation Defects in Human Embryonic Stem Cells

Graphical Abstract

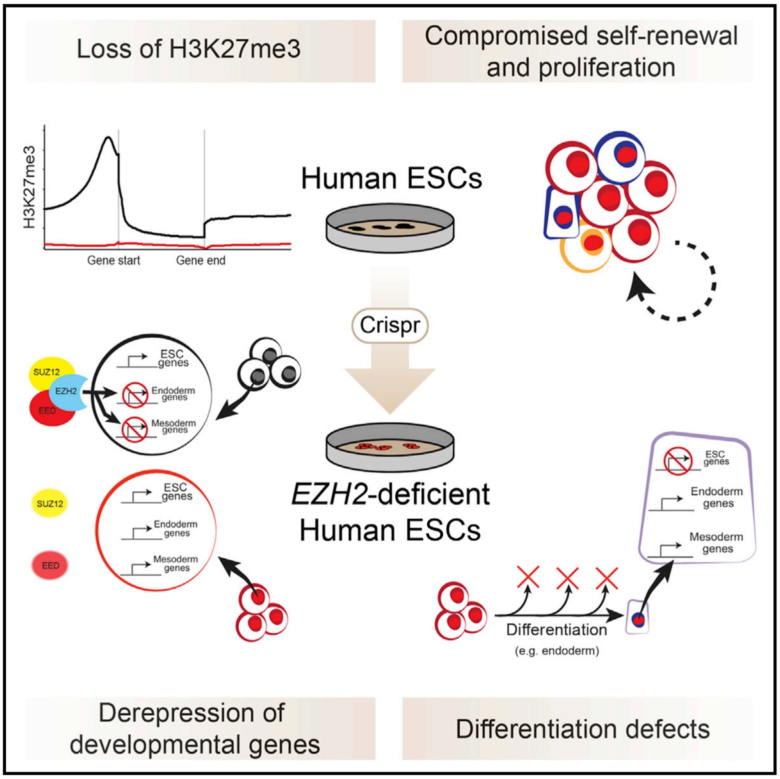

Highlights

- Comprehensive examination of *EZH2* function in human ESC regulation
- *EZH2* deficiency causes lineage-restricted derepression of developmental regulators
- More severe self-renewal and growth defects in *EZH2*-deficient hESCs than in mESCs
- *EZH2*-deficient hESCs can differentiate to early lineages but cannot form mature tissues

Authors

Adam Collinson, Amanda J. Collier, Natasha P. Morgan, Arnold R. Sienerth, Tamir Chandra, Simon Andrews, Peter J. Rugg-Gunn

Correspondence

peter.rugg-gunn@babraham.ac.uk

In Brief

Collinson et al. use *EZH2*-deficient human ESCs to demonstrate the broad conservation of Polycomb-group protein function in controlling cell-fate decisions and transcriptional programs during early human development. The authors also uncover unexpected human-specific differences that result in a more severe self-renewal and proliferation phenotype than that of PRC2-deficient mouse ESCs.

Accession Numbers

GSE76626

CrossMark

Collinson et al., 2016, Cell Reports *17*, 2700–2714
December 6, 2016 © 2016 The Author(s).
http://dx.doi.org/10.1016/j.celrep.2016.11.032

CellPress

OPEN ACCESS
CellPress

Cell Reports
Article

Deletion of the Polycomb-Group Protein EZH2 Leads to Compromised Self-Renewal and Differentiation Defects in Human Embryonic Stem Cells

Adam Collinson,[1] Amanda J. Collier,[1,2] Natasha P. Morgan,[1] Arnold R. Sienerth,[1] Tamir Chandra,[1] Simon Andrews,[3] and Peter J. Rugg-Gunn[1,2,4,5,*]
[1]Epigenetics Programme, The Babraham Institute, Cambridge CB22 3AT, UK
[2]Wellcome Trust – Medical Research Council Stem Cell Institute, University of Cambridge, Cambridge CB2 1QR, UK
[3]Bioinformatics Group, The Babraham Institute, Cambridge CB22 3AT, UK
[4]Centre for Trophoblast Research, University of Cambridge, Cambridge CB2 3EG, UK
[5]Lead Contact
*Correspondence: peter.rugg-gunn@babraham.ac.uk
http://dx.doi.org/10.1016/j.celrep.2016.11.032

SUMMARY

Through the histone methyltransferase EZH2, the Polycomb complex PRC2 mediates H3K27me3 and is associated with transcriptional repression. PRC2 regulates cell-fate decisions in model organisms; however, its role in regulating cell differentiation during human embryogenesis is unknown. Here, we report the characterization of *EZH2*-deficient human embryonic stem cells (hESCs). H3K27me3 was lost upon *EZH2* deletion, identifying an essential requirement for EZH2 in methylating H3K27 in hESCs, in contrast to its non-essential role in mouse ESCs. Developmental regulators were derepressed in *EZH2*-deficient hESCs, and single-cell analysis revealed an unexpected acquisition of lineage-restricted transcriptional programs. *EZH2*-deficient hESCs show strongly reduced self-renewal and proliferation, thereby identifying a more severe phenotype compared to mouse ESCs. *EZH2*-deficient hESCs can initiate differentiation toward developmental lineages; however, they cannot fully differentiate into mature specialized tissues. Thus, *EZH2* is required for stable ESC self-renewal, regulation of transcriptional programs, and for late-stage differentiation in this model of early human development.

INTRODUCTION

Polycomb-group (PcG) proteins are epigenetic repressors of transcriptional programs and maintain cellular identity during development, differentiation, and disease (Di Croce and Helin, 2013; Pasini and Di Croce, 2016; Pietersen and van Lohuizen, 2008; Schuettengruber and Cavalli, 2009; Surface et al., 2010). PcG proteins form two well-characterized and biochemically distinct chromatin-modifying complexes that are termed Polycomb Repressive Complex 1 and 2 (PRC1 and PRC2). PRC1 catalyzes histone H2A lysine 119 ubiquitination through the activity of the E3 ligases RING1A and RING1B (Müller and Verrijzer, 2009; Wang et al., 2004). PRC2 is composed of the core proteins EZH2, EED, and SUZ12, together with RBAP46/48 and several other accessory subunits, and is responsible for catalyzing di- and trimethylation on histone H3 lysine 27 (H3K27me2/3) (Cao et al., 2002; Czermin et al., 2002; Kuzmichev et al., 2002; Margueron and Reinberg, 2011; Müller et al., 2002). EZH2 is a SET-domain containing histone methyltransferase and is the catalytic subunit of PRC2. EED and SUZ12 are required for substrate recognition, complex stability and for promoting the enzymatic activity of EZH2 (Cao and Zhang, 2004; Nekrasov et al., 2005; Pasini et al., 2004; Tie et al., 2007).

Genome-wide studies in mouse and human embryonic stem cells (ESCs) have shown that PRC2 and H3K27me3 occupy the promoters of many developmental regulators that are important for cell differentiation and lineage specification (Azuara et al., 2006; Bernstein et al., 2006; Boyer et al., 2006; Bracken et al., 2006; Lee et al., 2006; Mikkelsen et al., 2007; Pan et al., 2007; Zhao et al., 2007). This distribution of chromatin marks led to the concept that PRC2 may contribute to the maintenance of pluripotency by keeping developmental regulators transcriptionally repressed, while enabling the genes to be rapidly activated upon suitable differentiation cues and stimuli. Despite a central position within the regulatory framework, however, PRC2 is dispensable for the maintenance of undifferentiated mouse ESCs, as the deletion of PRC2 components, including *Ezh2*, has little effect on their morphology, self-renewal, or proliferation, although a subset of PRC2 target genes are modestly derepressed (Chamberlain et al., 2008; Leeb et al., 2010; Pasini et al., 2007; Riising et al., 2014; Shen et al., 2008). H3K27me3 levels are globally reduced in *Ezh2*-deficient mouse ESCs; however, developmental regulators retain H3K27me3 at their gene promoters and are transcriptionally repressed (Shen et al., 2008). In this context, the Ezh2 homolog, Ezh1, forms a noncanonical PRC2 complex that is able to trimethylate H3K27 at target gene promoters and maintains transcriptional repression through methylation-dependent and potentially methylation-independent pathways (Margueron et al., 2008; Shen et al., 2008).

 © 2016 The Author(s).
This is an open access article under the CC BY license (http://creativecommons.org/licenses/by/4.0/).

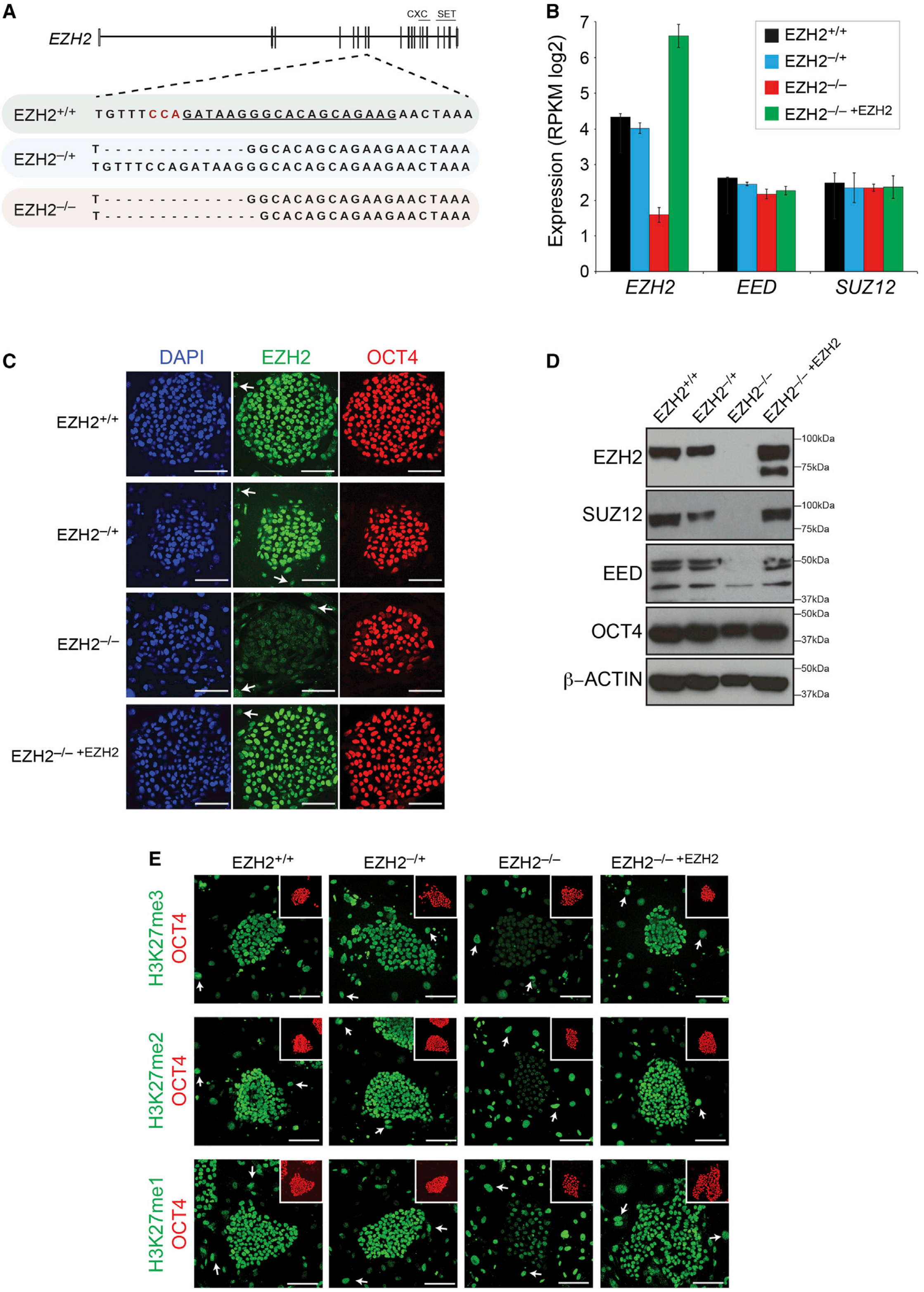

Figure 1. Targeted Deletion of *EZH2* in hESCs

(A) Overview of *EZH2* structure and targeting strategy. Exons encoding CXC and SET domains are indicated. The gRNA sequence is underlined and protospacer adjacent motif highlighted in red. DNA sequence of the deletions in one $EZH2^{-/+}$ ESC line and one $EZH2^{-/-}$ ESC line is shown for both alleles. Mutation causes frameshift and premature stop codon. An additional line is shown in Figures S1 and S2.

(legend continued on next page)

PRC2 deficiency has a more significant impact on mouse ESCs upon their differentiation, with defects in the repression of pluripotency networks and in the failure to fully activate differentiation transcriptional programs. This aberrant gene regulation results in impaired differentiation and proliferation (Chamberlain et al., 2008; Pasini et al., 2007; Shen et al., 2008). Further underscoring the critical role of PRC2 in directing differentiation programs, all three PRC2 core components (*Ezh2*, *Eed*, and *Suz12*) are essential for early mouse development, as loss-of-function mutant embryos initiate but fail to complete gastrulation and die between embryonic days 7 and 9 (Faust et al., 1995; O'Carroll et al., 2001; Pasini et al., 2004). The mutant phenotype is associated with mis-expression of lineage-specifying genes, decreased cell proliferation and an increased level of apoptosis (Pasini et al., 2004).

The well-conserved binding profiles of PRC2 components and H3K27me3 in human ESCs (hESCs) at the promoters of developmental regulators raises the possibility that PRC2 may also have an important role in controlling hESC pluripotency and differentiation (Gifford et al., 2013; Ku et al., 2008; Pan et al., 2007; Zhao et al., 2007). Moreover, coordinated changes to the epigenome, including H3K27me3 localization, occur upon differentiation of hESCs and are thought to be essential for lineage specification and memory of cellular identity, as they are in *Drosophila* and the mouse (Gifford et al., 2013; Xie et al., 2013). However, no functional studies of PRC2 in hESC regulation and early-stage differentiation have been reported to date. Human pluripotent cells represent a unique model in which to study human development and provide a platform for producing a source of differentiated cells relevant for basic and applied research. In addition, mouse and human ESCs are known to represent different pluripotent states and may therefore rely on different epigenetic pathways to confer their ability to self-renew and differentiate (Nichols and Smith, 2009; Rossant, 2015). Understanding the key epigenetic mechanisms that underpin hESCs is therefore a priority.

Here, we report the generation and characterization of *EZH2*-deficient hESCs. Our findings demonstrate that EZH2 is required to maintain the transcriptional repression of developmental regulators and for cells to undergo late-stage cell differentiation, thereby revealing the broad conservation of PRC2 function in this model of early human development. We also identify unexpected human-specific differences such as the essential requirement in hESC for EZH2 to maintain PRC2 stability and retain promoter-localized H3K27me3, which is in contrast to its non-essential role in mouse ESCs. In addition, self-renewal and proliferation are also perturbed to a greater extent in *EZH2*-deficient hESCs, as compared to *Ezh2*-deficient mouse ESCs. Our study therefore provides a comprehensive characterization of PRC2 function in hESCs, thereby providing a new platform to investigate the role of histone methylation in regulating the genome during human development and stem cell differentiation.

RESULTS

Targeted Deletion of *EZH2* in hESCs

To investigate the role of *EZH2* in human pluripotency and differentiation, we used CRISPR/Cas9 to disrupt *EZH2* in hESCs. A guide RNA (gRNA) designed to target an early exon within all known *EZH2* isoforms was nucleofected with *Cas9* into the H9 hESC line (Figures 1A and S1A). Individual colonies were isolated, expanded, and analyzed by Sanger DNA sequencing. The efficiency of disrupting the target sequence within the *EZH2* coding region was high, with ~35% clonal lines containing a mutation on one allele (*EZH2*$^{-/+}$). However, no homozygous cell lines were obtained out of 110 screened lines. This result provided a first indication that *EZH2*-deficient hESCs may be compromised relative to *EZH2*-containing cells when plated as single cells at clonal density. To overcome this apparent defect, we introduced a doxycycline (DOX)-inducible *EZH2* transgene using piggyBac transposition into an *EZH2*$^{-/+}$ line and re-targeted the cells with *EZH2* gRNA and *Cas9* in the presence of DOX. Using this strategy, we obtained several *EZH2* homozygous lines (*EZH2*$^{-/-}$; Figures 1A, S1B, and S1C). Once the *EZH2*$^{-/-}$ lines were isolated and established, they could be maintained without DOX-induced *EZH2* expression. Although we did not detect any indication that the DOX-inducible plasmid was leaky in the absence of DOX, to rule out the possibility of low-level *EZH2* expression, we transiently transfected *EZH2*$^{-/-}$ ESCs with piggyBac transposase and obtained stable *EZH2*$^{-/-}$ lines with all copies of the *EZH2* transgene removed (Figures S2A and S2B).

RNA expression analysis confirmed that *EZH2* transcripts were lower in *EZH2*$^{-/-}$ ESCs compared to parental *EZH2*$^{+/+}$ and *EZH2*$^{-/+}$ lines (Figures 1B and S2C). Moreover, EZH2 protein was undetectable by western blot and by immunofluorescent microscopy using two different antibodies raised against N- and C-terminal epitopes of EZH2 (Figures 1C, 1D, and S2D–S2F). The disruption of *EZH2* was accompanied by the loss of other PRC2 proteins, SUZ12 and EED, despite the presence of unchanged levels of *SUZ12* and *EED* transcripts in *EZH2*$^{-/-}$ ESCs (Figures 1B and 1D). This finding unexpectedly contrasts with *Ezh2*-deficient mouse ESCs where Suz12 and Eed levels are unchanged due to the ability of Ezh1 to form non-canonical PRC2 (Shen et al., 2008) but is consistent with *Suz12*-deficient and *Eed*-deficient mouse ESCs in which PRC2 components are unstable outside of the complex (Pasini et al.,

(B) mRNA expression levels from RNA-seq data revealing *EZH2*, *EED,* and *SUZ12* transcript levels in *EZH2*$^{+/+}$, *EZH2*$^{-/+}$, *EZH2*$^{-/-}$, and *EZH2*$^{-/-\ +EZH2}$ ESCs. Data show mean ± SD; n = 3 biological replicates.

(C) Immunofluorescent microscopy of colonies from *EZH2*$^{-/-}$ ESCs and control ESCs. This analysis reveals a strong reduction in EZH2 levels in *EZH2*$^{-/-}$ ESCs. The antibody was raised against a C-terminal epitope of EZH2; similar results were obtained using an alternative antibody raised against the N-terminal of EZH2 (Figure S2E). OCT4 expression indicates undifferentiated cells within a hESC colony. Arrows point to MEF. Scale bars, 100 μm.

(D) EZH2, SUZ12, and the main isoform of EED are undetectable in *EZH2*$^{-/-}$ ESCs by western blot analysis and are restored upon expression of a DOX-induced *EZH2* transgene. β-ACTIN is the loading control. Mass is in kilodaltons.

(E) H3K27me3 and H3K27me2 levels are reduced to background levels, and H3K27me1 levels are partially reduced, in *EZH2*$^{-/-}$ ESCs. OCT4 expression in inset indicates undifferentiated ESCs within the field of view. Arrows point to MEF. Scale bars, 100 μm.

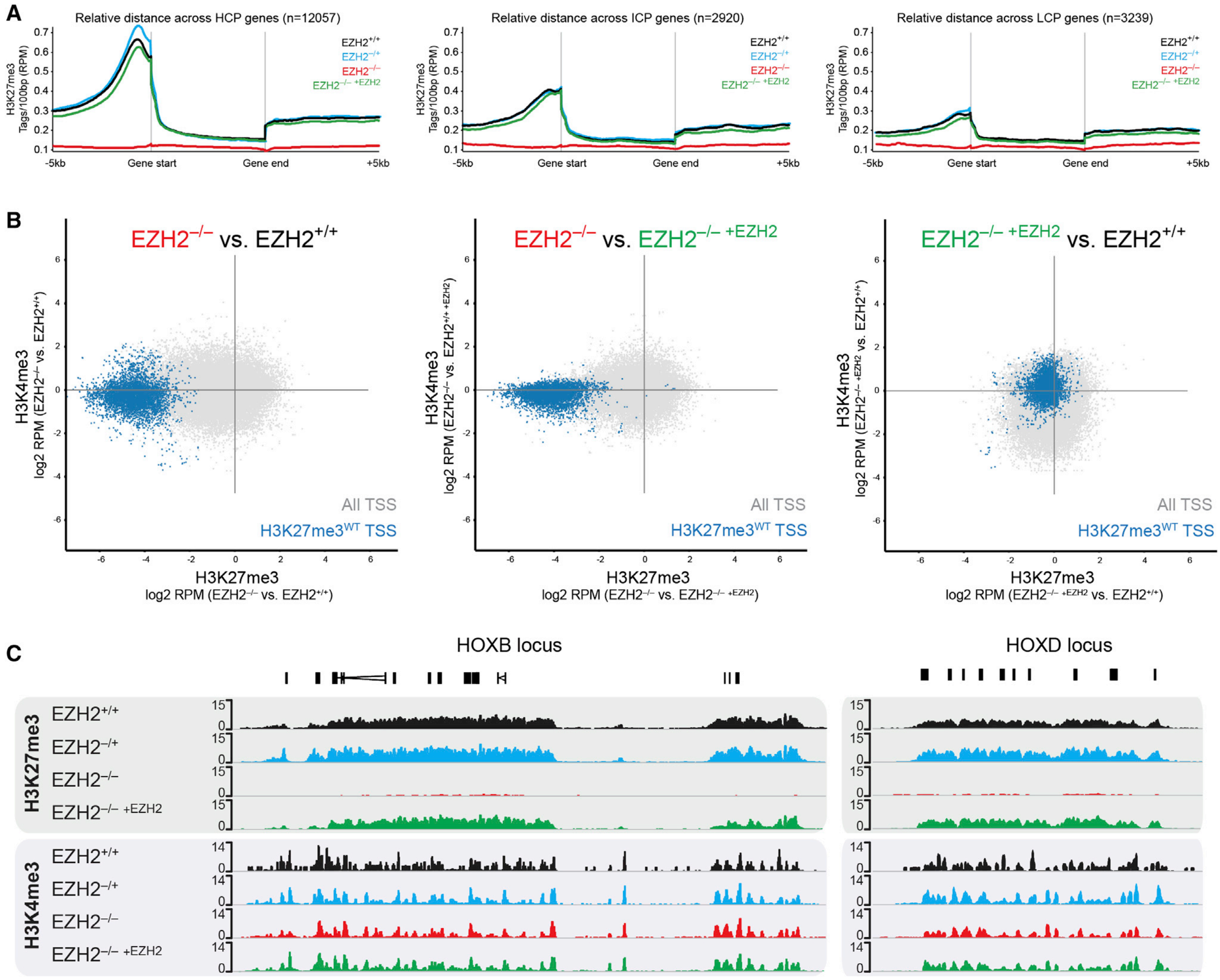

Figure 2. *EZH2* Deficiency in hESCs Results in Loss of H3K27me3

(A) Quantitative trend plot of H3K27me3 normalized ChIP-seq reads over gene body ±5 kb. High CpG (HCP), intermediate CpG (ICP), and low CpG (LCP) promoters are shown separately.

(B) Scatterplot of H3K27me3 (x axis) and H3K4me3 (y axis) normalized ChIP-seq reads in $EZH2^{-/-}$ relative to $EZH2^{+/+}$ (left) and relative to $EZH2^{-/-\ +EZH2}$ (center), and $EZH2^{-/-\ +EZH2}$ versus $EZH2^{+/+}$ (right). All transcriptional start sites (TSS) shown in gray; TSS that are positive for H3K27me3 in $EZH2^{+/+}$ ESCs highlighted in blue. Disruption of *EZH2* leads to a strong reduction in H3K27me3 levels at TSS, with little effect on H3K4me3 levels. Expression of a DOX-mediated *EZH2* transgene in the *EZH2*-deficient cells causes restoration of H3K27me3 levels to levels equivalent to $EZH2^{+/+}$.

(C) ChIP-seq tracks of *HOXB* (left) and *HOXD* (right) loci illustrate the loss of H3K27me3 in $EZH2^{-/-}$ ESCs compared to control ESCs. H3K4me3 is relatively unaffected. All ChIP-seq data represent the average of three biological replicates for each cell line. These results were confirmed independently by qPCR analysis of ChIP DNA at several gene promoters (Figure S3C).

2004, 2007). In hESCs, therefore, EZH1 is unable to form noncanonical PRC2 despite being present (Figure S2G). *EZH1* transcript and protein levels were largely unchanged upon *EZH2* deletion (Figure S2G). Immunofluorescent microscopy revealed that the loss of *EZH2* led to the reduction of H3K27me3 and H3K27me2 to background levels, and to the partial reduction of H3K27me1 (Figure 1E). Applying DOX to induce ectopic *EZH2* expression in $EZH2^{-/-}$ ESCs restored EZH2 and led to the stabilization of SUZ12 and EED proteins, and to the re-establishment of global H3K27 methylation (cells designated herein as $EZH2^{-/-\ +EZH2}$; Figures 1B–1E).

Loss of Promoter-Localized H3K27me3 in *EZH2*-Deficient hESCs

To characterize the molecular phenotype of *EZH2*-deficient hESCs, we profiled genome-wide histone methylation by native chromatin immunoprecipitation combined with high-throughput sequencing (chromatin immunoprecipitation sequencing [ChIP-seq]). Quantitative trend plots of normalized ChIP-seq reads revealed a complete loss of H3K27me3 at all gene promoters in $EZH2^{-/-}$ ESCs (Figure 2A). This finding contrasts with the retention of H3K27me3 at the promoters of developmental regulators in *Ezh2*-deficient mouse ESCs (Shen et al., 2008).

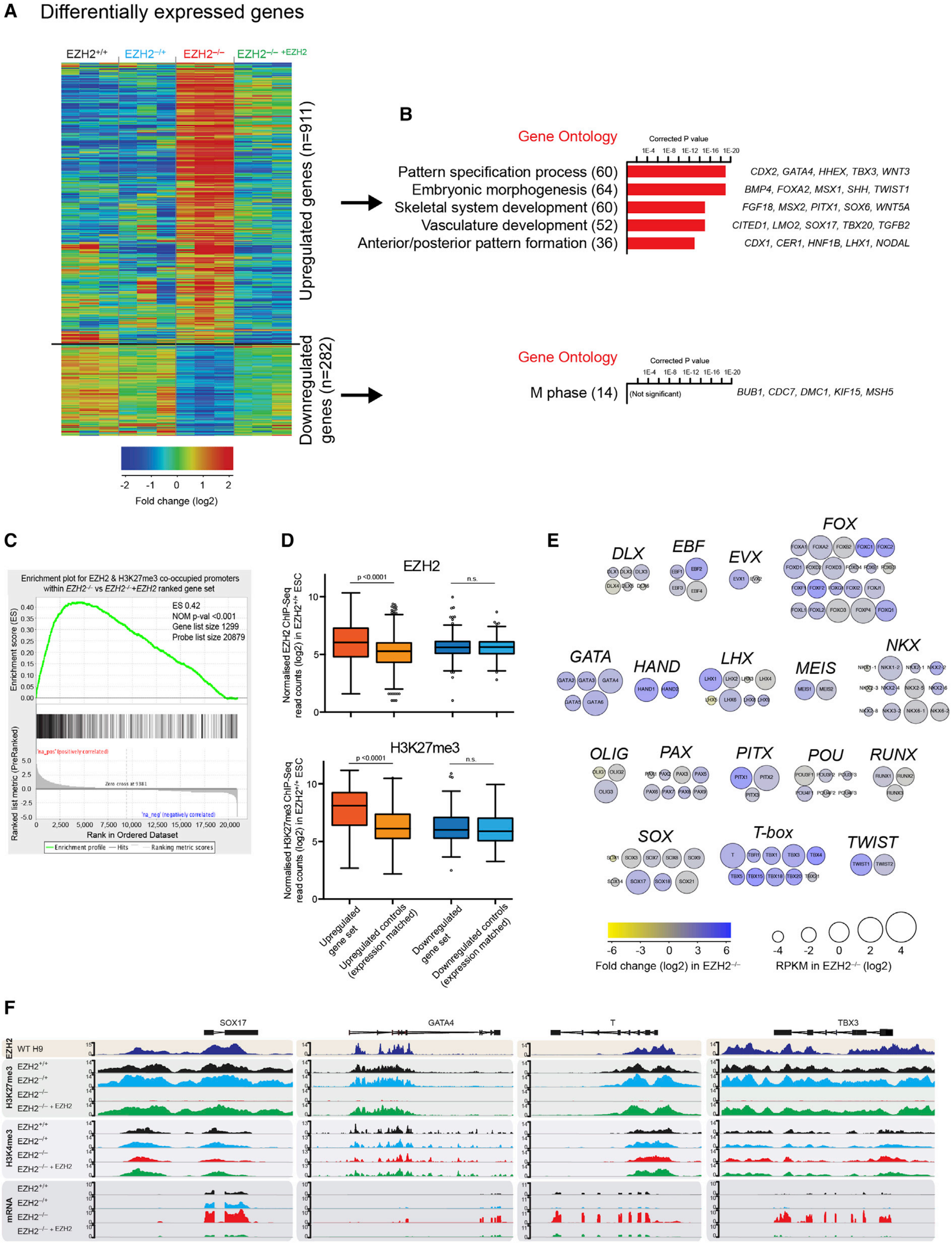

Figure 3. Genes Encoding Developmental Regulators Are Transcriptionally Derepressed in *EZH2*-Deficient hESCs

(A) RNA-seq heatmap for *EZH2*$^{-/-}$ ESCs and control ESCs (three biological replicates per line). Shown are all differentially expressed genes between *EZH2*$^{-/-}$and *EZH2*$^{-/-\ +EZH2}$ ESCs.

(legend continued on next page)

Confirming the result in hESCs, scatterplot analysis of ~2,000 promoters that have high levels of H3K27me3 in *EZH2*$^{+/+}$ ESCs (H3K27me3^{WT}) revealed a loss of H3K27me3 in *EZH2*$^{-/-}$ ESCs (Figure 2B). The majority of H3K27me3^{WT} promoters have histone H3 lysine 4 trimethylation (H3K4me3) in hESCs (Pan et al., 2007; Zhao et al., 2007), and H3K4me3 levels were largely unaffected by *EZH2* disruption (Figure 2B). ChIP-seq tracks for two example loci, *HOXB* and *HOXD*, illustrate the loss of H3K27me3 across the domains in *EZH2*$^{-/-}$ ESCs (Figure 2C). Comparison of H3K27me3 between *EZH2*$^{+/+}$ and *EZH2*$^{-/-\ +EZH2}$ cells revealed highly similar profiles, demonstrating that histone patterns are appropriately re-established upon EZH2 restoration (Figures 2A–2C). Interestingly, there was a modest increase in histone H3 lysine 27 acetylation (H3K27ac) levels at H3K27me3^{WT} promoters in *EZH2*$^{-/-}$ ESCs, supporting a potential antagonism between H3K27 acetylation and trimethylation that has also been observed in other contexts (Ferrari et al., 2014; Gehani et al., 2010; Jung et al., 2010; Pasini et al., 2010; Schoenfelder et al., 2015) (Figure S3A). Last, an alternative ChIP-seq analysis strategy of genome binning confirmed a loss of H3K27me3 sequencing reads across the genome of *EZH2*$^{-/-}$ ESCs, further reinforcing the key finding that H3K27me3 levels are depleted upon deletion of *EZH2* in hESCs (Figure S3B). Together, these results demonstrate that EZH2 is the main functional H3K27me2/3 methyltransferase in hESCs.

EZH2 Deficiency Causes Transcriptional Derepression of Key Developmental Genes

We next performed RNA sequencing (RNA-seq) to investigate the impact of loss of EZH2 and associated H3K27me3 on gene expression. The assays were carried out on samples that were flow-sorted using the hESCs cell-surface marker SSEA4 to ensure that we compared between equivalent cell populations (Figure S4A). The majority of genes were not altered transcriptionally by *EZH2* disruption, but 911 genes were significantly upregulated and 282 genes were significantly downregulated in *EZH2*$^{-/-}$ ESCs compared to *EZH2*$^{-/-\ +EZH2}$ ESCs ($p < 0.05$; Figures 3A and S4B). Gene ontology (GO) analysis of the upregulated gene set identified categories associated with developmental and cellular differentiation, including pattern specification, embryonic morphogenesis, and tissue formation (Figure 3B). The upregulated group was significantly enriched for genes with EZH2 and H3K27me3 occupancy in *EZH2*$^{+/+}$ ESCs and are thus expected to be sensitive to *EZH2* disruption (Figures 3C and 3D). Notably, not all genes with EZH2-bound promoters were mis-regulated, suggesting that secondary events may be required for this group of genes to initiate transcriptional change in response to the loss of EZH2. In addition, a subset of upregulated genes (enriched for signaling and adhesion GO terms) are likely to be regulated indirectly as they are not PRC2 targets in hESCs. No GO categories were significantly enriched in the downregulated gene set, although of note, the top category was associated with the regulation of M-phase and indicate the decreased transcription of genes typically expressed in mitosis (Figure 3B).

We examined transcriptional changes in a set of ~100 classical developmental regulators that have strong EZH2 promoter occupancy, including genes within the FOX, GATA, LHX, T-box, and SOX families (Lee et al., 2006). A clear pattern emerges from this analysis: nearly all genes within this class of developmental regulator showed transcriptional derepression in the absence of *EZH2* (Figures 3E and S4C). Gene derepression did not become more prevalent upon continued passaging, and therefore the *EZH2*$^{-/-}$ ESCs retained a similar transcriptional profile over time. ChIP-seq and RNA-seq tracks for several example genes, including *SOX17*, *GATA4*, *T*, and *TBX3*, illustrate the absence of promoter H3K27me3 and an associated increased transcript level in *EZH2*$^{-/-}$ ESCs (Figure 3F). We further showed that small hairpin RNA (shRNA)-mediated depletion of *EZH2* causes derepression of developmental regulators in two additional human pluripotent stem cell lines (WIBR3 and FiPS; Figure S4D). We conclude that the deletion of *EZH2* in hESCs leads to the loss of H3K27me3 and to the transcriptional derepression of genes that encode developmental regulators, thereby positioning EZH2 as a key factor in controlling the transcriptome of human cell types during early development.

Single-Cell Transcriptional Analysis Reveals Gene Mis-regulation Profiles

To investigate more precisely the transcriptional mis-regulation and cell-to-cell variability in response to *EZH2* deficiency, we performed single-cell RNA-seq on individual SSEA4-positive, flow-sorted *EZH2*$^{-/-}$ and *EZH2*$^{+/+}$ ESCs. The results show that a subset of *EZH2*$^{-/-}$ ESCs strongly upregulated EZH2-target genes, but individual genes are not robustly derepressed in most cells examined (Figure 4A). Unexpectedly, clustering of the data suggested that gene derepression occurs predominately within discrete transcriptional programs, such that

(B) Top GO terms of differentially expressed gene sets. Numbers of genes are shown; example genes within each GO category are listed (right). Corrected p values were calculated using a modified Fisher's exact test followed by Bonferroni's multiple comparison test.

(C) Gene set enrichment analysis of PRC2 targets (n = 1,299; defined by high EZH2 and H3K27me3 promoter-localized ChIP-seq values in *EZH2*$^{+/+}$ ESCs) in genes that have been ranked according to their fold change in transcription between *EZH2*$^{-/-}$ ESCs and *EZH2*$^{-/-\ +EZH2}$ ESCs. The positive enrichment score (ES) reveals that genes selectively derepressed in the absence of *EZH2* are enriched in PRC2 targets ($p < 0.001$; Kolmogorov-Smirnov statistic).

(D) Genes within the upregulated category have higher levels of promoter-localized EZH2 (upper) and H3K27me3 (lower) in EZH2$^{+/+}$ ESCs compared to an expression-matched set of genes and to downregulated genes. Data were compared using a Kruskal-Wallis test followed by Dunn's multiple comparison test.

(E) Genes encoding developmental regulators are transcriptionally derepressed in *EZH2*-deficient ESCs. A subset of direct EZH2 target genes is depicted as family groups. The color of each circle represents the log2 fold change in *EZH2*$^{-/-}$ ESCs relative to *EZH2*$^{-/-\ +EZH2}$ ESCs. The size of each circle represents the expression value of the gene in *EZH2*$^{-/-}$ cells. A similar pattern of target gene derepression is observed when comparing *EZH2*$^{-/-}$ ESCs with *EZH2*$^{+/+}$ ESCs (Figure S4C).

(F) ChIP-seq and mRNA-seq tracks of four genes encoding key developmental regulators illustrate the association between loss of H3K27me3 and transcriptional upregulation in *EZH2*$^{-/-}$ ESCs.

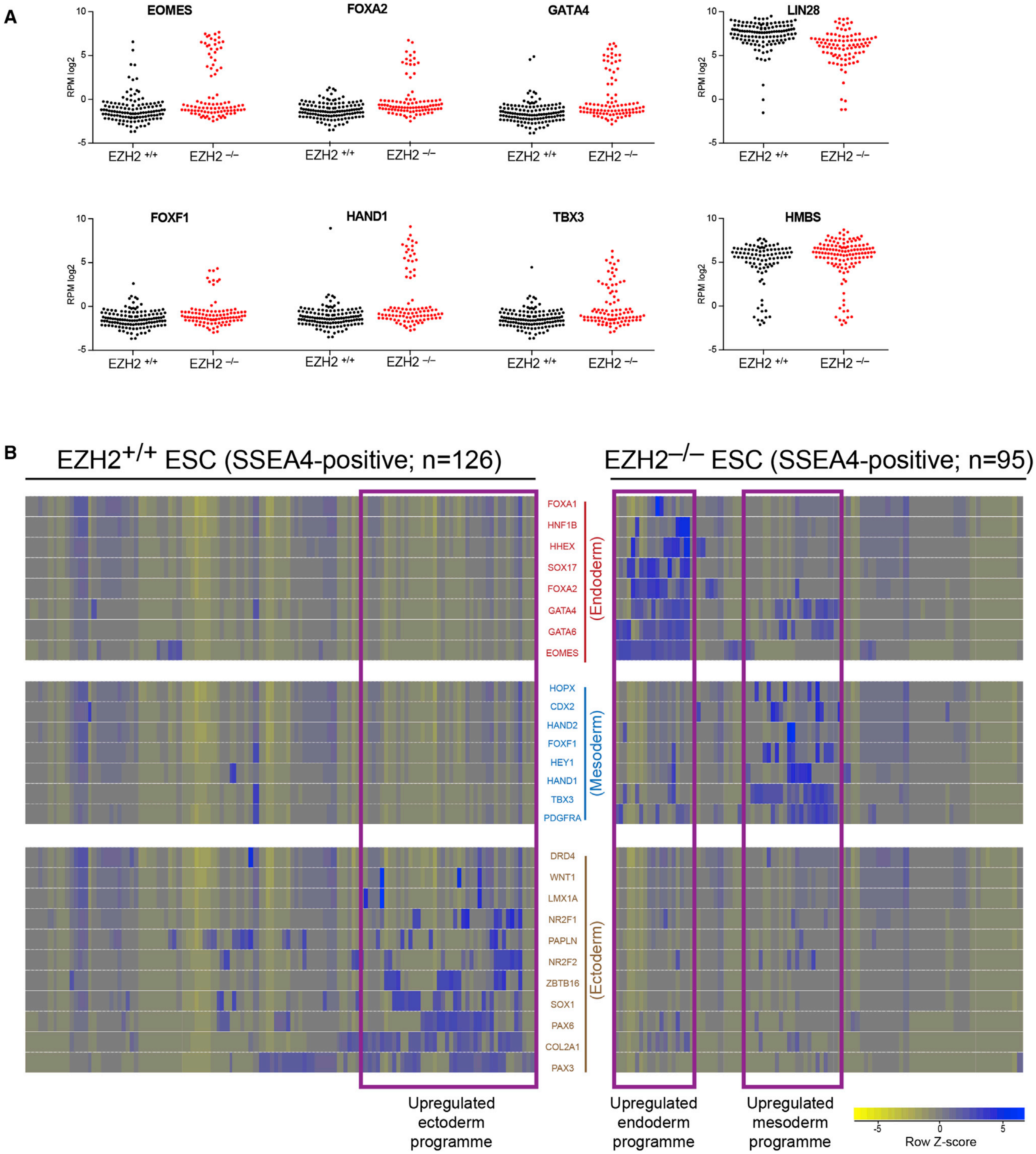

Figure 4. Transcriptional Derepression Occurs Predominantly within Discrete Lineage-Specific Programs

(A) Single-cell RNA-seq expression levels for six example PRC2-target genes in *EZH2*$^{-/-}$ ESCs and *EZH2*$^{+/+}$ ESCs, where each dot represent the results from a single cell. A pluripotency gene (*LIN28*) and housekeeping gene (*HMBS*) are shown for comparison. Robust upregulation of PRC2-target genes occurs in a subset of *EZH2*$^{-/-}$ ESCs.

(B) Heatmap of single-cell RNA-seq expression for *EZH2*$^{-/-}$ ESCs (right) and *EZH2*$^{+/+}$ ESCs (left). Each column represents an individual cell. Each row represents an individual gene, grouped into three clusters corresponding to endoderm, mesoderm, and ectoderm cell lineages. Shown are PRC2-target genes from within the hESC scorecard assay, which is an assay that can classify differentiated cell lineages (Bock et al., 2011). Subsets of cells (boxed in purple) tend to mis-express many genes from within one lineage but rarely mis-express multiple genes derived from more than one lineage.

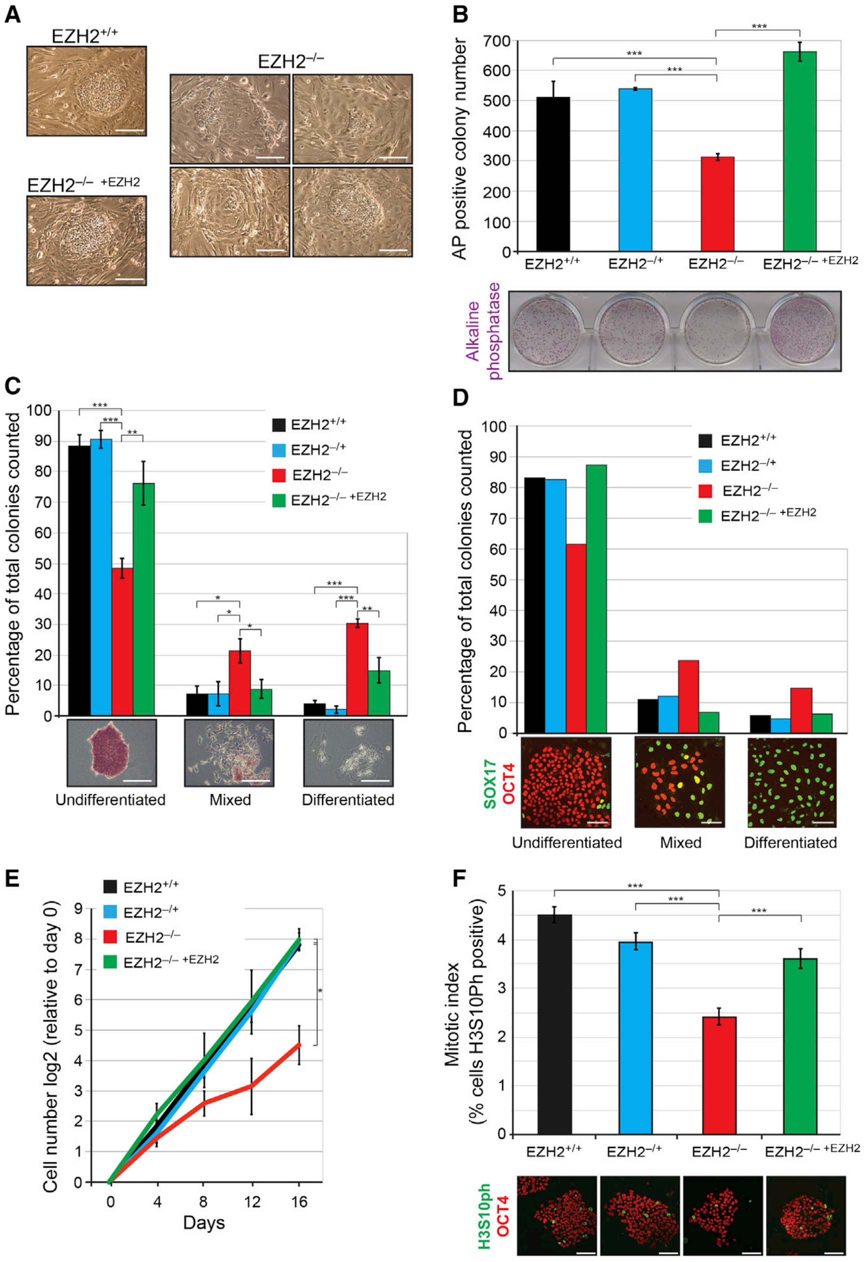

Figure 5. *EZH2*-Deficient hESCs Are Compromised in Self-Renewal and Proliferation

(A) Phase contrast images show representative colonies of *EZH2*$^{+/+}$, *EZH2*$^{-/-}$, and *EZH2*$^{-/-}$ $^{+EZH2}$ ESC lines. Note the variable morphology of *EZH2*-deficient colonies. Scale bars, 100 μm.

(B) *EZH2*$^{-/-}$ ESCs show reduced ESC colony formation when plated as single SSEA4-positive cells at low density (6,000 cells seeded per well). Data show mean ± SD; n = 3 biological replicates. Data were compared using a one-way ANOVA followed by Bonferroni's multiple comparison test (***$p < 0.0005$). Representative AP staining is shown underneath.

(C) *EZH2*$^{-/-}$ ESCs have reduced capacity to self-renew when plated at clonal density. ESC colonies were categorized as undifferentiated, mixed, or differentiated based on AP activity; examples shown underneath. Data show mean ± SD; n = 3 biological replicates. Over 150 colonies were scored for each cell line. Data were compared using a one-way ANOVA followed by Bonferroni's multiple comparison test (***$p < 0.0005$; **$p < 0.005$; *$p < 0.05$). Scale bars, 100 μm.

(D) Immunofluorescent microscopy for OCT4 (undifferentiated marker) and SOX17 (early differentiation marker) reveals an increased prevalence for mixed and fully differentiated colonies in *EZH2*$^{-/-}$ compared to control ESC lines. Representative images are shown underneath. Over 100 colonies were scored for each cell line. Scale bars, 100 μm.

(E) Growth curve over 16 days reveals a significant proliferation defect in *EZH2*$^{-/-}$ ESCs compared to control ESCs. Data show mean ± SD; n = 3 biological replicates. Data were compared between *EZH2*$^{-/-}$ ESCs and each control ESC line using one-way ANOVA followed by Bonferroni's multiple comparison test (*$p < 0.05$ for each comparison).

(F) Mitotic index was calculated for each ESC line by dividing the number of H3S10ph-positive cells by the total number of cells within a colony. The analysis was restricted to undifferentiated colonies (determined by OCT4 expression) of similar size in order to control for potential differences in cell state. Over 1,000 cells were scored for each cell line. Data show mean ± SD; n = 3 biological replicates. Data were compared using a one-way ANOVA followed by Bonferroni's multiple comparison test (***$p < 0.0005$). Scale bars, 100 μm.

individual *EZH2*$^{-/-}$ ESCs have upregulated multiple genes associated with a particular cell lineage but rarely show strong signatures derived from several cell lineages (Figure 4B). In particular, *EZH2*$^{-/-}$ ESCs show lineage biases toward endoderm and mesoderm, but not to ectoderm. This response may be constrained by the hESC culture environment due to the activities of FGF and Activin/Nodal signaling within the media, which are known to promote endoderm and mesoderm specification and suppress ectoderm differentiation (Pauklin and Vallier, 2015). Together, these results reveal that the depletion of EZH2 does not cause global EZH2-target gene derepression in all ESCs, as might be predicted from cell population studies. Rather, its loss leads to the mis-regulation of subsets of genes and to the acquisition of lineage-restricted transcriptional programs.

EZH2 Disruption Causes Self-Renewal and Proliferation Defects in hESCs

Undifferentiated *EZH2*-deficient ESCs could be maintained in culture for >50 passages; however, their growth and morphology were severely compromised compared to control lines. *EZH2*$^{-/-}$ colonies were highly variable in appearance with an increased prevalence of flatter cells that are characteristic of spontaneous differentiation (Figure 5A). To compare directly the ability of each hESC line to self-renew, we plated an equal number of

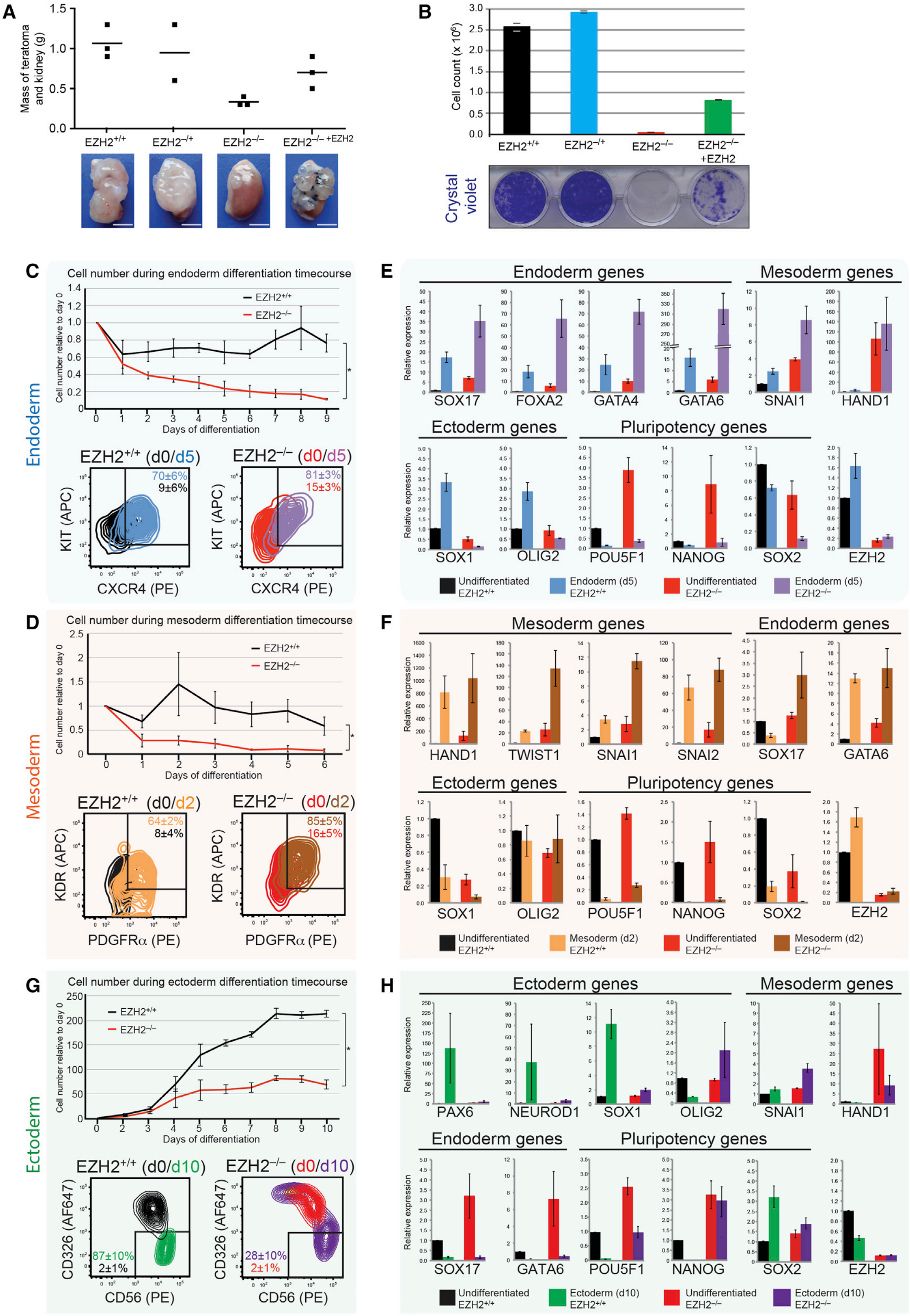

(legend on next page)

SSEA4-positive flow sorted cells at clonal density, and after 7 days we counted the number of colonies that were positive for the undifferentiated hESC marker alkaline phosphatase (AP). We observed an ∼40% reduction in colony number in *EZH2*$^{-/-}$ ESCs compared to control ESCs (Figure 5B).

Diminished colony formation was due to both compromised self-renewal and impaired proliferation. Categorizing *EZH2*$^{-/-}$ ESC colonies based on AP activity patterns revealed an ∼50% reduction in the proportion of undifferentiated colonies in *EZH2*$^{-/-}$ ESCs compared to control ESCs, and an associated increase in the proportion of colonies with a mixed or fully differentiated phenotype (Figure 5C). This was accompanied by a decrease in the proportion of *EZH2*$^{-/-}$ ESC colonies that are entirely OCT4 positive, with an associated increase in the proportion of colonies that are positive for SOX17, a marker of early differentiated cells (Figure 5D). The proportion of undifferentiated and differentiated cells within the *EZH2*$^{-/-}$ ESC cultures was unchanged over passage and was re-established after plating of purified undifferentiated *EZH2*$^{-/-}$ ESCs, further highlighting the unstable nature of these cells.

Cell counts over four passages revealed an ∼50% reduction in cell number in *EZH2*$^{-/-}$ ESCs, revealing that proliferation is significantly reduced in the absence of *EZH2* (Figure 5E). The mitotic index, as determined by the proportion of histone H3 serine 10 phosphorylation (H3S10ph) positive cells, was significantly lower in *EZH2*$^{-/-}$ ESCs compared to control ESCs (Figure 5F). The reduction in mitotic cells within *EZH2*$^{-/-}$ cultures is in agreement with our RNA-seq results, which identify the transcriptional downregulation of genes associated with M-phase (Figure 3B). Of further relevance to this defect is that several negative regulators of the cell cycle, such as *CDKN2A* (encoding p16INK4A and p14ARF) and *CDKN2B* (encoding p15INK4B), were transcriptionally derepressed in *EZH2*-deficient ESCs (Figure S5). These findings are consistent with previous studies in other cell types that have identified a role for EZH2 in controlling the transcription of cell-cycle regulators (Bracken et al., 2003, 2007; Pasini et al., 2004; Sauvageau and Sauvageau, 2010; Varambally et al., 2002). Together, the results demonstrate that *EZH2*-deficient hESCs are strongly compromised in their ability to self-renew and proliferate, thereby identifying a more severe phenotype compared to mouse ESCs that are deficient for *Ezh2* and other PRC2 proteins.

Differentiation Defects in *EZH2*-Deficient hESCs

We next investigated the impact of *EZH2* deletion on the ability of hESCs to differentiate correctly. We injected each hESC line into the kidney capsule of three immunocompromised mice to test for teratoma formation. Control hESC lines produced teratomas consisting of mature cell types derived from all three germ lineages. By contrast, *EZH2*$^{-/-}$ ESCs failed to produce teratomas in two mice and generated a very small mass in one mouse, which consisted of a restricted set of cell types, including immature adipocytes and epithelial cells (Figures 6A, S6A, and S6B). Although the DOX-inducible *EZH2* transgene could partially rescue the *EZH2*$^{-/-}$ phenotype, we noticed that the teratomas formed from the *EZH2*$^{-/-\ +EZH2}$ ESCs were smaller and displayed different morphology compared to the *EZH2*$^{+/+}$ and *EZH2*$^{-/+}$ ESC teratomas (Figures 6A, S6A, and S6B). The difference is likely to be because the cells were not provided with DOX once they were injected in situ, and therefore EZH2 levels would be lost gradually over several days. Further investigation of *EZH2* function in late-stage cell differentiation revealed that very few *EZH2*$^{-/-}$ ESCs survived after 5 days of retinoic-acid-mediated differentiation in vitro, compared to *EZH2*$^{+/+}$ and *EZH2*$^{-/+}$ ESCs (Figure 6B). Restoration of *EZH2* with the DOX-inducible transgene partially rescued the defect, although we noticed that the transgene was silenced at the later stages of cell differentiation, thereby hindering a full rescue. Together, these results lead us to conclude that hESCs require *EZH2* to form late-stage differentiated cell types.

We next studied the early stages of ESC differentiation. PRC2 loss-of-function mutant embryos initiate but fail to complete gastrulation (Faust et al., 1995; O'Carroll et al., 2001; Pasini et al., 2004); however, a detailed examination of PRC2-deficient mouse or human ESC differentiation toward early developmental progenitors using defined conditions has not been reported. To investigate these developmental events, we initiated directed and separate differentiation toward endoderm, mesoderm, and

Figure 6. *EZH2*-Deficient hESCs Can Initiate Differentiation but Are Severely Impaired in Generating Mature Cell Types

(A) *EZH2*$^{-/-}$ ESCs fail to generate teratomas. Mass of teratoma and kidney samples for indicated ESC lines, with images shown underneath (scale bar, 5 mm). Additional images and histology analysis are provided in Figures S6A and S6B.

(B) ESCs were induced to differentiate with retinoic acid for 5 days. Cell counts (upper) and crystal violet stain (lower) reveal that few *EZH2*$^{-/-}$ ESCs remain after 5 days compared to control ESCs. Short bars indicate mean values for the two biological replicates.

(C) *EZH2*-deficient ESCs can generate early endoderm cells. Upper panel shows cell counts over endoderm differentiation time course. Lower panel shows flow cytometry analysis of endoderm markers KIT/CXCR4 in undifferentiated *EZH2*$^{+/+}$ ESCs (black), day 5 endoderm differentiated *EZH2*$^{+/+}$ (blue), undifferentiated *EZH2*$^{-/-}$ ESCs (red), and day 5 endoderm differentiated *EZH2*$^{-/-}$ (purple). Inset numbers show percentage positive cells for each cell population (mean of three biological replicates, with range).

(D) *EZH2*-deficient ESCs can generate early mesoderm cells. Upper panel shows cell counts over 6 days of mesoderm differentiation. Lower panel shows flow cytometry analysis of mesoderm markers KDR/PDGFRα.

(E) RT-qPCR analysis of endoderm, mesoderm, ectoderm and pluripotency genes in undifferentiated (black) and day 5 endoderm differentiated (blue) *EZH2*$^{+/+}$ ESCs, and undifferentiated (red) and day 5 endoderm differentiated (purple) *EZH2*$^{-/-}$ ESCs. Note that *POU5F1* and *NANOG* are also associated with ESC differentiation (Loh and Lim, 2011), which may underlie their elevated expression patterns in *EZH2*$^{-/-}$ ESCs.

(F) qRT-PCR analysis of undifferentiated and day 2 mesoderm differentiated ESCs.

(G) *EZH2*-deficient ESCs can generate early ectoderm cells, but with significantly reduced efficiency compared to EZH2$^{+/+}$ ESCs. Upper panel shows cell counts over 10 days of ectoderm differentiation. Lower panel shows flow cytometry analysis of ectoderm marker CD56 and undifferentiated ESC marker CD326. Note the significantly decreased efficiency of ectoderm differentiation in *EZH2*$^{-/-}$ ESCs compared to *EZH2*$^{+/+}$ ESCs ($p = 0.01$; unpaired two-sided t test).

(H) RT-qPCR analysis of undifferentiated and day 10 ectoderm differentiated ESCs. For all panels, data show mean ± SEM of three biological replicates and were compared using an unpaired two-sided t test (*$p < 0.05$).

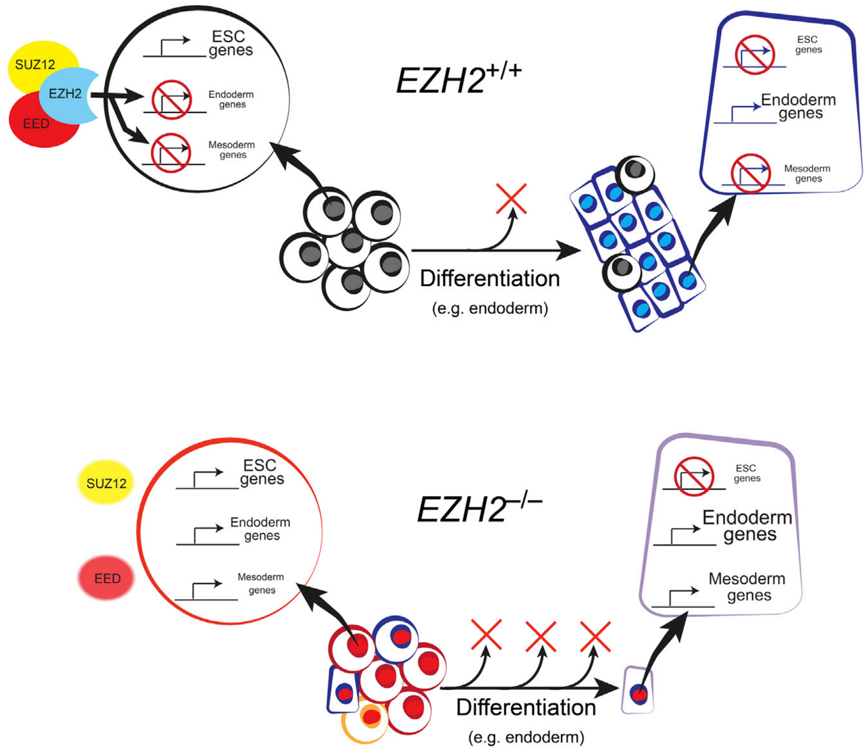

Figure 7. Proposed Model Summarizing the Role of *EZH2* in Regulating Transcriptional Programs and Cell Differentiation in hESCs

EZH2$^{+/+}$, above; and *EZH2*$^{-/-}$, below. *EZH2* deficiency leads to loss of PRC2, transcriptional derepression of developmental regulators and self-renewal defects in hESCs. Substantial cell loss (red crosses) and gene mis-regulation is observed upon differentiation of *EZH2*$^{-/-}$ ESCs (e.g., to endoderm in example shown).

ectoderm progenitors, using defined conditions and lineage-specific markers (Supplemental Experimental Procedures). We observed that cell number declined sharply during endoderm and mesoderm differentiation of *EZH2*$^{-/-}$ ESCs, such that very few cells remained by the end of their differentiation protocols (Figures 6C and 6D). In contrast, cell number was maintained in *EZH2*$^{+/+}$ ESCs during differentiation. We examined the cells at a mid-stage time point as this allowed us to obtain sufficient cells for analysis. Quantitative analysis of lineage-specific, cell-surface markers using flow cytometry showed that *EZH2*$^{-/-}$ ESCs were capable of differentiating into early-stage endoderm (defined as $KIT^{+}/CXCR4^{+}$) (Nostro et al., 2011) and mesoderm (defined as $PDGFR\alpha^{+}/KDR^{+}$) (Kattman et al., 2011) and, surprisingly, formed cell populations at these time points that were more uniform in marker expression than achieved upon differentiation of *EZH2*$^{+/+}$ ESCs (Figures 6C and 6D). To ascertain whether a pre-existing subset of endoderm progenitors were responsible for generating endoderm cells in *EZH2*$^{-/-}$ cultures, we used flow cytometry to separate $KIT^{+}/CXCR4^{+}$ (endoderm primed) and $KIT^{-}/CXCR4^{-}$ (not endoderm primed) *EZH2*$^{-/-}$ populations and subjected the cells to endoderm differentiation. Flow cytometry analysis showed that $KIT^{-}/CXCR4^{-}$ were highly efficient in generating endoderm, thereby demonstrating the ability of *EZH2*-deficient ESCs to respond to appropriate differentiation cues and initiate early-stage differentiation (Figure S6C).

Upon cell differentiation, pluripotency factors *POU5F1*, *NANOG*, and *SOX2* were downregulated to a similar extent in *EZH2*$^{-/-}$ ESCs compared to *EZH2*$^{+/+}$ ESCs (Figures 6E and 6F). This finding is in contrast to PRC2-deficient mouse ESCs, which exhibit a defect in silencing pluripotency networks during cell differentiation (Pasini et al., 2007; Shen et al., 2008). Consistent with our RNA-seq data, genes associated with endoderm and mesoderm differentiation were detected at higher levels at day 0 in *EZH2*$^{-/-}$ ESCs compared to *EZH2*$^{+/+}$ ESCs, and the transcript level of these genes increased further during differentiation, confirming their ability to undergo early-stage differentiation (Figures 6E and 6F). Ectopic expression of lineage-restricted genes occurred during *EZH2*$^{-/-}$ ESC differentiation, suggesting a failure to repress alternate transcriptional programs (Figures 6E and 6F).

EZH2-deficient cells were also able to generate early ectoderm cells (defined as $CD56^{+}/CD326^{-}$) (Gifford et al., 2013); however, cell number during differentiation and the efficiency of differentiation were significantly reduced compared to *EZH2*$^{+/+}$ ESCs (Figure 6G). Mis-regulation of lineage-restricted genes was observed upon ectoderm differentiation, thereby identifying a requirement for EZH2 in regulating appropriate gene expression in the early stages of ectoderm specification (Figure 6H).

Taken together, these results demonstrate that EZH2 is not required for the initial phase of hESC differentiation but is required for the robust generation of mature cell types that are produced in the later stages of differentiation in vitro or in teratoma assays.

DISCUSSION

PcG-proteins are essential regulators of cell-fate decisions and transcriptional programs during the development of several species, including *Drosophila* and the mouse. Here, we show that this important function is also required in humans during the establishment of early developmental cell types that arise upon ESC differentiation (Figure 7). Furthermore, loss of EZH2 function in undifferentiated hESCs led to the transcriptional derepression of ~900 genes including many important developmental regulators, thereby positioning EZH2 as a key factor in controlling the transcriptome of human cell types during early development. Interestingly, not all PRC2-target genes were mis-regulated, suggesting that redundant modes of transcriptional repression are in place, or that additional cues (such as transcription factor binding) are required to fully activate those genes. In addition to

developmental factors, we also detected an increased expression of cell-cycle regulators in the absence of *EZH2*, including *CDKN2A* (encoding p16INK4A and p14ARF) and *CDKN2B* (encoding p15INK4B). Given the close association between cell-cycle control and cell differentiation in hESCs (Pauklin and Vallier, 2013; Gonzales et al., 2015; Pauklin et al., 2016), it is likely that both processes contribute to the phenotype of *EZH2*-deficient cells. For example, an upregulation of p16INK4A would inhibit CDK4/6, which, in turn, would lead to an increase in Activin/Nodal activity (Pauklin and Vallier, 2013). This signaling change would promote the transcriptional programs of endoderm and mesoderm lineages and suppress ectoderm differentiation. We speculate, therefore, that the increased expression of endoderm and mesoderm genes in *EZH2*-deficient hESCs is caused jointly by the removal of repressive H3K27me3 marks, by altered signaling activities that are mediated by cell-cycle machinery, and by the cell-culture environment. Notably, our transcriptional results are consistent with a recent study that reported derepression of a subset of PRC2-target genes in *Ezh2*-deficient mouse epiblast tissue, thereby underscoring the relevance of our observations to pluripotent cells in vivo (Zylicz et al., 2015). Importantly, our analysis of individual cells further revealed that misregulation of genes tended to occur in a coordinated manner within lineage-restricted transcriptional programs, rather than a haphazard derepression of all PRC2-target genes as might be predicted from global cell population analysis. These results suggest the presence of feedback mechanisms that are able to promote or repress alternative cell fates during the early phases of differentiation. An exciting set of future studies will be to model and investigate the mechanisms responsible for this feedback. Our findings also raise broader questions about how cells are committed to a particular lineage during differentiation. Purifying live hESC populations that are in different transcriptional states and challenging the cells to functional assays should begin to unravel the complexities of cell-fate commitment during human development.

Despite extensive conservation in their functions, differences exist between mouse and human ESCs that lack *EZH2*; self-renewal, morphology, and proliferation are seemingly perturbed to a greater extent in human *EZH2*$^{-/-}$ ESCs compared to mouse *Ezh2*$^{-/-}$ ESCs (Shen et al., 2008). One potential explanation is that differences in PRC2 protein stability or function could contribute to the distinct mouse and human ESC phenotypes. For example, Eed and Suz12 levels are unaffected by the loss of Ezh2 in mouse ESCs, potentially due to a partial compensation by Ezh1 (Shen et al., 2008). In contrast, we show here that depletion of EZH2 in hESC results in loss of EED and SUZ12, despite the presence of EZH1. Interestingly, Ezh1 cannot compensate for the absence of Ezh2 during mouse ESC differentiation or embryo gastrulation (O'Carroll et al., 2001; Shen et al., 2008). We speculate there is a context-dependent role for Ezh1 and that the compensatory function diminishes as cells enter the post-implantation phase of development, which could partially explain the apparent inability of EZH1 to fulfill a compensatory role in *EZH2*-deficient hESCs. In addition, Ezh1 is able to repress gene transcription through methylation-independent mechanisms in somatic cells, potentially via chromatin compaction (Margueron et al., 2008). It will therefore be interesting in future studies to more precisely define the functional interplay between EZH1 and EZH2 in early human developmental cell types.

A second potential explanation for the distinct phenotypes is that mouse and human ESCs are known to represent different pluripotent states, with hESCs considered to be primed for differentiation (Nichols and Smith, 2009; Rossant, 2015). The *EZH2*-deficient phenotype may therefore manifest differently depending on cell state, a concept recently proposed for *DNMT1*-depleted ESCs (Liao et al., 2015). It will be important in future studies to test this hypothesis by investigating the role of PRC2 in human "naive" pluripotent cells, which are reported to be more similar to mouse ESCs (Manor et al., 2015). Furthermore, it is interesting to consider that PRC2 may contribute to the balance required for primed-state pluripotency by enabling low-level expression of lineage-specifying developmental regulators while constraining their levels so that they do not overwhelm the maintenance of the undifferentiated state. Given that the ectopic expression of several EZH2 target genes, such as *SOX17* and *GATA6*, can induce the differentiation of hESC (Séguin et al., 2008; Wamaitha et al., 2015), it is plausible that derepression of these and other developmental regulators in the absence of *EZH2* results in a shift toward an increased level of spontaneous differentiation that is observed in *EZH2*-deficient hESCs. Thus, our analysis of PcG function in hESCs should lead to a better understanding of the processes that regulate lineage priming and cell-fate commitment and inform similar events that occur in other species and cell types.

Genome-wide mapping has revealed that dynamic changes in epigenetic marks, including H3K27me3 localization, occur upon hESC differentiation (Gifford et al., 2013; Xie et al., 2013). A prevailing model proposes that this epigenetic reconfiguration is required to coordinate transcriptional programs and provide a memory of cell identity. We have now tested this model, and we show that EZH2 is not required for the initial phase of hESC differentiation as ectoderm, mesoderm, and endoderm germ lineages can form in the absence of EZH2; however, the mutant cells mis-express lineage-specific genes are unstable and are gradually lost over the differentiation time course. Interestingly, and in contrast to PRC2-deficient mouse ESCs (Pasini et al., 2007; Shen et al., 2008), pluripotency genes were downregulated appropriately upon differentiation of *EZH2*-deficient hESCs, suggesting that these genes are silenced by PRC2-independent pathways. The observed differentiation defects and reduction in cell survival are therefore unlikely to be caused by aberrant expression of pluripotency factors, but rather by mis-expression of lineage-specifying genes and cell-cycle regulators. Finally, although the *EZH2*-deficient hESCs were unable to form mature cell types, the rescue of early differentiation defects by conditionally restoring *EZH2* levels should enable the role of PRC2 to be investigated during late-stage in vitro differentiation. As the *EZH2*$^{-/-\ +EZH2}$ cells could not fully recapitulate the parental wild-type cells in the teratoma and the RA-differentiation experiments, alternative conditional systems might be better suited for the investigation of *EZH2* in late-stage cell differentiation. Interestingly, the results from our teratoma experiments suggest that once the *EZH2*$^{-/-}$ ESCs have overcome the initial early-stage differentiation barrier (enabled by residual EZH2), they are able to specialize along certain tissue lineages.

The differences in morphology and tissue composition of the teratomas are presumably, to some extent, a reflection of what differentiation pathways are accessible to *EZH2*-deficient cells. Future studies using alternative conditional strategies and more precise differentiation systems should lead to a better understanding of epigenetic modifiers in the generation of specialized cell types. Artificially controlling *EZH2* levels may also have useful practical applications in producing desired cell types, as has been demonstrated recently to boost production of beta cell progenitors (Xu et al., 2014).

Taken together, our study provides a comprehensive examination of *EZH2* function in hESC pluripotency and differentiation. Of note is that PRC2 mediates the self-renewal and differentiation of adult stem cells and cancer stem cells (Sauvageau and Sauvageau, 2010). Our findings therefore not only reveal the role of epigenetic modifiers and associated histone marks in regulating the genome during early human development, but also establish general principles that can be applicable to stem cells involved in homeostasis and disease.

EXPERIMENTAL PROCEDURES

Cell Culture

hESCs (H9/WA09, obtained from WiCell; WIBR3, kindly provided by Rudolph Jaenisch; FiPS, kindly provided by Austin Smith) were cultured at 37°C in 5% CO_2 in air on CF1 irradiated mouse embryonic fibroblasts (MEFs) in Advanced DMEM containing 20% knockout serum replacement supplemented with 2 mM L-glutamine, 0.1 mM β-mercaptoethanol, 1 × penicillin/streptomycin, 1 × non-essential amino acids (all from Thermo Fisher Scientific) and 4 ng/ml FGF2 (WT-MRC Cambridge Stem Cell Institute). Where indicated, DOX was added at 1 μg/ml. For feeder-free culture, ESCs were transferred onto Vitronectin matrix in TeSR-E8 media (STEMCELL Technologies). Authentication of the hESCs was achieved by confirmation of expression of pluripotency gene and protein markers. Cells were routinely verified as mycoplasma-free using a PCR-based assay. Teratoma formation assays were performed in a designated facility under licenses granted by the UK Home Office. Additional cell-culture materials and methods are detailed in Supplemental Experimental Procedures.

Targeted Deletion of *EZH2*

EZH2 gRNA (CCGCTTCTGCTGTGCCCTTATC) was designed using http://crispr.mit.edu (Hsu et al., 2013). The gRNA sequence was incorporated into the U6 target gRNA expression vector (Mali et al., 2013) and synthesized as a gBlock by Integrated DNA Technologies. The *EZH2* gRNA gBlock was sub-cloned into pCR2.1-TOPO (Thermo Fisher Scientific) and verified by sequencing. hESCs were dissociated into single cells using Accutase (Thermo Fisher Scientific). H9 ESCs (2 million) were nucleofected with 5 μg pCas9_GFP (Addgene plasmid # 44719) and 5 μg *EZH2* gRNA expression vector. After 48 hr, 10,000 GFP-positive single cells were isolated by FACS and seeded onto MEF in a 10-cm tissue culture dish in ESC media supplemented with 10 μM Rho Kinase inhibitor (Cell Guidance Systems) for the first 24 hr. Individual clones were picked and expanded in 24-well plates. Mutations were validated by DNA sequencing of TOPO cloned PCR products. As a check for specificity, ten predicted off-target gRNA sites within genes were tested and verified to contain unmodified sequences.

Plasmid Constructs

To construct PB-TET-EZH2-ires-mCherry plasmid, the *EZH2* coding sequence was amplified using primers EZH2_attb_F and EZH2_attb_R and sub-cloned into PB-TET-ires-mCherry plasmid. To generate $EZH2^{-/-\ +EZH2}$ ESCs, $EZH2^{-/+}$ ESCs were lipofected with 1 μg PB-TET-EZH2-ires-mCherry, 1 μg pCAG-rtTA-Puro, and 2 μg pCyL43 (Wang et al., 2008) followed by selection with 1 μg/ml Puromycin.

To remove PB-TET-EZH2-ires-mCherry, DOX-induced $EZH2^{-/-\ +EZH2}$ ESC were nucleofected with 5 μg pCMV-hyPBase (Yusa et al., 2011) and 1 μg Turbo-GFP (Lonza). After 48 hr, 10,000 GFP/mCherry double-positive single cells were isolated by FACS and seeded onto MEFs in a 10-cm tissue culture dish in ESC media supplemented with 10 μM Rho Kinase inhibitor for the first 24 hr. Individual clones were picked and expanded in 24-well plates. DNA was genotyped using mCherry_Geno and TET-Prom_Geno primers to confirm removal of PB-TET-EZH2-ires-mCherry.

Statistics

For Figure 3D, the data are significantly departed from normality ($p < 0.05$; D'Agostino-Pearson omnibus normality test) and the variance is different between the groups ($p < 0.05$; Brown-Forsythe test); therefore, a non-parametric test was used. For statistical analysis of data within Figures 5 and 6, the scatter of the data lead us to assume that the samples comes from a normally distributed population and that the variability between the groups is about the same; therefore, parametric tests were used.

ACCESSION NUMBERS

The accession number for the sequencing data reported in this paper is GEO: GSE76626.

SUPPLEMENTAL INFORMATION

Supplemental Information includes Supplemental Experimental Procedures and six figures and can be found with this article online at http://dx.doi.org/10.1016/j.celrep.2016.11.032.

AUTHOR CONTRIBUTIONS

A.C. and P.J.R.-G. designed the study, interpreted the results, and wrote the manuscript. A.C. generated all cell lines and performed all experiments. A.J.C. carried out the ectoderm differentiation experiments. N.P.M. generated several ChIP-seq libraries. A.R.S. performed cell-line characterization. T.C. assisted with generating the single-cell RNA-seq libraries. S.A. analyzed single-cell RNA-seq data. P.J.R.-G. conceived and supervised the project, performed experiments, and analyzed data. We consider A.J.C. and N.P.M. to have contributed equally.

ACKNOWLEDGMENTS

We thank members of several Babraham Institute Facilities including Rachael Walker and Arthur Davies in Flow Cytometry, Kristina Tabbada in Sequencing, Anne Segonds-Pichon and Felix Krueger in Bioinformatics, and Simon Walker and Hanneke Okkenhaug in Imaging. We are indebted to William Mansfield at the WT-MRC Stem Cell Institute and Fernando Constantino-Casas and Madeline Fordham at the Department of Veterinary Medicine at the University of Cambridge, for performing the teratoma experiments and histology, respectively. We are grateful to Rudolph Jaenisch at the Whitehead Institute for Biomedical Research for providing WIBR3 cells, Austin Smith at the WT-MRC Stem Cell Institute for providing FiPS cells, Manousos Koutsourakis and Bill Skarnes at the Wellcome Trust Sanger Institute for helpful advice with the use of CRISPR/Cas9 in hESCs, Kiran Musunuru at Harvard University for pCas9_GFP, and Anastasiya Sybirna for constructing the *EZH2* shRNA plasmid. We thank Sarah Elderkin, Wolf Reik, and members of P.J.R.-G.'s group for helpful discussions and comments on the manuscript. P.J.R.-G. is supported by the Wellcome Trust (WT093736) and BBSRC (BBS/E/B/000C0402). A.C. and A.J.C. are supported by MRC DTG Studentships (MR/J003808/1).

Received: July 25, 2016
Revised: October 11, 2016
Accepted: November 9, 2016
Published: December 6, 2016

REFERENCES

Azuara, V., Perry, P., Sauer, S., Spivakov, M., Jørgensen, H.F., John, R.M., Gouti, M., Casanova, M., Warnes, G., Merkenschlager, M., and Fisher, A.G. (2006). Chromatin signatures of pluripotent cell lines. Nat. Cell Biol. *8*, 532–538.

Bernstein, B.E., Mikkelsen, T.S., Xie, X., Kamal, M., Huebert, D.J., Cuff, J., Fry, B., Meissner, A., Wernig, M., Plath, K., et al. (2006). A bivalent chromatin structure marks key developmental genes in embryonic stem cells. Cell *125*, 315–326.

Bock, C., Kiskinis, E., Verstappen, G., Gu, H., Boulting, G., Smith, Z.D., Ziller, M., Croft, G.F., Amoroso, M.W., Oakley, D.H., et al. (2011). Reference Maps of human ES and iPS cell variation enable high-throughput characterization of pluripotent cell lines. Cell *144*, 439–452.

Boyer, L.A., Plath, K., Zeitlinger, J., Brambrink, T., Medeiros, L.A., Lee, T.I., Levine, S.S., Wernig, M., Tajonar, A., Ray, M.K., et al. (2006). Polycomb complexes repress developmental regulators in murine embryonic stem cells. Nature *441*, 349–353.

Bracken, A.P., Pasini, D., Capra, M., Prosperini, E., Colli, E., and Helin, K. (2003). EZH2 is downstream of the pRB-E2F pathway, essential for proliferation and amplified in cancer. EMBO J. *22*, 5323–5335.

Bracken, A.P., Dietrich, N., Pasini, D., Hansen, K.H., and Helin, K. (2006). Genome-wide mapping of Polycomb target genes unravels their roles in cell fate transitions. Genes Dev. *20*, 1123–1136.

Bracken, A.P., Kleine-Kohlbrecher, D., Dietrich, N., Pasini, D., Gargiulo, G., Beekman, C., Theilgaard-Mönch, K., Minucci, S., Porse, B.T., Marine, J.C., et al. (2007). The Polycomb group proteins bind throughout the INK4A-ARF locus and are disassociated in senescent cells. Genes Dev. *21*, 525–530.

Cao, R., and Zhang, Y. (2004). SUZ12 is required for both the histone methyltransferase activity and the silencing function of the EED-EZH2 complex. Mol. Cell *15*, 57–67.

Cao, R., Wang, L., Wang, H., Xia, L., Erdjument-Bromage, H., Tempst, P., Jones, R.S., and Zhang, Y. (2002). Role of histone H3 lysine 27 methylation in Polycomb-group silencing. Science *298*, 1039–1043.

Chamberlain, S.J., Yee, D., and Magnuson, T. (2008). Polycomb repressive complex 2 is dispensable for maintenance of embryonic stem cell pluripotency. Stem Cells *26*, 1496–1505.

Czermin, B., Melfi, R., McCabe, D., Seitz, V., Imhof, A., and Pirrotta, V. (2002). Drosophila enhancer of Zeste/ESC complexes have a histone H3 methyltransferase activity that marks chromosomal Polycomb sites. Cell *111*, 185–196.

Di Croce, L., and Helin, K. (2013). Transcriptional regulation by Polycomb group proteins. Nat. Struct. Mol. Biol. *20*, 1147–1155.

Faust, C., Schumacher, A., Holdener, B., and Magnuson, T. (1995). The eed mutation disrupts anterior mesoderm production in mice. Development *121*, 273–285.

Ferrari, K.J., Scelfo, A., Jammula, S., Cuomo, A., Barozzi, I., Stützer, A., Fischle, W., Bonaldi, T., and Pasini, D. (2014). Polycomb-dependent H3K27me1 and H3K27me2 regulate active transcription and enhancer fidelity. Mol. Cell *53*, 49–62.

Gehani, S.S., Agrawal-Singh, S., Dietrich, N., Christophersen, N.S., Helin, K., and Hansen, K. (2010). Polycomb group protein displacement and gene activation through MSK-dependent H3K27me3S28 phosphorylation. Mol. Cell *39*, 886–900.

Gifford, C.A., Ziller, M.J., Gu, H., Trapnell, C., Donaghey, J., Tsankov, A., Shalek, A.K., Kelley, D.R., Shishkin, A.A., Issner, R., et al. (2013). Transcriptional and epigenetic dynamics during specification of human embryonic stem cells. Cell *153*, 1149–1163.

Gonzales, K.A., Liang, H., Lim, Y.S., Chan, Y.S., Yeo, J.C., Tan, C.P., Gao, B., Le, B., Tan, Z.Y., Low, K.Y., et al. (2015). Deterministic restriction on pluripotent state dissolution by cell-cycle pathways. Cell *162*, 564–579.

Hsu, P.D., Scott, D.A., Weinstein, J.A., Ran, F.A., Konermann, S., Agarwala, V., Li, Y., Fine, E.J., Wu, X., Shalem, O., et al. (2013). DNA targeting specificity of RNA-guided Cas9 nucleases. Nat. Biotechnol. *31*, 827–832.

Jung, H.R., Pasini, D., Helin, K., and Jensen, O.N. (2010). Quantitative mass spectrometry of histones H3.2 and H3.3 in Suz12-deficient mouse embryonic stem cells reveals distinct, dynamic post-translational modifications at Lys-27 and Lys-36. Mol. Cell. Proteomics *9*, 838–850.

Kattman, S.J., Witty, A.D., Gagliardi, M., Dubois, N.C., Niapour, M., Hotta, A., Ellis, J., and Keller, G. (2011). Stage-specific optimization of activin/nodal and BMP signaling promotes cardiac differentiation of mouse and human pluripotent stem cell lines. Cell Stem Cell *8*, 228–240.

Ku, M., Koche, R.P., Rheinbay, E., Mendenhall, E.M., Endoh, M., Mikkelsen, T.S., Presser, A., Nusbaum, C., Xie, X., Chi, A.S., et al. (2008). Genomewide analysis of PRC1 and PRC2 occupancy identifies two classes of bivalent domains. PLoS Genet. *4*, e1000242.

Kuzmichev, A., Nishioka, K., Erdjument-Bromage, H., Tempst, P., and Reinberg, D. (2002). Histone methyltransferase activity associated with a human multiprotein complex containing the Enhancer of Zeste protein. Genes Dev. *16*, 2893–2905.

Lee, T.I., Jenner, R.G., Boyer, L.A., Guenther, M.G., Levine, S.S., Kumar, R.M., Chevalier, B., Johnstone, S.E., Cole, M.F., Isono, K., et al. (2006). Control of developmental regulators by Polycomb in human embryonic stem cells. Cell *125*, 301–313.

Leeb, M., Pasini, D., Novatchkova, M., Jaritz, M., Helin, K., and Wutz, A. (2010). Polycomb complexes act redundantly to repress genomic repeats and genes. Genes Dev. *24*, 265–276.

Liao, J., Karnik, R., Gu, H., Ziller, M.J., Clement, K., Tsankov, A.M., Akopian, V., Gifford, C.A., Donaghey, J., Galonska, C., et al. (2015). Targeted disruption of DNMT1, DNMT3A and DNMT3B in human embryonic stem cells. Nat. Genet. *47*, 469–478.

Loh, K.M., and Lim, B. (2011). A precarious balance: Pluripotency factors as lineage specifiers. Cell Stem Cell *8*, 363–369.

Mali, P., Yang, L., Esvelt, K.M., Aach, J., Guell, M., DiCarlo, J.E., Norville, J.E., and Church, G.M. (2013). RNA-guided human genome engineering via Cas9. Science *339*, 823–826.

Manor, Y.S., Massarwa, R., and Hanna, J.H. (2015). Establishing the human naïve pluripotent state. Curr. Opin. Genet. Dev. *34*, 35–45.

Margueron, R., and Reinberg, D. (2011). The Polycomb complex PRC2 and its mark in life. Nature *469*, 343–349.

Margueron, R., Li, G., Sarma, K., Blais, A., Zavadil, J., Woodcock, C.L., Dynlacht, B.D., and Reinberg, D. (2008). Ezh1 and Ezh2 maintain repressive chromatin through different mechanisms. Mol. Cell *32*, 503–518.

Mikkelsen, T.S., Ku, M., Jaffe, D.B., Issac, B., Lieberman, E., Giannoukos, G., Alvarez, P., Brockman, W., Kim, T.K., Koche, R.P., et al. (2007). Genome-wide maps of chromatin state in pluripotent and lineage-committed cells. Nature *448*, 553–560.

Müller, J., and Verrijzer, P. (2009). Biochemical mechanisms of gene regulation by polycomb group protein complexes. Curr. Opin. Genet. Dev. *19*, 150–158.

Müller, J., Hart, C.M., Francis, N.J., Vargas, M.L., Sengupta, A., Wild, B., Miller, E.L., O'Connor, M.B., Kingston, R.E., and Simon, J.A. (2002). Histone methyltransferase activity of a Drosophila Polycomb group repressor complex. Cell *111*, 197–208.

Nekrasov, M., Wild, B., and Müller, J. (2005). Nucleosome binding and histone methyltransferase activity of Drosophila PRC2. EMBO Rep. *6*, 348–353.

Nichols, J., and Smith, A. (2009). Naive and primed pluripotent states. Cell Stem Cell *4*, 487–492.

Nostro, M.C., Sarangi, F., Ogawa, S., Holtzinger, A., Corneo, B., Li, X., Micallef, S.J., Park, I.H., Basford, C., Wheeler, M.B., et al. (2011). Stage-specific signaling through TGFβ family members and WNT regulates patterning and pancreatic specification of human pluripotent stem cells. Development *138*, 861–871.

O'Carroll, D., Erhardt, S., Pagani, M., Barton, S.C., Surani, M.A., and Jenuwein, T. (2001). The polycomb-group gene Ezh2 is required for early mouse development. Mol. Cell. Biol. *21*, 4330–4336.

Pan, G., Tian, S., Nie, J., Yang, C., Ruotti, V., Wei, H., Jonsdottir, G.A., Stewart, R., and Thomson, J.A. (2007). Whole-genome analysis of histone H3 lysine 4

and lysine 27 methylation in human embryonic stem cells. Cell Stem Cell *1*, 299–312.

Pasini, D., and Di Croce, L. (2016). Emerging roles for Polycomb proteins in cancer. Curr. Opin. Genet. Dev. *36*, 50–58.

Pasini, D., Bracken, A.P., Jensen, M.R., Lazzerini Denchi, E., and Helin, K. (2004). Suz12 is essential for mouse development and for EZH2 histone methyltransferase activity. EMBO J. *23*, 4061–4071.

Pasini, D., Bracken, A.P., Hansen, J.B., Capillo, M., and Helin, K. (2007). The polycomb group protein Suz12 is required for embryonic stem cell differentiation. Mol. Cell. Biol. *27*, 3769–3779.

Pasini, D., Malatesta, M., Jung, H.R., Walfridsson, J., Willer, A., Olsson, L., Skotte, J., Wutz, A., Porse, B., Jensen, O.N., and Helin, K. (2010). Characterization of an antagonistic switch between histone H3 lysine 27 methylation and acetylation in the transcriptional regulation of Polycomb group target genes. Nucleic Acids Res. *38*, 4958–4969.

Pauklin, S., and Vallier, L. (2013). The cell-cycle state of stem cells determines cell fate propensity. Cell *155*, 135–147.

Pauklin, S., and Vallier, L. (2015). Activin/Nodal signalling in stem cells. Development *142*, 607–619.

Pauklin, S., Madrigal, P., Bertero, A., and Vallier, L. (2016). Initiation of stem cell differentiation involves cell cycle-dependent regulation of developmental genes by Cyclin D. Genes Dev. *30*, 421–433.

Pietersen, A.M., and van Lohuizen, M. (2008). Stem cell regulation by polycomb repressors: Postponing commitment. Curr. Opin. Cell Biol. *20*, 201–207.

Riising, E.M., Comet, I., Leblanc, B., Wu, X., Johansen, J.V., and Helin, K. (2014). Gene silencing triggers polycomb repressive complex 2 recruitment to CpG islands genome wide. Mol. Cell *55*, 347–360.

Rossant, J. (2015). Mouse and human blastocyst-derived stem cells: Vive les differences. Development *142*, 9–12.

Sauvageau, M., and Sauvageau, G. (2010). Polycomb group proteins: Multi-faceted regulators of somatic stem cells and cancer. Cell Stem Cell *7*, 299–313.

Schoenfelder, S., Sugar, R., Dimond, A., Javierre, B.M., Armstrong, H., Mifsud, B., Dimitrova, E., Matheson, L., Tavares-Cadete, F., Furlan-Magaril, M., et al. (2015). Polycomb repressive complex PRC1 spatially constrains the mouse embryonic stem cell genome. Nat. Genet. *47*, 1179–1186.

Schuettengruber, B., and Cavalli, G. (2009). Recruitment of polycomb group complexes and their role in the dynamic regulation of cell fate choice. Development *136*, 3531–3542.

Séguin, C.A., Draper, J.S., Nagy, A., and Rossant, J. (2008). Establishment of endoderm progenitors by SOX transcription factor expression in human embryonic stem cells. Cell Stem Cell *3*, 182–195.

Shen, X., Liu, Y., Hsu, Y.J., Fujiwara, Y., Kim, J., Mao, X., Yuan, G.C., and Orkin, S.H. (2008). EZH1 mediates methylation on histone H3 lysine 27 and complements EZH2 in maintaining stem cell identity and executing pluripotency. Mol. Cell *32*, 491–502.

Surface, L.E., Thornton, S.R., and Boyer, L.A. (2010). Polycomb group proteins set the stage for early lineage commitment. Cell Stem Cell *7*, 288–298.

Tie, F., Stratton, C.A., Kurzhals, R.L., and Harte, P.J. (2007). The N terminus of Drosophila ESC binds directly to histone H3 and is required for E(Z)-dependent trimethylation of H3 lysine 27. Mol. Cell. Biol. *27*, 2014–2026.

Varambally, S., Dhanasekaran, S.M., Zhou, M., Barrette, T.R., Kumar-Sinha, C., Sanda, M.G., Ghosh, D., Pienta, K.J., Sewalt, R.G., Otte, A.P., et al. (2002). The polycomb group protein EZH2 is involved in progression of prostate cancer. Nature *419*, 624–629.

Wamaitha, S.E., del Valle, I., Cho, L.T., Wei, Y., Fogarty, N.M., Blakeley, P., Sherwood, R.I., Ji, H., and Niakan, K.K. (2015). Gata6 potently initiates reprograming of pluripotent and differentiated cells to extraembryonic endoderm stem cells. Genes Dev. *29*, 1239–1255.

Wang, H., Wang, L., Erdjument-Bromage, H., Vidal, M., Tempst, P., Jones, R.S., and Zhang, Y. (2004). Role of histone H2A ubiquitination in Polycomb silencing. Nature *431*, 873–878.

Wang, W., Lin, C., Lu, D., Ning, Z., Cox, T., Melvin, D., Wang, X., Bradley, A., and Liu, P. (2008). Chromosomal transposition of PiggyBac in mouse embryonic stem cells. Proc. Natl. Acad. Sci. USA *105*, 9290–9295.

Xie, W., Schultz, M.D., Lister, R., Hou, Z., Rajagopal, N., Ray, P., Whitaker, J.W., Tian, S., Hawkins, R.D., Leung, D., et al. (2013). Epigenomic analysis of multilineage differentiation of human embryonic stem cells. Cell *153*, 1134–1148.

Xu, C.R., Li, L.C., Donahue, G., Ying, L., Zhang, Y.W., Gadue, P., and Zaret, K.S. (2014). Dynamics of genomic H3K27me3 domains and role of EZH2 during pancreatic endocrine specification. EMBO J. *33*, 2157–2170.

Yusa, K., Zhou, L., Li, M.A., Bradley, A., and Craig, N.L. (2011). A hyperactive piggyBac transposase for mammalian applications. Proc. Natl. Acad. Sci. USA *108*, 1531–1536.

Zhao, X.D., Han, X., Chew, J.L., Liu, J., Chiu, K.P., Choo, A., Orlov, Y.L., Sung, W.K., Shahab, A., Kuznetsov, V.A., et al. (2007). Whole-genome mapping of histone H3 Lys4 and 27 trimethylations reveals distinct genomic compartments in human embryonic stem cells. Cell Stem Cell *1*, 286–298.

Zylicz, J.J., Dietmann, S., Günesdogan, U., Hackett, J.A., Cougot, D., Lee, C., and Surani, M.A. (2015). Chromatin dynamics and the role of G9a in gene regulation and enhancer silencing during early mouse development. eLife *4*, 4.

Cell Reports, Volume *17*

Supplemental Information

Deletion of the Polycomb-Group Protein EZH2 Leads to Compromised Self-Renewal and Differentiation Defects in Human Embryonic Stem Cells

Adam Collinson, Amanda J. Collier, Natasha P. Morgan, Arnold R. Sienerth, Tamir Chandra, Simon Andrews, and Peter J. Rugg-Gunn

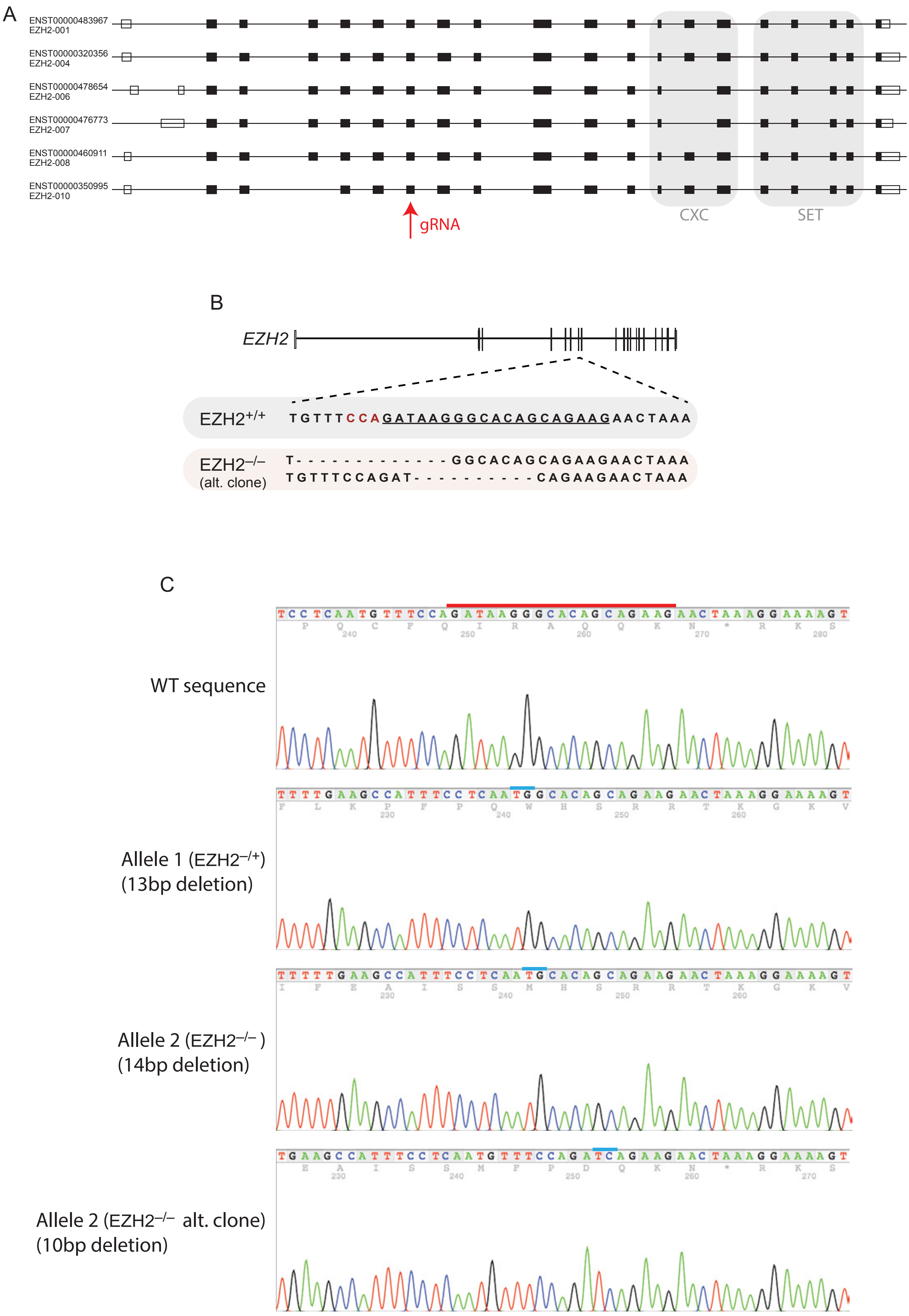
A
ENST00000483967
EZH2-001
ENST00000320356
EZH2-004
ENST00000478654
EZH2-006
ENST00000476773
EZH2-007
ENST00000460911
EZH2-008
ENST00000350995
EZH2-010
gRNA
CXC
SET
B
EZH2
EZH2+/+
TGTTTCCAGATAAGGGCACAGCAGAAGAACTAAA
EZH2–/–
(alt. clone)
T-------------GGCACAGCAGAAGAACTAAA
TGTTTCCAGAT----------CAGAAGAACTAAA
C
WT sequence
Allele 1 (EZH2–/+)
(13bp deletion)
Allele 2 (EZH2–/–)
(14bp deletion)
Allele 2 (EZH2–/– alt. clone)
(10bp deletion)

Figure S1, related to Figure 1.

Targeted deletion of *EZH2* in human ESC.

(A) Exon structure of *EZH2* isoforms (Ensembl) and the location of gRNA sequence.

(B) DNA sequence of the deletions in an alternative *EZH2*$^{-/-}$ ESC line is shown for both alleles. Mutation causes frameshift and premature stop codon. The gRNA sequence is underlined and protospacer adjacent motif highlighted in red.

(C) Sanger DNA sequencing chromatograms for *EZH2*$^{+/+}$, *EZH2*$^{-/+}$ and two *EZH2*$^{-/-}$ ESC lines. The red line indicates the position of the gRNA sequence. The blue lines indicate the position of the deletion.

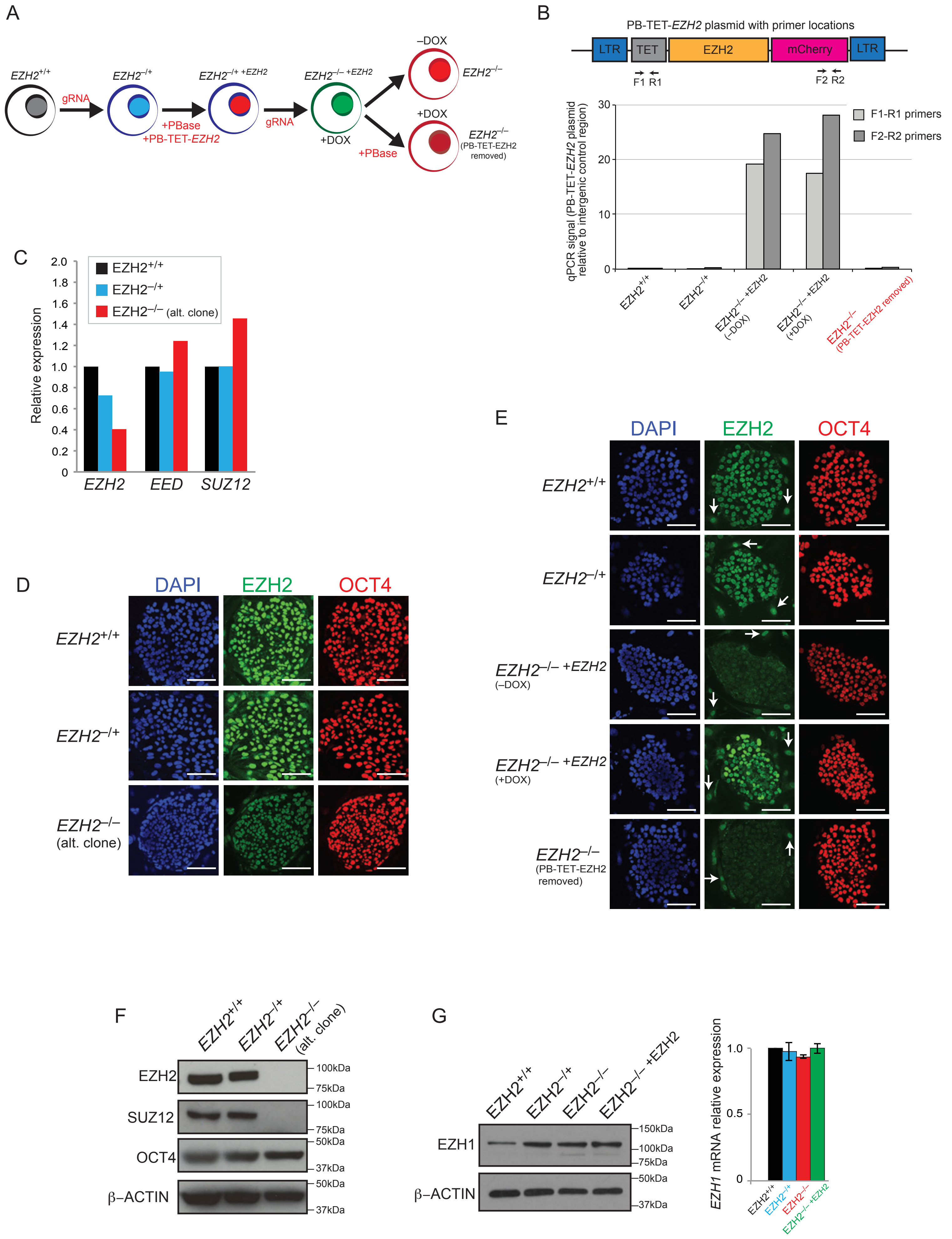
A
EZH2+/+
gRNA
EZH2−/+
+PBase
+PB-TET-EZH2
EZH2−/+ +EZH2
gRNA
EZH2−/− +EZH2
+DOX
−DOX
EZH2−/−
+DOX
+PBase
EZH2−/−
(PB-TET-EZH2 removed)
B
PB-TET-EZH2 plasmid with primer locations
LTR
TET
EZH2
mCherry
LTR
F1 R1
F2 R2
qPCR signal (PB-TET-EZH2 plasmid relative to intergenic control region)
F1-R1 primers
F2-R2 primers
EZH2+/+
EZH2−/+
EZH2−/− +EZH2 (−DOX)
EZH2−/− +EZH2 (+DOX)
EZH2−/− (PB-TET-EZH2 removed)
C
Relative expression
EZH2+/+
EZH2−/+
EZH2−/− (alt. clone)
EZH2
EED
SUZ12
D
DAPI
EZH2
OCT4
EZH2+/+
EZH2−/+
EZH2−/− (alt. clone)
E
DAPI
EZH2
OCT4
EZH2+/+
EZH2−/+
EZH2−/− +EZH2 (−DOX)
EZH2−/− +EZH2 (+DOX)
EZH2−/− (PB-TET-EZH2 removed)
F
EZH2+/+
EZH2−/+
EZH2−/− (alt. clone)
EZH2
SUZ12
OCT4
β−ACTIN
100kDa
75kDa
50kDa
37kDa
G
EZH2+/+
EZH2−/+
EZH2−/−
EZH2−/− +EZH2
EZH1
β−ACTIN
150kDa
100kDa
75kDa
50kDa
37kDa
EZH1 mRNA relative expression
EZH2+/+
EZH2−/+
EZH2−/−
EZH2−/− +EZH2

Figure S2, related to Figure 1.

Additional characterisation of *EZH2*-deficient human ESC.

(A) Overview of targeting strategy including generation of *EZH2*-deficient ESC containing a DOX-inducible *EZH2* transgene (PB-TET-*EZH2*) and after transient expression of piggyBac transposase (PBase) to excise all copies of the transgene.

(B) qPCR on genomic DNA extracted from indicated ESC lines using two primer pairs that are specific for the PB-TET-*EZH2* transgene are shown relative to one primer pair that amplifies a control region that is not within the transgene. These data demonstrate that transient PBase expression excised the transgene.

(C) RT-qPCR analysis of PRC2 components *EZH2*, *EED* and *SUZ12* in $EZH2^{+/+}$, $EZH2^{-/+}$ and an alternative $EZH2^{-/-}$ ESC line.

(D) Immunofluorescent microscopy confirms a strong reduction in EZH2 levels in an alternative $EZH2^{-/-}$ ESC line compared to control ESC. The antibody was raised against a C-terminal epitope of EZH2. OCT4 expression indicates undifferentiated cells within an ESC colony. Scale bars, 100μm.

(E) Immunofluorescent microscopy reveals a strong reduction in EZH2 levels in $EZH2^{-/-}$ ESC in the absence of DOX and also after transgene excision (in the presence of DOX). The antibody was raised against an N-terminal epitope of EZH2. OCT4 expression indicates undifferentiated cells within an ESC colony. Arrows point to MEF. Scale bars, 100μm.

(F) EZH2 and SUZ12 are undetectable in an alternative $EZH2^{-/-}$ ESC line by Western blot analysis. β-ACTIN is loading control. Mass in kDa.

(G) EZH1 is expressed in $EZH2^{-/-}$ ESC by Western blot analysis (left panel) and by qRT-PCR analysis (right panel), and levels are unaffected by the loss of *EZH2*. β-ACTIN is loading control. Mass in kDa. qRT-PCR data show mean ± s.d.; n=3 biological replicates.

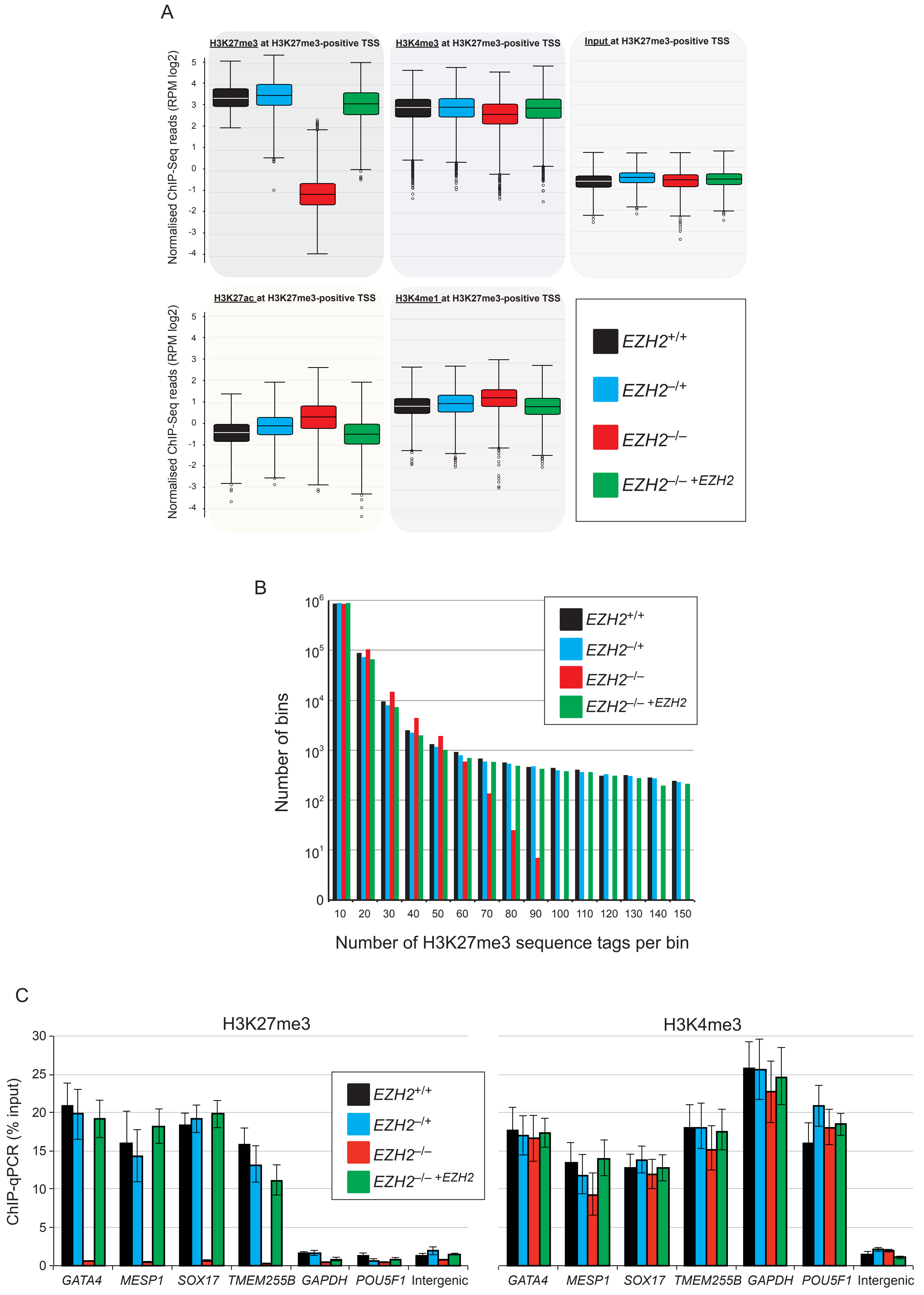
A
H3K27me3 at H3K27me3-positive TSS
H3K4me3 at H3K27me3-positive TSS
Input at H3K27me3-positive TSS
H3K27ac at H3K27me3-positive TSS
H3K4me1 at H3K27me3-positive TSS
Normalised ChIP-Seq reads (RPM log2)
EZH2+/+
EZH2−/+
EZH2−/−
EZH2−/− +EZH2
B
Number of bins
Number of H3K27me3 sequence tags per bin
C
H3K27me3
H3K4me3
ChIP-qPCR (% input)
GATA4
MESP1
SOX17
TMEM255B
GAPDH
POU5F1
Intergenic

Figure S3, related to Figure 2.

Disruption of *EZH2* causes loss of promoter and genome-wide H3K27me3 in human ESC.

(A) Boxplot analysis of normalised ChIP-Seq reads for H3K27me3-positive (in EZH2$^{+/+}$ ESC) gene promoters (±2.5kb TSS; n=2081) in the indicated ESC lines. Panels show four different histone modifications, plus input sample. Promoters show a substantial reduction of H3K27me3 and moderate increase in H3K27ac in *EZH2*$^{-/-}$ ESC compared to control ESC.

(B) Genome-wide reduction in H3K27me3 levels in *EZH2*-deficient ESC. 150Mb of Chromosome 5 (arbitrarily chosen) was divided into 300bp bins with 150bp step size, and the number of non-duplicated reads per bin was quantified and globally normalised to the data set with the highest coverage. A similar number of bins have a low sequence count per bin in all four ESC lines; however EZH2$^{-/-}$ ESC have very few bins with a high sequence count per bin.

(C) Additional validation provided by qPCR analysis of ChIP DNA for several gene promoter regions in *EZH2*-deficient and control ESC lines. Gene promoters include *GATA4*, *MESP1*, *SOX17* and *TMEM255B*, which are marked by H327me3 and H3K4me3 in wild-type ESC; *GAPDH* and *POU5F1*, which are marked by H3K4me3 only in ESC; and a negative control intergenic region that is not marked by either histone modification. Consistent with the ChIP-Seq data, the qPCR analysis reveals that H3K27me3 signals are reduced to background levels in *EZH2*$^{-/-}$ ESC, compared to control ESC, whereas H3K4me3 signals are largely unaffected. Data show mean ± s.d.; n=3 biological replicates.

Collinson_Fig.S4

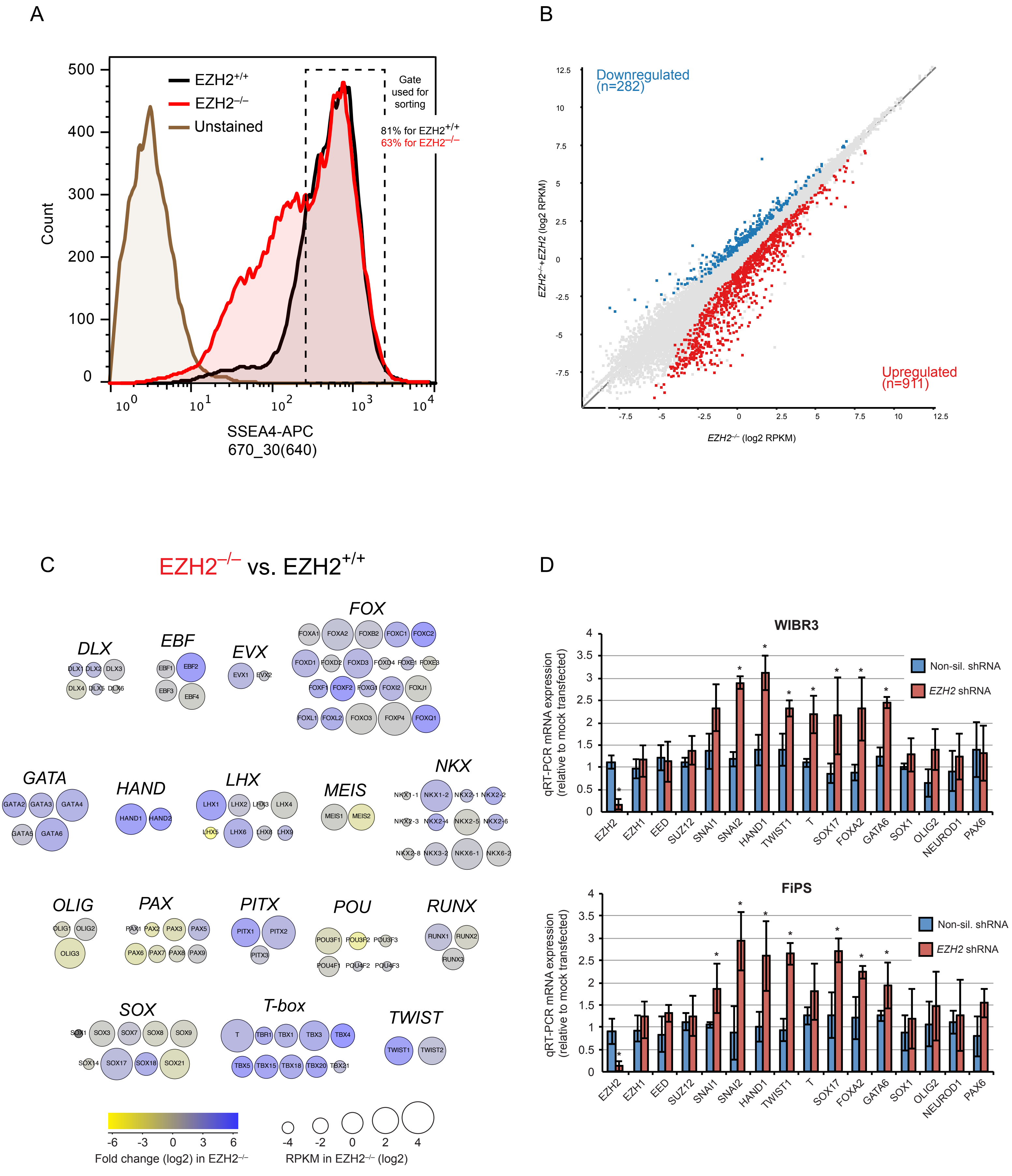

Figure S4, related to Figure 3.

Genes encoding key developmental regulators are transcriptionally derepressed in *EZH2*-deficient hESC.

(A) Flow cytometry histogram of SSEA4 levels and sorting strategy used for $EZH2^{+/+}$ and $EZH2^{-/-}$ ESC. Numbers show percentage positive cells within sorting gate for each cell line.

(B) Scatter plot of RNA-Seq transcript levels of all genes in $EZH2^{-/-}$ ESC versus $EZH2^{-/- +EZH2}$ ESC. Upregulated and downregulated genes are highlighted.

(C) A subset of direct EZH2 target genes is grouped into families. The colour of each circle represents the log2 fold change in $EZH2^{-/-}$ ESC relative to $EZH2^{+/+}$ ESC. The size of each circle represents the expression value of the gene in $EZH2^{-/-}$ ESC.

(D) qRT-PCR analysis of WIBR3 (upper) and FiPS (lower) human pluripotent stem cells lines 72h after transfection with shRNA targeting *EZH2*. Non-silencing shRNA provides a negative control. All values are displayed relative to mock-transfected cells. *EZH2* mRNA was depleted to ~15% levels compared to mock-transfected and non-silencing samples. *EZH1*, *EED* and *SUZ12* mRNA levels are unaffected. Several genes encoding developmental regulators are transcriptionally derepressed upon *EZH2* knockdown, particularly those associated with mesoderm (*SNAI1*, *SNAI2*, *HAND1*, *TWIST1*, T) and endoderm (*SOX17*, *FOXA2*, *GATA6*) differentiation. Genes associated with ectoderm (*SOX1*, *OLIG2*, *NEUROD1*, *PAX6*) differentiation are unchanged. Data show mean ± s.d.; n=3 biological replicates, and the *EZH2* shRNA samples were compared against the non-silencing shRNA samples using an unpaired two-sided t-test (*, $p<0.05$).

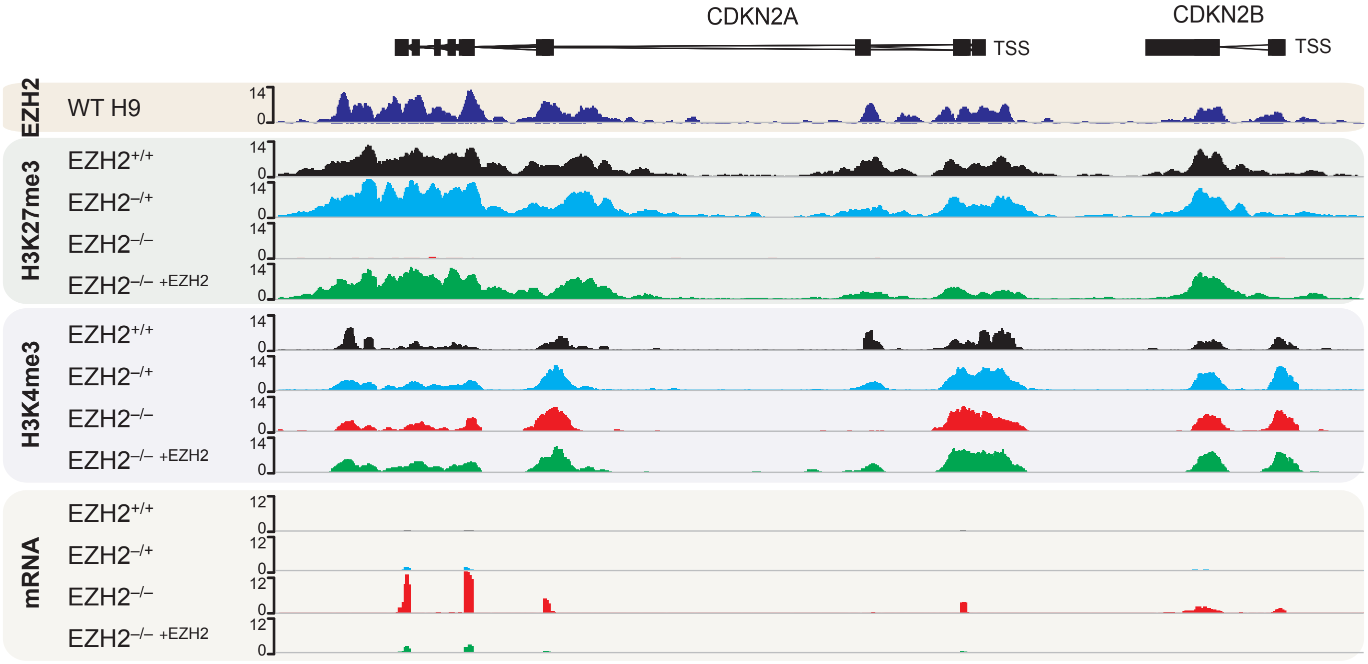
CDKN2A
TSS
CDKN2B
TSS
EZH2
WT H9
H3K27me3
EZH2+/+
EZH2−/+
EZH2−/−
EZH2−/− +EZH2
H3K4me3
EZH2+/+
EZH2−/+
EZH2−/−
EZH2−/− +EZH2
mRNA
EZH2+/+
EZH2−/+
EZH2−/−
EZH2−/− +EZH2
14
0
12
0

Figure S5, related to Figure 5.

Genes encoding key cell cycle regulators are transcriptionally derepressed in *EZH2*-deficient human ESC.

ChIP-Seq and mRNA-Seq tracks of two genes encoding key cell cycle regulators illustrate the association between loss of H3K27me3 and transcriptional upregulation in $EZH2^{-/-}$ ESC compared to control ESC. The transcriptional upregulation of *CDKN2A* and *CDKN2B* could partially underlie the proliferation impairment in $EZH2^{-/-}$ ESC.

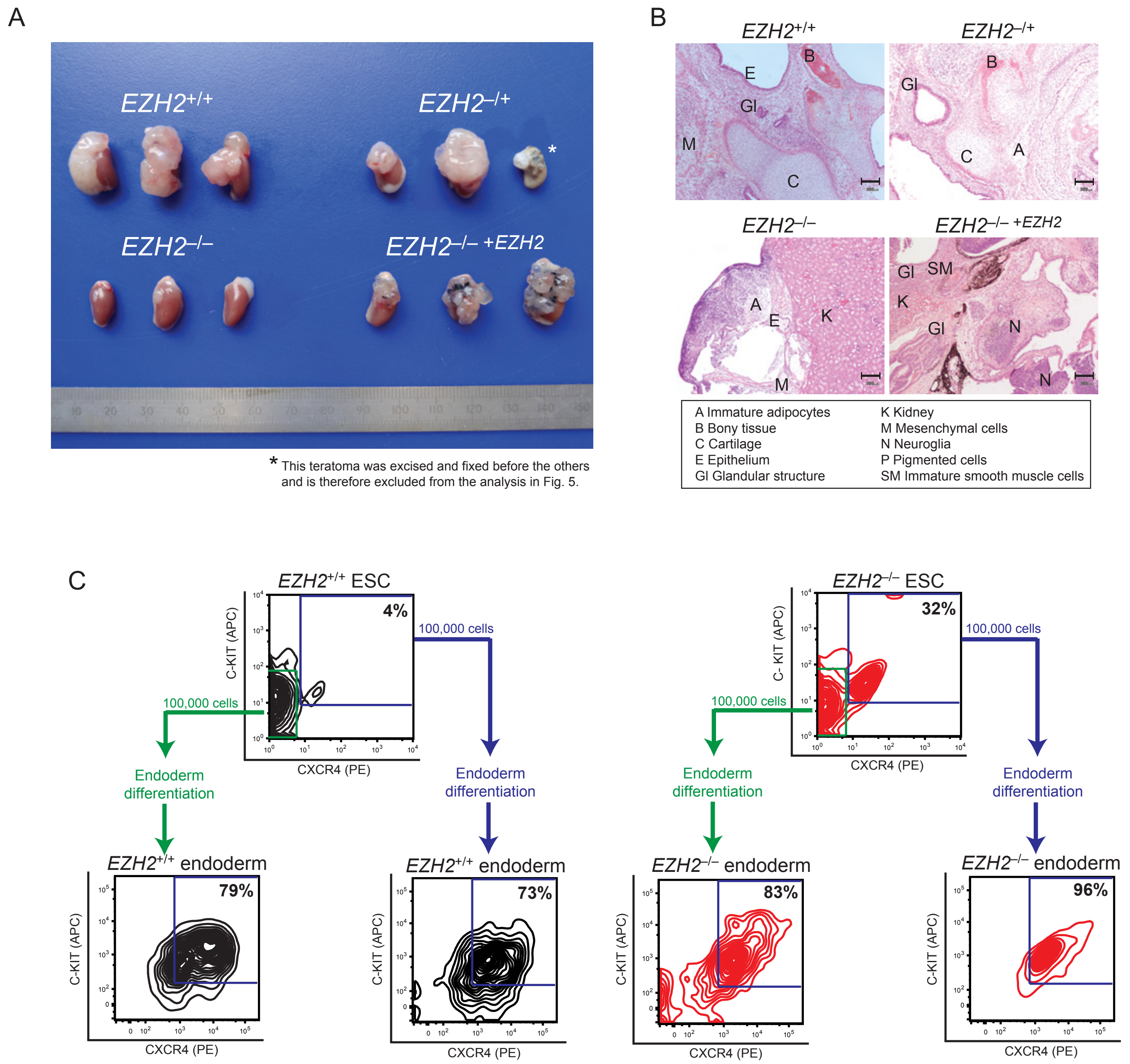
A
EZH2+/+
EZH2−/+
*
EZH2−/−
EZH2−/− +EZH2
* This teratoma was excised and fixed before the others and is therefore excluded from the analysis in Fig. 5.
B
EZH2+/+
EZH2−/+
EZH2−/−
EZH2−/− +EZH2
A Immature adipocytes
B Bony tissue
C Cartilage
E Epithelium
Gl Glandular structure
K Kidney
M Mesenchymal cells
N Neuroglia
P Pigmented cells
SM Immature smooth muscle cells
C
EZH2+/+ ESC
4%
100,000 cells
C-KIT (APC)
CXCR4 (PE)
Endoderm differentiation
EZH2+/+ endoderm
79%
73%
EZH2−/− ESC
32%
EZH2−/− endoderm
83%
96%

Figure S6, related to Figure 6.

***EZH2*-deficient human ESC can initiate differentiation, but cannot generate mature cell types.**

(A) *EZH2*$^{-/-}$ ESC fail to generate teratomas. Three teratomas were analysed for each ESC line. The ruler underneath provides a size reference.

(B) Representative images of haematoxylin and eosin staining of teratomas. Note that control ESC formed large teratomas in all experiments, whereas EZH2$^{-/-}$ ESC generated a very small mass in only one kidney. The presence of various tissue types are indicated with key shown underneath. *EZH2*$^{-/-}$ ESC are unable to form mature cell types that are abundant in teratomas derived from control ESC. Scale bars, 300µm.

(C) Flow cytometry analysis confirms that C-KIT/CXCR4 negative *EZH2*$^{-/-}$ ESC are able to form C-KIT/CXCR4 positive endoderm progenitors after five days of differentiation. This experiment confirms that endoderm cells generated from a bulk population of *EZH2*$^{-/-}$ ESC are not arising entirely from pre-existing endoderm-primed cells.

Supplemental Experimental Procedures

Retinoic Acid differentiation

ESC were seeded onto Vitronectin matrix in TeSR-E8 media in 12-well plates. After 48h, 5μM of retinoic acid (Sigma-Aldrich) was added to each well in N2B27 media (50% DMEM-F12 (Thermo Fisher Scientific), 50% Neurobasal (Thermo Fisher Scientific) supplemented with 0.5X B-27 Supplement (Thermo Fisher Scientific), 0.5X N2 Supplement (Thermo Fisher Scientific), 2mM L-Glutamine, 0.1mM β-mercaptoethanol and 1x Penicillin/Streptomycin. Media was changed every 24h and cells processed on day 5 after induction.

Endoderm differentiation

Endoderm differentiation was achieved following the protocol by Nostro and colleagues (Nostro et al., 2011) with minor modifications. Briefly, three days after passage, ESC were washed once with RPMI-1640 (Thermo Fisher Scientific) and incubated overnight in 5%CO_2;5%O_2 in Endoderm Aggregation Media (RPMI-1640 supplemented with 2mM L-Glutamine (Thermo Fisher Scientific), 450nM MTG (Sigma), 1X Penicillin/Streptomycin (Thermo Fisher Scientific), 50μg/ml Ascorbic Acid (Sigma), 0.25ng/ml BMP-4 (R&D Systems), 5ng/ml FGF-2 (WT–MRC Cambridge Stem Cell Institute), 100ng/ml activin A (WT–MRC Cambridge Stem Cell Institute) and 10ng/ml VEGF (R&D Systems)). The following day (designated day 0) the media was replaced with endoderm induction media (RPMI-1640 supplemented with 2mM L-Glutamine (Thermo Fisher Scientific), 450nM MTG (Sigma), 1X Penicillin/Streptomycin (Thermo Fisher Scientific), 50μg/ml Ascorbic Acid (Sigma), 100ng/ml activin A (WT–MRC Cambridge Stem Cell Institute), 25ng/ml Wnt3a (R&D Systems) and 0.2-2% FBS (Sigma). Media was changed every 48h and cells were typically harvested on day 5.

Mesoderm differentiation

Mesoderm differentiation was achieved following the protocol by Kennedy and colleagues (Kennedy et al., 2007) with minor modifications. Briefly, ESC colonies were lifted away from MEF using 1mg/ml Collagenase IV, washed, and the colonies triturated to obtain small clusters. The clusters were cultured overnight as embryoid bodies (EB) in a non-tissue culture treated Petri dish on a rocker at 30 rpm at 5%CO_2;5%O_2 in 12ml mesoderm aggregation media: STEMPRO34 (Thermo Fisher Scientific) supplemented with 2mM L-Glutamine (Thermo Fisher Scientific), 450nM MTG (Sigma), 1X Penicillin/Streptomycin (Thermo Fisher Scientific), 50μg/ml Ascorbic Acid (Sigma), 150mg/ml Apo-Transferrin (Sigma), 2ng/ml BMP-4 (R&D Systems). The following day, half of the media was discarded and replaced with 6ml fresh mesoderm aggregation media supplemented with 10ng/ml FGF-2. Eighteen hours later (designed as day 0), EBs were collected by settling and re-suspended in mesoderm induction media (STEMPRO 34 supplemented with 2mM L-Glutamine, 450nM MTG, 1X Penicillin/Streptomycin, 50 μg/ml Ascorbic Acid, 150mg/ml Apo-Transferrin, 60ng/ml BMP-4, 10ng/ml activinA, 5ng/ml FGF-2). Cells were typically harvested on day 2.

Ectoderm differentiation

Ectoderm differentiation was achieved following the protocol by Lee and colleagues (Lee et al., 2010). Briefly, ESC were seeded at a density of 10,000 cells per cm^2 on Matrigel in mTeSR1 media (StemCell Technologies) and incubated in 5%CO_2 in air (designated as day 0). Differentiation was initiated on day 2 by replacing the media with KSR media (Advanced DMEM with 15% KSR, 2mM L-Glutamine, 1X Penicillin/Streptomycin (all from Thermo Fisher Scientific), 0.1mM β-mercaptoethanol (Sigma-Aldrich), supplemented with 10μM SB431542 and 500ng/ml Noggin. On day 4, media was replaced with 75% KSR media / 25% N2 media (Neurobasal supplemented with N2, B27 (without RA) and 2mM L-Glutamine; all from Thermo Fisher Scientific) containing 10μM SB431542 and 500ng/ml Noggin. On day 6, media was replaced with 50% KSR media / 50% N2 media containing 10μM SB431542 and 500ng/ml Noggin, and on day 8, media was replaced with 25% KSR media / 75% N2 media containing 10μM SB431542 and 500ng/ml Noggin. Cells were typically harvested on day 10.

Teratoma formation

Teratoma formation assays were provided as a service by the WT–MRC Cambridge Stem Cell Institute in a designated facility under licenses granted by the UK Home Office. HESC were injected beneath the kidney capsule of immune-deficient mice (NOD/SCID) as 1 million cells per animal. Four hESC lines were tested, with one hESC line injected into one kidney per animal and a total of three animals used for each hESC line. Mice were male, 12-weeks old and obtained from Charles River. Randomisation, but not experimental 'blinding' to sample identity, was used for these studies. Teratomas were excised after 8 weeks, except for one teratoma from an *EZH2*$^{-/+}$ hESC line that was excised after 6 weeks due to animal health. Samples were fixed, embedded, sectioned, stained with hematoxylin and eosin and examined as a service by the Histopathology Facility at the University of Cambridge.

EZH2 shRNA knockdown experiments

EZH2 shRNA (clone ID: V3LHS_412598) and non-silencing shRNA were purchased from Dharmacon and cloned into Neomycin EF1α mCherry expression plasmid (Golding et al., 2010) using *MluI* and *XhoI*. WIBR3 and FiPS cells were dissociated into single cells using Accutase (Thermo Fisher Scientific). Cells (2 million) were nucleofected with 10μg

Neomycin EF1α *EZH2* shRNA mCherry, 10μg Neomycin EF1α non-silencing shRNA mCherry, or mock transfected. Cells were plated in ESC media, supplemented for the first 24h with 10μM Rho Kinase inhibitor (Sigma-Aldrich). 72h after nucleofection, mCherry-positive cells were sorted by FACS directly into Trizol LS.

Flow cytometry
Cells were washed once with PBS and incubated with 0.05% Trypsin (Thermo Fisher Scientific) for 5 minutes at 37°C. Media containing 5% FBS was added to inactivate the trypsin and samples triturated gently to form a single cell suspension. Cells were washed once with 2% FBS in PBS and collected by centrifugation at 1000 rpm for 5 minutes. Primary antibody (diluted in 2% FBS) was applied for 1 hr at 4°C in the dark, washed once more, and suspended in 400μl 2% FBS, 1ug/ml DAPI. Cells were analysed on a LSRII or Fortessa V (BD) or sorted on an Influx or Aria III (BD) at the Babraham Institute Flow Cytometry Facility.

Alkaline Phosphatase staining
SSEA4-positive live ESC were obtained by FACS and 6000 cells were seeded in ESC media onto MEF-coated 12-well plates or in mTeSR-E8 media onto Vitronectin-coated 12-well plates. Media was supplemented with 10μM Rho kinase inhibitor for the first 24h. Cells were grown for 7-10d until the appearance of visible colonies and stained for Alkaline Phosphatase activity (Sigma-Aldrich).

Crystal Violet staining
Media was removed and cells were fixed with 3:1 (v:v) methanol/acetic acid for 5 minutes. Fresh fix buffer added for a further 5 minutes and cells were washed once in water. Cells were dried for 30 minutes and then each well coated equally with 0.1% crystal violet solution in PBS for 10 minutes. Crystal violet solution was removed and the cells washed with water. Cells were then air-dried.

Immunofluorescent microscopy
ESC were seeded onto MEF-coated glass coverslips. Cells were fixed with 2% paraformaldehyde for 10 minutes at room temperature, washed in PBS then permeablised and blocked with 5% FBS, 0.1%Triton X-100 in PBS for 1 hour at room temperature. Cells were stained with primary antibody overnight at 4°C. Secondary antibody was applied for 4 hours at 4°C. Nuclei were stained with 0.2ng/ml DAPI (Sigma-Alrich). Images were acquired on an Olympus FV1000 or NIKON A1-R confocal microscopes at the Babraham Institute Imaging Facility.

Western blot
Whole cell lysates were extracted in RIPA buffer. Proteins were separated by electrophoresis in 10-15% SDS-polyacrylamide gels and transferred to 0.45μM PVDF membranes (Amersham Hybond). Membranes were blocked for >1 h in TBS-T 5% milk and hybridised to primary antibody overnight at 4°C. Membranes were washed 3 times for 10 minutes in TBS-Tween 5% milk at room temperature then incubated for 1 h at room temperature with secondary antibodies HRP-conjugated rabbit-anti-mouse or goat-anti-rabbit immunoglobulins (1:10,000 dilution, GE Healthcare). Detection was performed using ECL Primer Western Blotting Detection Reagent (Amersham).

RT-qPCR
Total RNA from bulk cells was isolated using the RNeasy kit (QIAGEN). Cells isolated by FACS went directly into TRIzol LS Reagent (Thermo Fisher Scientific) and total RNA was extracted followed by clean-up with the RNeasy kit (Qiagen). Total RNA (0.5-1μg) was reversed transcribed using the QuantiTect Reverse Transcription Kit (Qiagen). qPCR was performed using JumpStart Sybr Green (Sigma-Aldrich) in the following reaction: 6μl Sybr Green, 0.52μl water, 0.24μl 10mM forward primer, 0.24μl 10mM reverse primer, 250ng cDNA in a total volume of 12μl. The following primers were used: EZH2_qPCR ,EED_qPCR, SUZ12_qPCR, EZH1_qPCR, SOX17_qPCR, FOXA2_qPCR, GATA4_qPCR, GATA6_qPCR, SOX1_qPCR, NEUROD1_qPCR, OCT4_qPCR, NANOG_qPCR, SOX2_qPCR, HAND1_qPCR, TWIST1_qPCR, OLIG2_qPCR, SNAI1_qPCR, SNAI2_qPCR, PAX6_qPCR, GAPDH_qPCR and HMBS_qPCR.

RNA-Sequencing libraries
SSEA4-positive live ESC were isolated by FACS straight into TRIzol LS (Thermo Fisher Scientific). Total RNA was purified and then cleaned up using an RNeasy kit (Qiagen). Indexed mRNA-Seq libraries were constructed from 500ng total RNA using the Tru-Seq RNA Library Prep Kit v2 (Illumina). Library fragment size and concentration was determined using an Aglient Bioanalyzer 2100 and KAPA Library Quantification Kit (KAPA Biosystems). Samples were sequenced on Illumina HiSeq 1000/2500 as 100bp single-end libraries at the Babraham Institute Sequencing Facility.

For single-cell RNA-Seq experiments, individual SSEA4-positive live ESC were deposited by FACS directly into 96-well plates. Poly-A cDNA from single cells was obtained as described (Picelli et al., 2014). For library generation the Illumina Nextera DNA LT library preparation kit was used following the Fluidigm protocol described for cDNA generation using the Fluidigm C1 machine (available online). Pooled libraries were sequenced on an Illumina HiSeq

1000/2500 as 100bp paired-end libraries at the Babraham Institute Sequencing Facility. Spike-ins (ERCC RNA Spike-in Mix, Ambion) were added in a final dilution of 1:10E6.

RNA-Sequencing analysis
Reads were trimmed using trim galore v0.4 (http://www.bioinformatics.babraham.ac.uk/projects/trim_galore/) using default parameters to remove the standard Illumina adapter sequence. Reads were mapped to the human GRCh38 genome assembly using tophat 2.0.12 guided by the gene models from the Ensembl v70 release. BAM files were imported to Seqmonk v32.1 (http://www.bioinformatics.babraham.ac.uk/projects/seqmonk/). Raw read counts per transcript were calculated using the RNA-Seq quantitation pipeline on the Ensembl v70 gene set using non-directional counts. Differentially expressed genes were identified using DESeq2 with a cut-off of $p<0.05$ after multiple testing correction and without independent filtering. Correlation based hierarchical clustering of the DESeq2 hits was performed on log2 RPM (reads per million reads of library) values, which were normalised per-gene by subtracting the median value across all samples. Gene ontology (GO) functional annotation for the differentially expressed gene sets was done using the DAVID analysis tool (http://david.abcc.ncifcrf.gov/home.jsp). *P*-values were adjusted for multiple hypothesis testing using Bonferroni correction. Gene set enrichment analysis in Figure 3C was calculated using the GSEAPreranked tool within GSEA software (Subramanian et al., 2005) (www.broadinstitute.org/gsea). Input data were a ranked gene list ordered by fold-change expression between $EZH2^{-/-}$ ESC and $EZH2^{-/-\ +EZH2}$ ESC (n=20879), and a set of PRC2 targets (n=1299; defined by high EZH2 and H3K27me3 promoter-localised ChIP-Seq values in $EZH2^{+/+}$ ESC). Default settings were used with 1000 gene set permutations. The *EZH2* network in Figure 3E was analysed and visualized using Cytoscape (http://www.cytoscape.org). For Figure 3F, data were quantitated over 20bp running windows, separated by 10bp and the values were globally normalised to the data set with the highest coverage.

For single-cell RNA-Sequencing analysis, reads were trimmed using trim galore v0.4 (http://www.bioinformatics.babraham.ac.uk/projects/trim_galore/) using default parameters to remove the standard Illumina adapter sequence. Reads were mapped to the GRCh38 human genome assembly plus the ERCC spike-in control sequences using HiSat v2.0.1b guided by splice junctions imported from the gene models in Ensembl v78. Uniquely mapping reads were imported into SeqMonk v0.32.1 for analysis (http://www.bioinformatics.babraham.ac.uk/projects/seqmonk/). In initial QC, samples were removed where the percentage of reads coming from ERCC was >10% or the percentage of reads in genes was < 75% or the percentage of genes showing any signal was <15%. To remove potential ambiguity, samples were excluded if *EZH2* log2RPM > 1 in the $EZH2^{-/-}$ ESC set, and *EZH2* log2RPM < 2 in the $EZH2^{+/+}$ ESC set. Initial quantitation was performed on a gene set of annotated models with a biotype of protein_coding or lincRNA at a gene level counting reads overlapping any valid exon in each gene. Heatmaps were produced from hierarchical clustering of scaled RPM normalised single-cell RNA-Seq data using the R hclust function with the Ward's method and Euclidean distance correlations.

ChIP-Sequencing libraries
SSEA4-positive live ESC were isolated by FACS and snap frozen. Native ChIP for profiling histone marks was performed as described by Gilfillan and colleagues (Gilfillan et al., 2012) with minor modifications. Cell pellets (from 400,000 cells) were thawed and suspended in 95µl MNase Buffer (50mM Tris-HCl pH8, 1mM CaCl2, 0.2% Triton X-100) supplemented with 5mM Sodium Butyrate and 1X Complete EDTA-free Protease Inhibitor (Roche). Chromatin was digested with micrococcal nuclease (3U; NEB) for 8min at 37°C to obtain predominantly mononucleosomes and dinucleosomes. Stop buffer (10µl of 110mM Tris-HCl pH8, 5mM EDTA) was added to inactivate the reaction. Chromatin was solubilised by brief sonication using a Diagenode Bioruptor for 1min on high power. Chromatin was diluted in 100ml RIPA-IP buffer (280mM NaCl, 1.8% Triton X-100, 0.2% SDS, 0.2% Sodium Deoxycholate, 5mM EGTA supplemented with 5mM Sodium Butyrate and 1X Complete EDTA-free Protease Inhibitor) and insoluble material was removed by centrifugation at 14,000 rpm at 4°C for 15 minutes. Supernatant was transferred to a new tube, 10% was removed for the input sample, and the remaining chromatin was pre-cleared with 50µl pre-washed Protein A and Protein G Dynabeads (Thermo Fisher Scientific) at 4°C for 1 hour. Dynabeads were removed, the pre-cleared chromatin was diluted to 500µl in RIPA-IP buffer and split into 5 tubes. Chromatin was incubated overnight with histone antibodies at 4°C with rotation. Pre-washed Dynabeads (10µl) were added to each tube and incubated at 4°C for 2-3 hours with rotation. Beads were washed five times with RIPA buffer, once with LiCl buffer (250mM LiCl, 10mM Tris-HCl pH8, 0.5% NP-40, 0.5% Sodium Deoxycholate, 1mM EDTA) and once with 1xTE. Beads were suspended in 100µl 1X TE supplemented with 50µg Proteinase K and incubated at 55°C for 1 hour. ChIP and Input DNA were purified using Genomic DNA Clean and Concentrator columns (Zymo) and eluted in 50µl 1xTE. ChIP material was examined using a small aliquot of material by qPCR with primers ChIP_qPCR_MESP1, ChIP_qPCR_GAPDH, ChIP_qPCR_OCT4, ChIP_qPCR_TMEM255B, ChIP_qPCR_SOX17, ChIP_qPCR_GATA4 and ChIP_qPCR_Intergenic.

Crosslinked ChIP for profiling EZH2 occupancy was performed using iDeal ChIP-Seq kit for Transcription Factors (Diagenode).

Indexed ChIP-Seq libraries were generated with the NEBNext Master Kit (NEB) using NEBNext Multiplex Oligos for Illumina indexes (NEB). Library fragment size and concentration was determined using an Aglient Bioanalyzer 2100 and KAPA Library Quantification Kit (KAPA Biosystems). Samples were sequenced on Illumina HiSeq 1000/2500 as 100bp single-end libraries at the Babraham Institute Sequencing Facility.

ChIP-Sequencing analysis

Reads were trimmed using trim galore v0.4 (http://www.bioinformatics.babraham.ac.uk/projects/trim_galore/) using default parameters to remove the standard Illumina adapter sequence. They were mapped to the human GRCh38 genome assembly using bowtie2 v2.2.5 using default parameters. BAM files imported to Seqmonk and reads were extended by 200bp at their 5' end to approximate the true insert size. For Figure 2A, gene promoters were assigned with high, intermediate and low CpG states as previously described by Mikkelsen and colleagues (Mikkelsen et al., 2007). Sequence reads were quantitated over 100bp running windows and the values were globally normalised to the data set with the highest coverage. Quantitation trend plot was used to calculate the mean signal over an average gene body -/+ 5kb for each of the three promoter states. For Figure 2B, non-duplicated reads were quantified using globally normalised read counts within probes -/+ 2.5kb of annotated transcriptional start sites. From this set, $H3K27me3^{WT}$ promoters were defined as probes <0.1 log2 RPKM in input sample and >0.1 log2 RPKM in H2K27me3 IP sample. Probes were name-matched to genes and deduplicated by name. For Figure 3F and Figure S5, data were quantitated over 20bp running windows, separated by 10bp and the values were globally normalised to the data set with the highest coverage. For Figure S3B, 150Mb of Chromosome 5 (arbitrarily chosen) was divided into 300bp bins with 150bp step size, and the number of non-duplicated reads per bin was quantified and globally normalised to the data set with the highest coverage. For Figure 3, EZH2-target promoters were identified as follows: non-duplicated reads were quantified within probes -/+ 2.5kb of annotated transcriptional start sites, and probes with a globally normalised log2 read count between 7.25 and 10.5 were retained. Probes were name-matched to genes and deduplicated by name.

Antibodies

The following antibodies were used for flow cytometry: SSEA4 (R&D Systems, FAB1435), KDR-APC (R&D Systems, FAB357A), PDGFRα-PE (R&D Systems, FAB1264P), C-KIT-APC (Thermo Fisher Scientific, CD11705), CXCR4-PE (R&D Systems, FAB170B), EPCAM-APC (BioLegend, 324212), CD56-PE (BD Biosciences, 345812), Mouse IgG_{1}-APC (R&D Systems, IC002A), Mouse IgG_{2A} PE (R&D Systems, IC003P) and Mouse IgG_{I} PE (R&D Systems, IC002P). The following antibodies were used for immunofluorescent microscopy: SOX17 (R&D Systems, AF1924), OCT4 (Santa Cruz, sc5279), EZH2 C-terminal (Diagenode, pAB-039-050), EZH2 N-terminal (Sigma-Aldrich, E6909), H3K27me3 (Millipore, 07-449), H3K27me2 (Activ Motif, 39378), H3K27me1 (Activ Motif, 39246), H3S10ph (Millipore, 06-570), Donkey anti-goat AF488 (Thermo Fisher Scientific, A11055), Donkey anti-rabbit AF555 (Thermo Fisher Scientific, A31572), Goat anti-mouse AF568 (Thermo Fisher Scientific, A11031), Donkey anti-mouse AF647 (Thermo Fisher Scientific, A31571), Donkey anti-rabbit AF488 (Thermo Fisher Scientific, A21206). The following antibodies were used for ChIP: H3K27me3 (Millipore, 07-449; 1μg per ChIP), H4K4me3 (Abcam, ab8580; 0.5μg per ChIP), H3K27ac (Abcam ab4729; 0.5μg per ChIP), H3K4me1 (Abcam, ab8895; 0.5ug per ChIP), rabbit anti-mouse IgG (Jackson Immuno Research, 315-005-003; 0.5μg per ChIP) and EZH2 (Diagenode, pAB-039-050; 2μg per ChIP). The following antibodies were used for Western blotting: EZH2 (Diagenode, pAB-039-050), SUZ12 (NEB, 3737), EED (Millipore, 09-774), EZH1 (Abcam, ab13665), OCT4 (Santa Cruz, sc5279) and β-actin (Sigma-Aldrich, A5441).

Primers

EZH2_attb_F	GGGGACAAGTTTGTACAAAAAAGCAGGCTCTATGGGCCAGACTGGGAAGAA
EZH2_attb_R	GGGGACCACTTTGTACAAGAAAGCTGGGTCTCAAGGGATTTCCATTTCTCTTTCGA
mCherry_Geno_F	CTACGACGCTGAGGTCAAGA
mCherry_Geno_R	GTGTAGTCCTCGTTGTGGGA
TET-Prom_Geno_F	GCACGTCTCCCTATCAGTGA
TET-Prom_Geno_R	CCCGGTGTCTTCTATGGAGG
Ezh2_Exon7_F	TCAGCTTTGTTATAGAGACATAATTGG
Ezh2_Exon7_F	GGCTCATCCGCTACATTGAT
Ezh2_qPCR_F	GAGCAAAGCTTACACTCCTTTCA
Ezh2_qPCR_R	ATAAGTGTTGGGTGTTGCATGA
Suz12_qPCR_F	TTCTTCGAACTCGGAATCTCAT
Suz12_qPCR_R	TGATGTTTGTTCTGGAGTTTCG
Eed_qPCR_F	TTGCATTGGGCAATCAAGTT
Eed_qPCR_R	GCAGCACCACATTTATGATGAG
Ezh1_qPCR_F	GGCGCTGCTTTAAATACGACT
Ezh1_qPCR_R	CACATGGTTCTGGTTCAATCTT
Sox17_qPCR_F	CAGAATCCAGACCTGCACAAC
Sox17_qPCR_R	CTCTGCCTCCTCCACGAAG
Foxa2_qPCR_F	GTCCGACTGGAGCAGCTACTAT

Foxa2_qPCR_R	GTCCGACTGGAGCAGCTACTAT
Gata4_qPCR_F	GAAGCCCAAGAACCTGAATAAA
Gata4_qPCR_R	GTTGCTGGAGTTGCTGGAAG
Gata6_qPCR_F	GTGCCCAGACCACTTGCTAT
Gata6_qPCR_R	TGGAGTCATGGGAATGGAAT
Gapdh_qPCR_F	CGCTGAGTACGTCGTGGAGT
Gapdh_qPCR_R	GGGCAGAGATGATGACCCTTT
Hmbs_qPCR_F	AGGAGTTCAGTGCCATCATCCT
Hmbs_qPCR_R	CACAGCATACATGCATTCCTCA
Sox1_qPCR_F	ATGAAGGAGCACCCGGATTA
Sox1_qPCR_R	GCCAGCGAGTACTTGTCCTT
Pax6_qPCR_F	GGTTGGTATCCGGGGACTTC
Pax6_qPCR_R	CGTTGGAACTGATGGAGTTGGT
Neurod1_qPCR_F	CGAAGATGAGGACCTGGAAG
Neurod1_qPCR_R	CAGGCGAGCCTTAGTCATCT
Oct4_qPCR_F	GGATATACACAGGCCGATGTGG
Oct4_qPCR_R	ATGGTCGTTTGGCTGAATACCT
Nanog_qPCR_F	TCCAGCAGATGCAAGAACTCTC
Nanog_qPCR_R	GGTTCTGGAACCAGGTCTTCAC
Twist1_qPCR_F	CGGACAAGCTGAGCAAGATT
Twist1_qPCR_R	TGGAGGACCTGGTAGAGGAA
Hand1_qPCR_F	ACATCGCCTACCTGATGGAC
Hand1_qPCR_R	ATCCGCCTTCTTGAGTTCAG
Olig2_qPCR_F	GACAAGCTAGGAGGCAGTGG
Olig2_qPCR_R	CGGCTCTGTCATTTGCTTCT
Snai1_qPCR_F	TCAAGATGCACATCCGAAGC
Snai1_qPCR_R	CGGACATGGCCTTGTAGCAG
Snai2_qPCR_F	TCGGACCCACACATTACCTT
Snai2_qPCR_R	TGTGTCCTTGAAGCAACCAG
ChIP_qPCR_Intergenic_F	CGGATGACAGGGTTATTGCT
ChIP_qPCR_ Intergenic _R	GGATTCTGGGATCCTTGGTT
ChIP_qPCR_Gata4_F	TGAGAGGGTGTGCCAGAAACT
ChIP_qPCR_Gata4_R	GGGTTGCAAAGCGAATACATC
ChIP_qPCR_Sox17_F	AGTTGAGTCCTGGGGGAAAAA
ChIP_qPCR_Sox17_R	ACTGCATGGGCATCTTCAAAT
ChIP_qPCR_Mesp1_F	GTCTGCCAAGGAACCACTTC
ChIP_qPCR_Mesp1_R	CCCAAGTGACAAGGGACAAC
ChIP_qPCR_Oct4_F	CTCCCACACCTCCATGTTCT
ChIP_qPCR_Oct4_R	AGGAGCTGAGAGGGTGACTG
ChIP_qPCR_Tmem255b_F	CGGAATCTCCAATTCTTCCA
ChIP_qPCR_Tmem255b_R	AACTGGTGAACACGCAACAG
ChIP_qPCR_Gapdh_F	CAGGCTGGATGGAATGAAAG
ChIP_qPCR_Gapdh_R	AAAGGCACTCCTGGAAACCT

Supplemental References

Gilfillan, G.D., Hughes, T., Sheng, Y., Hjorthaug, H.S., Straub, T., Gervin, K., Harris, J.R., Undlien, D.E., and Lyle, R. (2012). Limitations and possibilities of low cell number ChIP-seq. BMC Genomics *13*, 645.

Golding, M.C., Zhang, L., and Mann M.R. (2010) Multiple epigenetic modifiers induce aggressive viral extinction in extraembryonic endoderm stem cells. Cell Stem Cell *6*:457-467.

Kennedy, M., D'Souza, S.L., Lynch-Kattman, M., Schwantz, S., and Keller, G. (2007). Development of the hemangioblast defines the onset of hematopoiesis in human ES cell differentiation cultures. Blood *109*, 2679-2687.

Lee, G., Chambers, S.M., Tomishima, M.J., and Studer, L. (2010). Derivation of neural crest cells from human pluripotent stem cells. Nat Protoc *5*, 688-701.

Mikkelsen, T.S., Ku, M., Jaffe, D.B., Issac, B., Lieberman, E., Giannoukos, G., Alvarez, P., Brockman, W., Kim, T.K., Koche, R.P.*, et al.* (2007). Genome-wide maps of chromatin state in pluripotent and lineage-committed cells. Nature *448*, 553-560.

Nostro, M.C., Sarangi, F., Ogawa, S., Holtzinger, A., Corneo, B., Li, X., Micallef, S.J., Park, I.H., Basford, C., Wheeler, M.B.*, et al.* (2011). Stage-specific signaling through TGFbeta family members and WNT regulates patterning and pancreatic specification of human pluripotent stem cells. Development *138*, 861-871.

Picelli, S., Faridani, O.R., Bjorklund, A.K., Winberg, G., Sagasser, S., and Sandberg, R. (2014). Full-length RNA-seq from single cells using Smart-seq2. Nat Protoc *9*, 171-181.

Subramanian, A., Tamayo, P., Mootha, V.K., Mukherjee, S., Ebert, B.L., Gillette, M.A., Paulovich, A., Pomeroy, S.L., Golub, T.R., Lander, E.S.*, et al.* (2005). Gene set enrichment analysis: a knowledge-based approach for interpreting genome-wide expression profiles. Proc Natl Acad Sci U S A *102*, 15545-15550.
